# Dopamine Partial Agonists in Pregnancy and Lactation: A Systematic Review

**DOI:** 10.3390/ph18071010

**Published:** 2025-07-06

**Authors:** Alexia Koukopoulos, Delfina Janiri, Miriam Milintenda, Sara Barbonetti, Georgios D. Kotzalidis, Tommaso Callovini, Lorenzo Moccia, Silvia Montanari, Marianna Mazza, Lucio Rinaldi, Alessio Simonetti, Mario Pinto, Giovanni Camardese, Gabriele Sani

**Affiliations:** 1 Department of Life Science, Health, and Health Professions, Link Campus University, 00165 Rome, Italy; a.koukopoulos@unilink.it (A.K.); g.camardese@unilink.it (G.C.); 2 Department of Psychiatry, Fondazione Policlinico Universitario Agostino Gemelli IRCCS, Largo Agostino Gemelli 8, 00168 Rome, Italy; milintenda@icloud.com (M.M.); sara.barbonetti@gmail.com (S.B.); giorgio.kotzalidis@gmail.com (G.D.K.); t.callovini@gmail.com (T.C.); lorenzomoccia27@gmail.com (L.M.); silvia.montanari@yahoo.com (S.M.); marianna.mazza@policlinicogemelli.it (M.M.); lucio.rinaldi@policlinicogemelli.it (L.R.); alessio.simonetti@policlinicogemelli.it (A.S.); mario.pinto@guest.policlinicogemelli.it (M.P.); gabriele.sani@unicatt.it (G.S.); 3 Department of Neuroscience, Section of Psychiatry, Università Cattolica del Sacro Cuore, Largo Francesco Vito 1, 00168 Rome, Italy; 4 Menninger Department of Psychiatry and Behavioral Sciences, Baylor College of Medicine, Houston, TX 77030, USA

**Keywords:** dopamine D_2_/D_3_ partial agonists, aripiprazole, brexpiprazole, cariprazine, pregnancy, lactation, breastfeeding, puerperium, perinatal period

## Abstract

**Background/Objectives**: Dopamine partial agonists are drugs initially developed to treat schizophrenia, seeking a double effect of increased dopaminergic transmission in the prefrontal cortex and decrease in the accumbens/striatum. Of these drugs, aripiprazole, brexpiprazole, and cariprazine are currently marketed and used in schizophrenia spectrum and mood disorders. It is debated whether patients with psychiatric disorders becoming pregnant should discontinue or continue their antipsychotic treatment despite some risks for the fetus, i.e., whether it is worse to have an untreated disorder or treating it with drugs. The safety of drugs for mother and baby extend from pregnancy to the postpartum, when breastfeeding assumes great importance. We set to investigate the use of dopamine partial agonists in pregnancy and lactation. **Methods**: On 23 June 2025, we used suitable strategies for identifying cases and studies of cariprazine, aripiprazole, brexpiprazole, dopamine partial agonists in pregnancy, perinatal period, and/or lactation on PubMed, CINAHL, PsycInfo/PsycArticles, Scopus, and ClinicalTrials.gov. We used the PRISMA Statement in developing our review. We included case reports and clinical studies. We excluded reports without pregnancy or focused on other drugs than the above. We reached consensus on eligibility with Delphi rounds among all authors. **Results**: Our searches produced 386 results on the above databases. We included 24 case reports/series and 15 studies. Most studies showed no negative pregnancy outcomes. There were serious concerns about the use of dopamine D_2_/D_3_ partial agonists during lactation. **Conclusions**: The use of dopamine partial agonists during pregnancy appears to be safe, but during breastfeeding they should be better avoided.

## 1. Introduction

There is considerable debate on whether pregnant women with psychiatric disorders, like schizophrenia and schizophrenia spectrum disorders, or mood disorders, like bipolar disorders or major depressive disorder, should continue their medication up to delivery (or even during lactation) or whether they should stop as soon as they become informed on being pregnant [1]. This debate stems from the possible dangerousness of the medications used for the above disorders, deduced either by animal studies of teratogenicity or by sporadic human reports of malformations or other adverse pregnancy/delivery outcomes. The latter have to be balanced against the occurrence of the same malformations and perinatal adverse outcomes occurring spontaneously in the general population with no prescription. Furthermore, pregnancy and postnatal outcomes of the fetus/infant may also relate to mother’s underlying psychiatric disorder and the symptoms displayed by the patient during her pregnancy [2]; in such case, stopping the medication might expose the patient to increased adverse outcome risks. Hence the risk of drug intake during pregnancy must be weighed against the risk of discontinuing the drug and exposing the patient to the risk of exacerbation of the underlying psychiatric disorder. Most women with psychiatric disorders decide to stop taking their medications upon knowing about their pregnancy [3], but with some psychiatric drugs, the teratogenic potential is higher when the exposure occurs during the first trimester [4], so when the patient decides to discontinue, the damage has already been done and the patient is further exposed to the potential damage of the underlying psychiatric disorder. Hence, clinicians are confronted with a very knotty question, whether to advise their patients persisting with medication intake or discontinue and adopt alternative measures. Such measures may involve exercise, psychoeducation, and lifestyle changes, as much of the impending dangers of the underlying psychiatric disorders involve personal life habits (substance use disorder and eating) [5] or metabolic syndrome [6].

Exposure to symptomatic schizophrenia or bipolar disorder during pregnancy carries more negative pregnancy outcomes than exposure to antipsychotic monotherapy treatment [7]. Generally, the use of antipsychotics throughout pregnancy is not associated with increased risk for adverse pregnancy outcomes in pregnant patients with schizophrenia spectrum or bipolar disorder, but with differences between the various antipsychotic classes and drugs, and each antipsychotic drug has its own risks, based on their chemistry [8]. Some classes, like the dibenzodiazepines, such as clozapine and olanzapine, have a higher dysmetabolic potential [9], while others, like the pyridopyrimidines risperidone and paliperidone (9-hydroxyrisperidone), and the substituted benzo amides amisulpride and sulpiride, thanks to their strong inhibitory activity of the D_2_ group of dopamine receptors of the tuberoinfundibular tract, impact the pituitary somatomammotrophs, thus causing hyperprolactinemia [10] and/or galactorrhea [11], hence impacting lactation [12]. Since dysmetabolism in pregnant women can lead to gestational diabetes, an adverse outcome per se, and also cardiocirculatory implications, ensuing in hypertension or even acute cardiac episodes, and hyperprolactinemia to deranged lactation, clinicians are seeking drugs that are devoid of such effects while maintaining their antipsychotic efficacy. Among the first-generation antipsychotic agents (FGAs) in clinical use, the phenothiazines, butyrophenones, and thioxanthenes are related to movement disorders and hyperprolactinemia [13], while the substituted benzo amides are potent inducers of prolactin secretion, thus impacting lactation [14]. A class of second-generation antipsychotics (SGAs) like the dopamine D_2_/D_3_ receptor partial agonists (DAPAs) is related to less extrapyramidal and other movement disorders save akathisia [15] and low likelihood to induce hyperprolactinemia [15,16].

**Figure 1:**
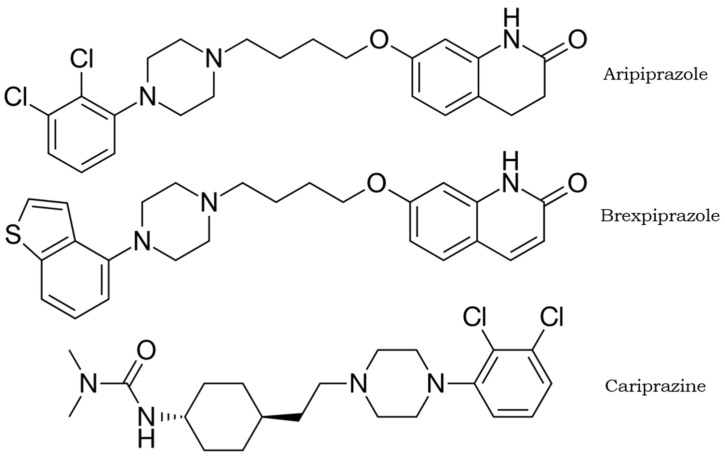
Chemical structures of DAPAs (aripiprazole, brexpiprazole, and cariprazine).

The three DAPAs currently marketed as antipsychotics are aripiprazole (7-{4-[4-(2,3-Dichlorophenyl)piperazin-1-yl]butoxy}-3,4-dihydroquinolin-2(1*H*)-one), brexpiprazole (7-[4-[4-(1-benzothiophen-4-yl)piperazin-1-yl]butoxy]quinolin-2(1*H*)-one), and cariprazine (*N’*-[*trans*-4-[2-[4-(2,3-Dichlorophenyl)-1-piperazinyl]ethyl]cyclohexyl]-*N,N*-dimethylurea), having in common a piperazinyl structure. Their chemical structures are shown in Figure 1.

The three DAPAs have peculiar receptor binding profiles that differ from other SGAs. However, they have also some differences among them [17,18,19,20,21]. These are shown in Table 1. The rationale for their use in the psychoses lies in the neurodevelopmental hypothesis of schizophrenia [17,18,19,20,21,22]; this postulates that there is a delay in migration of those neurons that would eventually become dopaminergic from the subplate [23,24] ensuing in hyperdopaminergic activity in the limbic system (septum, Calleja’s bodies, nucleus accumbens septi-ventral striatum and extended amygdala) that would produce positive symptoms (hallucinations and delusions) secondary to hypodopaminergism in the prefrontal cortex, with an impaired glutamatergic feedback to the striatum/accumbens that would produce negative symptoms (avolition, lack of curiosity and reduced ideation, asociality, blunted affect, anhedonia, alogia) [25]. Classical neuroleptics and also many SGAs lack the partial dopamine agonist activity of DAPAs, only having a dopamine antagonist activity that may be useful in the mesolimbic system, but harm dopaminergic activity in the prefrontal cortex, where it is needed to avoid the negative system. DAPAs, according to their developers’ wishes, are hypothesized to be able to maintain sufficient dopaminergic activity in the prefrontal cortex to counter negative symptoms, while effectively controlling dopaminergic mesolimbic activity [26].

**Figure 2:**
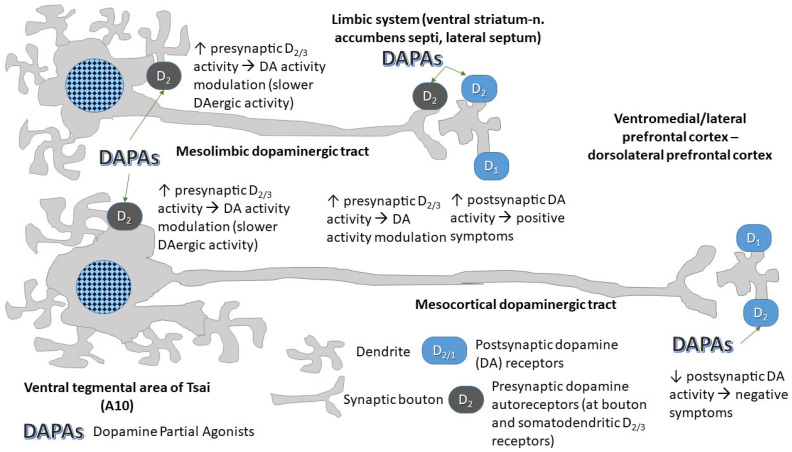
Mechanism of action of dopamine partial agonists. Ideally, these drugs displace dopamine and antagonists from the D_2_ group of dopamine receptors (D_2_, D_3_, and D_4_), thus moderately enhancing the action of dopamine at this level. The consequence of this action in the site of origin of dopaminergic neurons that project to the cortex and the limbic system (ventral tegmental area {A10} and retrorubral area {A8}) is that dopamine synthesis and forwarding to the synapse is slowed down. The presynaptic action at the mesolimbic level ensues in smoothly modulating dopamine release, while the partial agonist activity partially counteracts the excess dopamine activity at that level, which is linked to production of positive symptoms, such as hallucinations and delusions. At the prefrontal cortical level, where there is a relative lack of dopamine autoreceptors, where the decreased baseline dopamine activity is believed to be related to intellectual numbness, lack of curiosity, and low drive for pleasurable activity, partial dopamine agonists increase the dopaminergic stimulation of these receptors, accounting for their efficacy against negative symptoms. Of course, this may only be wishful thinking.

The mechanism of action of DAPAs is believed to be linked to their partial dopamine receptor agonist activity at lower-intermediate doses, while increasing their doses leads to pure antidopaminergic activity [26,40]. The mode of action of DAPAs is illustrated in Figure 2. Briefly, at lower dosages, DAPAs activate D_2/3_ somatodendritic autoreceptors in the A10 area, i.e., the origin of most dopaminergic projections to the forebrain. This results in the downregulation of dopamine synthesis, which may be good for the mesolimbic terminations, but bad for the dorsolateral prefrontal cortex (DLPFC) terminations, in that there, dopaminergic activity is already low, thus ensuing in negative symptoms. However, this latter area is endowed with postsynaptic D_2_ receptors while relatively lacking terminal autoreceptors [41], hence, the end result of the administration of DAPAs is the partial activation of these receptors, whose activity is much needed to counteract the negative symptoms of the psychoses, like anhedonia, alogia, avolition, blunted affect, and social withdrawal. In the mesolimbic terminations (nucleus accumbens septi-ventral striatum, extended amygdala and lateral septal nuclei), the partial agonist activity achieves to limit the action of excess dopamine at a postsynaptic level, while the partial agonist activity at the level of the presynaptic terminal (where the receptors of the D_2_ group are mainly constituted by D_2_ and D_3_ dopamine receptors) modulates the release of dopamine at that level (Figure 2).

Based on their pharmacological properties, DAPAs may be useful to treat psychoses during pregnancies. However, their teratogenic potential has not been extensively evaluated in the human [42]. Animal studies are lacking for brexpiprazole and cariprazine, while aripiprazole was shown to induce placental atrophy and dysgenesis in pregnant female rats [43] and delayed neurogenesis and development in early chick embryos [44]. Since currently available data are insufficient to determine the teratogenic and malformation potentials of DAPAs, we decided to undertake a review of potential adverse and lactation outcomes of these drugs, i.e., aripiprazole, brexpiprazole, and cariprazine, by searching the dedicated medical literature.

## 2. Materials and Methods

To investigate the pregnancy and lactation outcomes of DAPAs we searched the PubMed, CINAHL, PsycInfo/PsycArticles databases on 23 June 2025, using the following search strategy: (cariprazine OR aripiprazole OR brexpiprazole OR “dopamine partial agonist *”) AND (pregnant OR pregnancy OR lactation OR breastfeeding OR peripartum OR postpartum OR perinatal). Furthermore, we searched the Scopus database using TITLE(cariprazine OR brexpiprazole OR aripiprazole) AND TITLE-ABS-KEY(pregnancy OR lactation OR breastfeeding) and the ClinicalTrials.gov register on the same day. The searched produced the results shown in the Supplementary Table S1. No language or time restrictions were applied; all databases were searched since their inception, although we did not expect to find any result before the first introduction of aripiprazole.

Eligibility was determined by all participating authors, who discussed it through Delphi rounds. Not more than two were required to obtain consensus on eligibility. We applied the PRISMA 2020 Statement in conducting our review [45]. We filled-out the PRISMA checklist in Supplementary Table S2.

**Figure 3:**
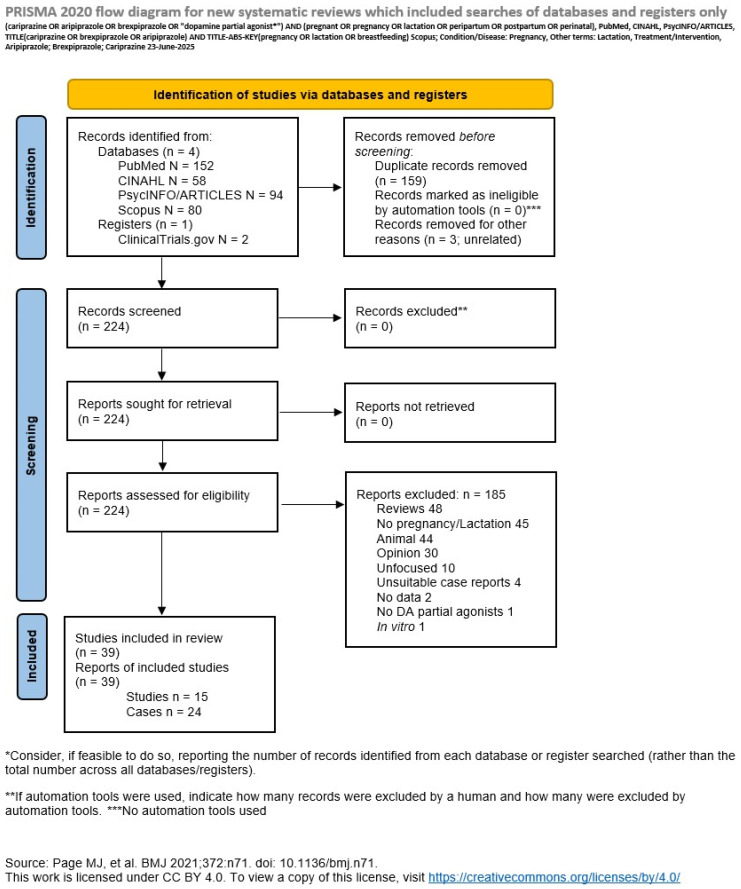
PRISMA 2020 flowchart [45] of our search with reasons for exclusion.

Eligible were studies or case reports dealing with the administration of DAPAs (aripiprazole, brexpiprazole, and cariprazine) in pregnant women. Excluded were reviews (labeled Review; however, we retrieved them and hand-searched them for possible eligible papers that could have been missed by our research strategy), editorials or letters to the editor expressing the author’s opinions but not providing data, collectively labeled as Opinion, studies that were included in the search but not dealing with pregnancy outcomes or lactation, termed No pregnancy/lactation, and those which, despite including pregnant women, lumped their results with those of other populations (termed Lumping). Articles not specifically targeting the pregnancy/lactation outcomes were labeled as Unfocused, and studies resulting in the search but were unrelated to the subject matter, labeled Unrelated. We also excluded studies not using DAPAs, labeled as No DA partial agonists, those that did not provide suitable data, labeled No data, and in vitro studies, labeled In vitro. When a case report/series provided unsuitable data was labeled Unsuitable case and excluded. We would also have excluded retired or retracted papers and those that present data on the same samples, termed as Overlapping, keeping only the best quality of them, or studies presenting the same data or corrections to an existing article, termed Duplicates; however, no such categories emerged. We show the selection process in Figure 3, where we present the PRISMA flowchart of our search.

### Quality and Risk-of-Bias Assessment

We investigated the quality of the case reports using the JBI Critical Appraisal Checklist for Case Reports [46,47]. We also assessed the risk of bias of the included clinical studies with the Cochrane Risk-of-Bias (RoB-2) tool [48]. Detailed results for these measures are shown in the Supplement, Supplementary Table S3.

We registered our review on the Open Science Framework (OSF) platform, with the registration Identifier DOI: 10.17605/OSF.IO/KGNCA.

## 3. Results

Our PubMed search on 23 June 2025, i.e., (cariprazine OR aripiprazole OR brexpiprazole OR “dopamine partial agonist*”) AND (pregnant OR pregnancy OR lactation OR peripartum OR postpartum OR perinatal), produced 152 results, of which 36 were eligible. Of them, fifteen were studies (eleven from databases [retrospective], one with a small sample, one longitudinal, and two prospective) and twenty-one were case reports/series. Excluded were thirty-four reviews, thirty-four animal studies, eighteen articles not involving pregnancy and/or lactation-breastfeeding, eleven opinion papers, nine studies which had a design unable to meet our demands and were labeled unfocused, one for being an unsuitable case report, one not involving any DAPA, one in vitro, one providing no data, and one unrelated to the subject matter, while two were duplicates, i.e., referring to another record resulting in the list, in both cases corrections (errata corrige). The CINAHL search added fifty-eight records, of which two were case reports to add to the PubMed search, while the PsycINFO/PsycARTICLES searches added ninety-four articles, adding a further case report. The Scopus search added eighty records with no more eligible articles, while ClinicalTrials.gov produced just one record regarding the National Pregnancy Registry for Psychiatric Medications, with a code NCT01246765 that has different occurrences in PubMed, but is still recruiting. Most of the additional databases produced duplicates. Detailed results are shown in Supplementary Table S1.

### 3.1. Case Reports/Series

The case reports we used in our review were 24 [49,50,51,52,53,54,55,56,57,58,59,60,61,62,63,64,65,66,67,68,69,70,71,72]. Their summary is shown in Table 2. They corresponded to thirty-three patients described, mostly affected by disorders of the schizophrenia spectrum, but also eight cases of bipolar disorder and one with anxiety and depression. There were six cases whose ethnicity/race was unreported.

### 3.2. Clinical Studies

The clinical studies we included in this review were 15 [73,74,75,76,77,78,79,80,81,82,83,84,85,86,87]. The summary of these studies is shown in Table 3. Most were country-based, but there were also two that used international databases (the World Health Organization [WHO] pharmacovigilance system). The countries involved were the US (six studies), Japan (two studies), and the Czech Republic, France, Norway, Australia, and India (one study each).

The details of the search strategy and inclusion/exclusion decision, with the reasons for exclusion, are shown in the Supplementary Table S1 and in Figure 3, where the PRISMA flow-chart is provided.

The eligible studies included a total of 5,318,184 women and one study included only neonates, with 6208 cases of reported anomalies [85]. The number of people involved in these studies appears to be extremely large; however, other investigators reported on already defined malformations, whatever treatment they were undergone, while others, those with the smallest samples, focused on patients who were exposed to DAPAs (essentially, aripiprazole). Given the wide variation of study designs, a meta-analysis was unfeasible. Of the twenty-four case reports/series, nineteen were favorable to the continuous administration of aripiprazole during pregnancy (no reports on brexpiprazole and cariprazine), and three reported obstetric complication and adverse developmental outcomes (one transient), but there were eight reports of negative effects on lactation and only one reported breastfeeding to be unaffected by aripiprazole [52]. Of the clinical studies, one reported pregnancy to lower serum concentrations of aripiprazole, but did not report other negative outcomes [75], while six were positive for aripiprazole and seven had some concerns, but none showed increased teratogenicity. One showed definitely that aripiprazole suppresses lactation [87].

## 4. Discussion

This systematic review showed that aripiprazole and brexpiprazole (from just one study) carry some potential for adverse neonatal outcomes and are detrimental to milk production and other measures related to breastfeeding, including mother and infant-related factors. Cariprazine had no reports, either case/series or study, while the bulk of evidence came from aripiprazole studies. However, the evidence is not clear-cut, and is still interlocutory. There is need for more data to clarify whether DAPAs should be continuously prescribed during pregnancy. Case reports were more positive than studies attempting at identifying a “signal” through disproportionality analyses. Taken together, our review suggests caution with administration of DAPAs in pregnant women and discontinuing the drug during breastfeeding or shifting to other means of lactating the neonate if one is to persist in DAPA intake.

The data collected here for the DAPAs were not specifically directed at obtaining data for this group of drugs during pregnancy. This limits the strength of the results; in fact, most studies are interlocutory and even inconsistent, with the DAPA group indistinguishable at times from other antipsychotics [74,75,84], and sometimes faring better [85] and other times worse [82]. While we would not recommend suspending the administration of antipsychotic drugs, including DAPAs in women with severe underlying psychiatric disorders, the picture changes when it comes to lactation [88]. Although recent reviews have been reassuring [89,90], aripiprazole and cariprazine, among other antipsychotics, were shown to inhibit dehydrocholesterol-reductase 7, thus resulting in accumulation of the immediate cholesterol precursor, 7-dehydrocholesterol, which is highly unstable and results in the formation of toxic oxysterols, which may also be teratogenic [91], but this is not limited to DAPAs and extends to other antipsychotics. These products interfere with glial cholesterol synthesis [92] and may affect embryonic development [93]. This is only indirect evidence, mostly speculative, but should induce a cautious approach in clinicians dealing with the problem of whether to suggest alternative ways of milking to mothers on DAPAs.

Lactation involves the oxytocin-mediated ejection of milk through a reflex upon suckling, which induces the production of prolactin by pituitary somatomammotrophs [94]. Both mother- and newborn-related factors may affect the production and the ejection of milk and its absorption by the neonate, thus the efficacy of breastfeeding. Defective latching, like in the case of cleft lip, may impair lactation in patients on aripiprazole [95], Likewise, reduced oxytocin and prolactin production may be followed by milk failure; DAPAs, particularly aripiprazole, were related to decreased prolactin production [16,96,97], but in some people with particular genetic characteristics, also to hyperprolactinemia [98], while brexpiprazole [99] and cariprazine [100] were not associated with significant variations in prolactin.

Taking together all these reports, inconsistencies exist in assessing the teratogenic/malformation potential of all DAPAs, but there is consensus on the need to continue medication once it started throughout pregnancy and avoid it during the postpartum, when lactation and breastfeeding are under way. All decisions must be taken in agreement with the adequately informed patient [67] and/or significant others, if she cannot provide informed consent due to incapacity. The future employment of DAPAs during pregnancy and lactation has to await further experience and comparison with other antipsychotics, always balancing risks to benefits.

Pregnancy was reported to lower aripiprazole levels [75], but this has not reported heretofore for other DAPAs. If such event occurs, it is possible that dosage adjustments are needed. From our case reports, it emerges that there is much interpersonal variability in response to these drugs during pregnancy, so each case should be dealt individually, pointing to personalized medicine interventions aided by pharmacogenomics and therapeutic drug monitoring (TDM). TDM revealed an increased metabolism of aripiprazole in later pregnancy, namely the third trimester, that should prompt to adjust doses [78]. TDM is a very useful tool during pregnancy to ensure safety, thus preventing harm to both mother and fetus [101]. TDM has been applied in studies of aripiprazole [102], brexpiprazole [70], and cariprazine [103]. The content of aripiprazole and its major metabolite have also been measured in both serum and milk of the mother, rendering it possible to measure how much drug the newborn may ingest [60]. We welcome an extension of TDM to pregnant and lactating mothers.

### Limitations

This review obtained limited data due to the fact that the field is relatively new. However, besides PubMed, we used three other databases and the ClinicalTrials.gov registry, although the latter provided just one unyielding record. Furthermore, we did not investigate in detail the risk of bias of included papers, but we were aware during the revision and reporting that there was a low risk of bias for all eligible. The Rob-2 produced low risk of bias for all studies, while the quality of all included studies was good (Supplemental Table 3). We investigated for a sponsor effect, but there was none among the case reports, which were the most favorable towards DAPAs. Also among clinical studies, there was no apparent interference of drug manufacturers; the only study that had a design with excessive focus on one drug [86] was not financially supported by the pharmaceutical industry and none of the authors was affiliated with any pharmaceutical company. The strength of our review is its originality; the weakness concerns a “dilution” effect when the obtained data have to be seen against the background of other antipsychotics for which very large samples are available. We should wait for the collection of pregnancy/lactation data for DAPAs of sufficient participant numbers to overcome this weakness.

## 5. Conclusions

DAPAs (data mainly from aripiprazole, limited from brexpiprazole, and none for cariprazine) carry a small potential for adverse obstetric or neonatal outcomes, but may suppress milk production and complicate breastfeeding. Data from case reports/series were more positive than studies searching databases. Taken together, our review suggests caution in prescribing DAPAs to pregnant women. During pregnancy, discontinuing the medication is not suggested, but monitoring serum levels of DAPAs might provide clues as to dose adjustments. However, it is suggested to discontinue DAPAs during breastfeeding or giving up breastfeeding if DAPA intake is intended to continue.

## Figures and Tables

**Table 1:** Receptor binding/affinity profiles of three marketed DAPAs, aripiprazole, brexpiprazole, and cariprazine.

	Aripiprazole		Brexpiprazole		Cariprazine		Haloperidol	
Site	*K*_D_/*K*_i_ (nM)	Action	*K*_D_/*K*_i_ (nM)	Action	*K*_D_/*K*_i_ (nM)	Action	*K*_D_/*K*_i_ (nM)	Action
SERT	98–1080	Inhibitor			65% at 10 μM	Inhibitor		
NAT	2090	Inhibitor			0% at 10 μM	Inhibitor		
DAT	3220	Inhibitor			90% at 10 μM	Inhibitor		
5-HT_1A_	1.7–5.6	Partial agonist	2.6	Partial agonist	0.12	Partial agonist	1927	Agonist
5-HT_1B_	830	ND			32	ND		
5-HT_1D_	68	ND			0.47	Antagonist		
5-HT_1E_	8000	ND			1.9	Antagonist		
5-HT_2A_	3.4–35	Antagonist	18.8	Antagonist	12–34	Antagonist	53	Antagonist
5-HT_2B_	0.11–0.36	Inverse agonist	0.58	Antagonist	140	ND		
5-HT_2C_	15–180	Partial agonist	134	Inverse agonist	58	Antagonist	<10,000	Antagonist
5-HT_3_	628	ND			3.7	Antagonist		
5-HT_5A_	1240	ND			0.12	Partial agonist		
5-HT_6_	214–786	Antagonist			32	ND	3666	Antagonist
5-HT_7_	9.6–39	Antagonist	84.1	Antagonist	0.47	Antagonist	377.2	Antagonist
α_1A_	25.9	ND	155	Antagonist	3.8	Antagonist	12	Antagonist
α_1B_	34.4	ND			0.17	Antagonist		
α_1D_					2.6	Antagonist		
α_2A_	74.3	ND			15	Antagonist	1927	Agonist
α_2B_	102	ND			17	Antagonist	480	Agonist
α_2C_	37.9	ND			0.59	Antagonist	550	Agonist
β_1_	141	ND			59	Antagonist		
β_2_	163	ND			67	Antagonist		
β_3_					>10,000	ND		
D_1_	265–1170	ND			160	ND	45	Antagonist
D_2_	1.4	Partial agonist			0.35	Partial agonist	0.7	Inverse agonist
D_2L_	0.74–1.2	Partial agonist	0.49	Partial agonist	0.30	Partial agonist	0.7	Inverse agonist
D_2S_	1.2	Partial agonist	0.69	Partial agonist			0.7	Inverse agonist
D_3_	0.8–9.7	Partial agonist	0.085	Partial agonist	1.1	Partial agonist	0.2	Inverse agonist
D_4_	44–514	Partial agonist			6.3	ND	5–9	Inverse agonist
D_5_	95–2590	ND			ND	ND	?	Antagonist
H_1_	27.9–61	ND	23.2	Antagonist	19	Antagonist	1800	Antagonist
H_2_	>10,000	ND			>10,000	ND		
H_3_	224	ND			>10,000	ND		
H_4_	>10,000	ND						
mACh			>1000	Antagonist	52% at 10 μM	ND	>1000	Antagonist
M_1_	6780	ND			67% at 10 μM	ND	>10,000	Antagonist
M_2_	3510	ND			>10,000	ND		
M_3_	4680	ND						
M_4_	1520	ND						
M_5_	2330	ND						
NMDA	4001	Antagonist					2000 IC_50_ *	Antagonist
σ					96% at 10 μM	ND		
σ_1_							3	Antagonist
σ_2_							54	Agonist

Note. The higher the *K*_i_ values, the lower the binding affinity; % potency of receptor occupancy at 10 micromolar concentrations increases as values increase. Blank boxes, not tested. Abbreviations. D, dopamine receptors; DAT, dopamine transporter; H, histamine receptors; *K*_i_, dissociation constant, inhibitory; M, muscarinic cholinergic receptors; NAT, noradrenaline transporter; ND, not determined; nM, nanomoles; NMDA, *N*-methyl-D-aspartate glutamate receptors; SERT, serotonin transporter; 5-HT, serotonin receptors; α, alpha adrenoceptors; β, beta-adrenoceptors; μM, micromoles; σ, sigma chaperone intracellular receptors (1 and 2 subtypes). Based on Shapiro et al. (2003) [27] for aripiprazole; Maeda et al. (2014) [28,29] for brexpiprazole; Kiss et al. (2010) [30], Herman et al. (2018) [31] for cariprazine, and Kanba et al. (1994), Leysen et al. (1992), Malmberg et al. (1998), Leysen et al. (1994), Cobos et al. (2007), Colabufo et al. (2004), Kroeze et al. (2003), Ilyin et al. (1996) [32,33,34,35,36,37,38,39] for haloperidol. * NR1/NR2B subunit, ifenprodil site.

**Table 2:** Summary of clinical case reports/series of patients receiving dopamine partial agonists during pregnancy and/or lactation in chronological order.

Study	Patient (s)	Treatment	Outcomes
Mendhekar et al., 2006 [49]	♀ 22 yrs, Indian, paranoid SCZ	No drugs for 1 yr before pregnancy. At GW 29, the pt had a recurrence of psychosis and was started on aripiprazole, increased to 15 mg/day over two wks. Symptoms resolved after 8 wks, but treatment continued until 6 days before delivery, totaling 85 days of exposure.	At 37 wks spontaneous labor; she delivered a healthy ♂weighing 2.6 kg; Apgar scores of 9 at 1 min and 10 at 5 min. At 6 months, the infant remained in good health.
Mendhekar et al., 2006 [50]	♀ 27-yrs, Indian, schizoaffective disorder	Became pregnant while taking 15 mg/day oral aripiprazole Medication discontinued at 8 wks of pregnancy. She experienced a relapse at 20 GWs and restarted aripiprazole at 10 mg/day. She responded well to treatment; medication was maintained for the remaining of the pregnancy.	The pregnancy progressed without complications, although unexplained fetal tachycardia led to C-S. The newborn was a healthy ♂, weighed 3.25 kg, and showed normal development over a 6-month FU. Bottlefeeding necessary due to unsuccessful lactation.
Mervak et al., 2008 [51]	♀ 24-yrs, Caucasian?, schizoaffective disorder	The pt had been taking aripiprazole 20 mg/day but discontinued it around the time of conception. At around 8 GWs, she experienced symptom relapse. Aripiprazole was gradually reintroduced, titrating back to the original dose of 20 mg/day. Her symptoms significantly improved; she continued on the same dose of medication throughout the rest of her pregnancy. At 39 wks, she presented with mildly elevated blood pressure (149/95) but showed no signs of preeclampsia.	≈40 wks, she gave birth uneventfully to a healthy ♂ weighing 3.24 kg, with Apgar scores of 9 at both 1 and 5 min. She chose not to breastfeed, and both mother and child remained healthy at FUs up to 12 months postpartum.
Lutz et al., 2010 [52]	♀ 34 yrs, Caucasian?, paranoid SCZ	In 2006, the pt conceived while on aripiprazole, which was then stopped; she delivered a healthy baby in early 2007. After a relapse in August 2007, she resumed aripiprazole and responded well. During her 2nd pregnancy in 2008, aripiprazole (15 mg/day) was continued to prevent relapse.	She delivered a healthy ♂ in February 2009, with no observed abnormalities. Although ongoing medication, aripiprazole and its metabolite were undetectable in breast milk samples, and the estimated infant exposure was below 0.7%; both mother and infant remained healthy over a 3-month FU.
Nguyen et al., 2011 [53]	♀ 27 yrs, Caucasian?, SCZ	The pt stopped aripiprazole 10 mg and citalopram 20 mg at 5 wks pregnant due to concerns about fetal safety. At 16 wks, early relapse symptoms appeared, and she resumed both medications after a risk-benefit discussion. Her condition improved, but she stopped both drugs again without medical advice after 5 wks. She restarted aripiprazole at 36.7 wks due to postpartum relapse risk but did not resume citalopram.	She had an uncomplicated pregnancy and delivered a healthy ♀ via elective C-S at 39.3 wks due to breech presentation. She participated in a placental transfer study showing cord:maternal serum ratios of 0.64 for aripiprazole and 0.47 for its metabolite DHAri (assessed through LC). The newborn had mild, self-resolving respiratory distress and brief feeding difficulty, with normal neonatal assessments. The mother remained psychiatrically stable postpartum and chose not to breastfeed.
Watanabe et al., 2011 [54]	♀ 27 yrs, Japanese, diagnosed with SCZ at GW 21	The exact duration of her symptoms was uncertain, but she had been receiving welfare due to long-standing social and work-related difficulties. At 22 wks of pregnancy, she was prescribed aripiprazole at 6 mg/day, which helped reduce her SCZ symptoms. However, since her delusions and hallucinations persisted, the dosage was raised to 12 mg/day at 30 wks and then to 18 mg/day at 34 wks. The 18 mg/day dosage was kept steady thereafter.	At GW 35 external cephalic version for breech presentation attempted but failed. The pt delivered a healthy 2866 g ♂ via scheduled C-S at 38 wks. The newborn showed no morphological abnormalities, though Apgar scores were initially low (2 at 1 min, 9 at 5 min). He required 1 min of respiratory support due to poor tone and no spontaneous breathing, but recovered well. The mother, on aripiprazole 18 mg/day, chose to stop breastfeeding on day 6 due to fatigue. Both mother and baby were in good health at the 2-month FU. LC measured aripiprazole concentrations in umbilical vein blood, maternal blood, neonatal blood at 6 days, and breast milk at 6 days after C-S. The levels were 96.4, 181, 7.6, and 38.7 ng/mL, respectively.
Gentile et al., 2011 [55]	♀ 36 yrs, Italian (Caucasian?), chronic delusional disorder	The pt stopped aripiprazole 15 mg/day in October 2009 to pursue pregnancy and conceived the following month. At 14 GWs, she experienced a severe psychotic relapse. Aripiprazole was restarted at 10 mg/day and continued successfully until delivery.	The pt had a scheduled C-S on 16 August 2010 (due to psychiatric history) delivering a healthy ♀. The newborn weighed 3400 g, measured 49 cm, and had Apgar scores of 9 and 10. Pregnancy was smooth, with normal lab tests and no fetal anomalies (aripiprazole was restarted post-organogenesis). The mother opted not to breastfeed, and the infant grew and remained healthy at 8-wk FU.
Widschwendter and Hofer, 2012 [56]	♀ 36 yrs, Caucasian?, paranoid SCZ	In September 2009, while still antipsychotic-naïve, the pt delivered a healthy ♂. In 2010, she became pregnant again while on aripiprazole 15 mg/day. Plasma levels at GWs 6 and 11 were 163.5 and 131 ng/mL, respectively. She chose to stop aripiprazole at GW 11 due to concerns for the fetus.	After an uneventful pregnancy, she gave birth to a healthy ♀ in March 2011, with no abnormalities detected. Post-delivery, she chose not to breastfeed and resumed treatment with aripiprazole 15 mg/day. 1-yr later, pt remained in remission and the child’s development was normal.
Wakil et al., 2013 [57]	♀ 31 yrs, G2P0010, Caucasian?, psychosis and BD	During 1TM the pt discontinued aripiprazole, without medical advice, due to concerns for fetal health. At GW 37, she presented to the ED with fluid leakage concerns, but no rupture of membranes was found. She exhibited severe agitation and psychosis, requiring restraints and antipsychotic medications. Upon psychiatric admission, she was treated with olanzapine, valproate, haloperidol, lorazepam, and diphenhydramine. Her symptoms gradually improved with this regimen	At GW 41, after a failed labor induction, she underwent C-S. The baby was born healthy, weighing 4385 g, with Apgar scores of 8 and 9 at 1 and 5 min, respectively (sex/gender not provided). Ms. M chose not to breastfeed due to concerns about the psychotropic medications. She was on valproic acid and haloperidol at the time of delivery. She was stable at discharge on postpartum day 8 with outpatient FU at a specialized clinic. She was prescribed valproic acid (2000 mg qHS), aripiprazole (20 mg/day), and haloperidol (5 mg qHS).
Windhager et al., 2014 [58]	**M1**: ♀ 32 yrs, Caucasian, schizoaffective disorder **M2-1/M2-2**: ♀ 32 yrs (her 2nd and 3rd pregnancy), Caucasian, SCZ	**M1** continued aripiprazole 15 mg daily after pregnancy confirmation at GW 9, with an increase to 20 mg in the last 2 months due to relapse concerns. **M2-1** stopped aripiprazole (20 mg) at GW 6, but due to relapse risk, restarted at 10 mg in GW 14, increasing to 15 mg at GW 30. **M2-2**, during her 3rd pregnancy, tapered from 15 mg to 5 mg and discontinued between GW 4–13. She restarted at 5 mg, titrated to 7.5 mg at GW 21, and continued until delivery. All pts showed a significant decline (>⅔) in aripiprazole PLs during pregnancy (assessed through LC), especially at 3TM, prompting dose adjustments. PLs dropped further before birth but rebounded postpartum, doubling within 4–5 wks.	All three pregnancies ended in full-term, spontaneous deliveries with no complications. Newborn adaptation was smooth, with Apgar scores of 9/10/10 (both). Sex/gender at birth not declared. **M1** remained in stable psychiatric remission for 16 months postpartum (end of observation). **M2-1** had no psychotic symptoms during a 6-month FU and stayed stable between her 2nd and 3rd pregnancies. **M2-2** increased her aripiprazole dose postpartum up to 15 mg to prevent relapse and remained stable during a 7-month FU. Both mothers chose not to breastfeed. Fetal aripiprazole levels at birth were 54, 44, and 35 ng/mL respectively, with a mean placental transfer rate of 54.67%.
Pirec et al., 2014 [59]	**M1:** ♀ 26 yrs, Israeli, BD with psychotic features. **M2:** ♀ 23 yrs, Israeli, BD with psychotic features	**M1**: Pt discontinued aripiprazole 15 mg/day at 4 wks GA upon pregnancy discovery. Lamotrigine was added, titrated up to 150 mg/day at 25 weeks GA. At 31 weeks GA, she had a manic psychotic relapse and was hospitalized; lamotrigine was stopped and clonazepam + haloperidol introduced. Upon discharge: aripiprazole 10 mg BID and clonazepam 1 mg QHS. **M2**: Patient hospitalized at ~6 weeks GA with psychotic mania and polysubstance use. Initially treated with ziprasidone and haloperidol, later switched to aripiprazole (titrated to 25 mg/day) and Li^+^ (300 mg TID). Improved by 10 wks GA, discharged stable. Self-discontinued Li^+^ at 12 wks GA due to sedation. Continued aripiprazole 25 mg/day monotherapy.	**M1**: Delivered infant (sex not mentioned) at term via C-S for breech presentation. Infant in 5–10th percentile, Apgar scores 9/9. NICU admission on day 2 due to poor feeding and hyperbilirubinemia, resolved spontaneously. Discharged on day 5. Bottle-fed. At 3-month follow-up: good development; umbilical hernia and thigh hemangioma noted. **M2**: Delivered ♂ infant at term, vaginal delivery. Apgar scores 9/9. Infant: 26th percentile weight, 53rd percentile length, <3rd percentile head circumference, with hypospadias. Bottle-fed. Microcephaly possibly due to polypharmacy.
Nordeng et al., 2014 [60]	♀ 35 yrs, BD-I, Norwegian	Pt developed postpartum psychosis at her first pregnancy, 13 years ago. Two further pregnancies were followed by mild postpartum depressive symptoms. At her current pregnancy she was on lamotrigine 50 mg/day and aripiprazole 10 mg/day. They were both discontinued but aripiprazole reintroduced due to manic relapse at GW 9. Manic symptoms subsided; pt remained stable thereafter.	She delivered at GW 39 and 4 days a healthy ♀, which she wished to breast-feed. She did so while on 10 mg/day aripiprazole. Her drug and DHAri milk levels were measured through LC-MS at wks 8 and 10 postpartum. Based on these measures, the baby was assumed to ingest ≅20 µg/day aripiprazole with mother’s serum levels being 114 ng/mL. Pt’s serum prolactin levels were lower than most lactating women’s levels (35–40 ng/mL vs. 50–100 ng/mL), resulting in insufficient milk levels. However, the baby developed normally and had no side effects.
Frew, 2015 [61]	♀ 34 yrs, G2P2, Caucasian?, BD-I	Pt had one hospitalization for mania during early pregnancy. Treated with Li^+^ (600–900 mg/day) during the 2TM and 3TM with excellent symptom control.	The pt gave birth to a healthy, full-term baby (sex not disclosed) and chose to continue Li^+^ (600 mg/day) while breastfeeding. At day 10, the infant’s Li^+^ level was 0.26 mmol/L (58% of the maternal level of 0.45 mmol/L), raising parental concern. They trialed quetiapine, which caused excessive sedation, and then switched to 2.5 mg/day aripiprazole. Aripiprazole led to a rapid drop in milk supply and mild hypomanic symptoms. After 2 wks, she discontinued aripiprazole and resumed Li^+^, which restored milk production within 48 h. The infant’s Li^+^ level later dropped to 0.20 mmol/L, and breastfeeding continued successfully.
Morin & Chevalier, 2017 [62]	♀ (age not reported) Canadian, BD	Continued lamotrigine 250 mg/day, aripiprazole 15 mg/day, and sertraline 100 mg/day throughout pregnancy and postpartum. Exclusively breastfed.	Term ♂ infant, spontaneous vaginal delivery. At 12 days of life, presented with 30% weight loss and severe hypernatremic dehydration (Na^+^ 199 mEq/L). ICU admission: hypovolemic shock, renal failure, DIC, and right lower limb gangrene requiring amputation of all five toes. Lamotrigine plasma level: 13.0 µmol/L (3.33 µg/mL). No breast milk after admission. Mild gross motor delay at 5 and 13 months.
Yskes et al., 2018 [63]	♀ 40 yrs, African American, unspecified BD and GAD with panic attacks	Treated 5 wks postpartum with hydroxyzine 50 mg/day → aripiprazole 5 mg/day added. Was also taking milk thistle and fenugreek supplements. Did not take risperidone.	Decreased milk production reported after 5 days of aripiprazole + hydroxyzine, requiring formula supplementation. Milk production returned to normal within 9 days after stopping both medications. Aripiprazole was suspected as causal (Naranjo score = 2). No further psychotropics were used.
Ballester-Gracia et al., 2019 [64]	♀ 43 yrs, Spanish, BD	History of multiple manic relapses due to treatment discontinuation while trying to conceive. Switched from paliperidone LAI to aripiprazole LAI (400 mg/month), then reduced to 300 mg/month at patient’s request upon confirmed pregnancy (~2–3 weeks GA). Treatment maintained throughout pregnancy.	Delivered a healthy ♀ at 40 + 4 weeks via spontaneous vaginal delivery. Birth weight 3500 g, Apgar scores 9/10/10, umbilical cord pH 7.29. No congenital malformations or developmental issues at 5-month FU. Pregnancy and delivery proceeded without complications. Aripiprazole LAI resumed at 400 mg/month postpartum.
Walker et al., 2019 [65]	♀ 31 yrs, anxious-depressive, primipara	Pt treated before pregnancy with 2 mg/day aripiprazole and 225 mg/day venlafaxine. Aripiprazole discontinued at GW 6. Pt delivered a healthy baby (gender undisclosed) after cesarean section at 36.5 GW due to preeclampsia. She breastfed her infant, supplementing it with expressed breast milk and formula to overcome reduced milk production. She developed postpartum depression at day 8 postpartum and resumed 2 mg/aripiprazole.	3 days after the reintroduction of low-dose aripiprazole, milk production declined. The infant was latching properly, but swinged between effective and noneffective nutrition patterns. By day 15, it was less than 30 mL/day. At day 21, milk production had ceased. The baby passed to a formula-milk feeding and thrived.
Solé et al., 2020 [66]	♀ 31 yrs, SCZ, Caucasian	Hospitalized different times and treated with risperidone, but developed hyperprolactinemia and amenorrhea. She switched to aripiprazole 20 mg/day and then to aripiprazole LAI 400 mg/4 wks. She became pregnant, continuing on aripiprazole. Prolactinemia was normal. She delivered at GW 41 a healthy ♀ infant.	Good symptom control, no adverse obstetric outcomes for mother and baby. No lactation or thriving outcomes reported. This case adds to the literature of safe antipsychotic use in pregnancy, but fails to report on lactation, a field in which literature is more cautious.
Komaroff, 2021 [67]	♀ 30 yrs, G1P1001, White (USA), bipolar depression and anxiety	Switched from sodium valproate and clonazepam to aripiprazole 10 mg/day and sertraline 50 mg/day during early pregnancy. Continued both medications throughout pregnancy and postpartum.	C-S at GW 41 due to non-reassuring tracing and nuchal cord. Infant lost 11.3% body weight postpartum, requiring formula. Mother experienced lactation failure: no engorgement, no expressed milk, low prolactin levels (7.4 → 7.7). Psychiatric medication not modified. Persistent difficulty with bonding, depressive symptoms postpartum (EPDS scores 12 → 13 → 11 → 7). No breastfeeding established.
Fernández-Abascal et al., 2021 [68]	**M1**: ♀ 39 yrs, Spanish, paranoid SCZ **M2**: ♀ 32 yrs, Spanish, SCZ and STPD **M3**: ♀ 36 yrs, Spanish, paranoid SCZ, cutaneous lupus, type I obesity **M4**: ♀ 31 yrs, Spanish, SCZ **M5**: ♀ 39 yrs, Black (Senegal), SCZ **M6**: ♀ 30 yrs, Spanish, SCZ (initially SCZF, SIPD)	**M1**: Aripiprazole LAI 400 mg/28 days × 32 months before conception. Continued during pregnancy with reduced dose to 300 mg/28 d. **M2**: Aripiprazole LAI 400 mg/28 days for 19 months before pregnancy. Continued in pregnancy, reduced to 300 mg/28 d. **M3**: Aripiprazole LAI 400 mg/28 days for 5 years. Reduced to 300 mg/28 d during pregnancy. **M4**: Aripiprazole LAI 160 mg/28 days before and during pregnancy. Continued unchanged. **M5**: Switched from paliperidone LAI to oral aripiprazole 15 mg/day, then LAI 300 mg/28 days. Continued. **M6**: Aripiprazole LAI 400 mg/28 d before pregnancy, discontinued at end of 1TM, restarted postpartum.	**M1**: Term delivery (38 + 5 wks) of healthy ♂ infant. Breastfed. Normal development at 3 yrs. **M2**: Preterm vaginal delivery (31 + 5 wks) of ♀ infant. 1 month incubator. Not breastfed. Normal development at 2 yrs. **M3**: Term delivery (40 wks) of healthy ♂. Artificial lactation. Anti-SSa/SSb+; normal development at 1 month. Dose increased postpartum. Stable. **M4**: Term delivery (39 + 5 wks) of healthy ♂. No breastfeeding. Normal development at 2 yrs. Continued LAI 160 mg. **M5**: Term delivery (39 wks) of healthy ♂. No breastfeeding. Normal development at 12 months. Maintained LAI 300 mg. **M6**: Term delivery (40 wks) of healthy ♂. Not breastfed. Normal development at 18 months. Restarted LAI 400 mg postpartum. Stable.
Sahoo et al., 2023 [69]	**M1**: ♀ 29 yrs, Turkish, ICD-10 SCZ **M2**: ♀ 24 yrs, Turkish, SCZ	**M1**: Aripiprazole 20 mg/day and escitalopram 10 mg/day before and throughout pregnancy. **M2**: Aripiprazole 10 mg/day for 2 years before conception; continued during pregnancy.	**M1**: Reduced fetal movements at 33 weeks; hospitalized, monitored, no abnormalities. Spontaneous vaginal delivery at 38 weeks. Infant: Undisclosed sex; 2.69 kg, Apgar 6. Lactation failure. At 16 months: no developmental issues. **M2**: Forceps delivery under general anesthesia at 38 wks due to agitation during labor. Infant: ♂, 2.7 kg, intubated, recovered in 3 days. Lactation failure. At 1 yr: healthy, normal milestones.
Konishi et al., 2024 [70]	♀ 30 yrs, Japanese, SCZ	Switched from aripiprazole 24 mg/day to brexpiprazole 1 mg/day at ~2–4 wks GW due to fatigue. Dose increased to 2 mg/day at 18 wks GW. Risperidone 2 mg BID and quetiapine 12.5 mg BID added. All drugs continued postpartum.	C-S at 41 + 3 wks. Neonate: 3730 g, Apgar 8/9, normal physical exam. Ingested only colostrum (12 times between 46–86.5 h). Finnegan score 1 (nasal obstruction) on day 1, no neonatal withdrawal symptoms. Breast milk RID: brexpiprazole 0.47–1.1%, quetiapine < 0.01%, risperidone 0.9–1.0%, paliperidone 0.9–1.0%. PLs declined postnatally. No adverse events.
Pinci et al., 2024 [71]	♀ 30 yrs, Italian (Caucasian), SCZ	Paliperidone palmitate LAI 350 mg (PP3M), last dose at GW 5. Switched at GW 9 to oral aripiprazole 15 mg/day. Also received lorazepam 2.5 mg/day.	Elective C-S at 39 wks. No obstetric complications. Infant: ♀; weight 3.39 kg; length 48 cm; head circumference 36 cm; Apgar 10/10; no congenital malformations, negative newborn screenings. No breastfeeding. Suspicion of plagiocephaly ruled out. Normal development at 4-month FU. Sedation in 1st month of aripiprazole resolved spontaneously.
Herold et al., 2024 [72]	♀ 23 yrs, Hungarian, SCZ	Cariprazine 3 mg/day maintenance treatment before and throughout pregnancy. Escitalopram discontinued at GW 8. Psychiatric monitoring continued. Venlafaxine 50 mg/day added postpartum.	Spontaneous vaginal delivery at 40 weeks. ♀ infant; 2700 g; Apgar 9/9. Not breastfed due to maternal medication. No neonatal complications. 2-yr FU: normal development and functioning, appropriate attachment. No adverse effects.

Abbreviations: 1TM, first trimester; 2TM, second trimester; 3TM, third trimester; BD, bipolar disorder; BID, bis in die, twice a day; C-S, Cesarean section; DHAri, dehydroaripiprazole; FU, follow-up; G1P1001, woman with one pregnancy, one delivery, no preterm, no miscarriage, one live birth; G2P0010, woman with two pregnancies (G2), no previous deliveries to 24 weeks or beyond (P0), no previous preterm deliveries (0), no previous full-term deliveries (0), and one previous live birth (10); G2P2, woman with two pregnancies and two deliveries; GAD, generalized anxiety disorder; GW(s), gestation week(s); ICU, intensive care unit; LAI, long-acting injectable; LC, liquid chromatography; Li^+^, lithium; M, mother; MS, mass spectrometry; PL(s), plasma level(s); pt(s), patient(s); qHS, every night at bedtime, quaque hora somni; SCZ, schizophrenia; SCZF, schizophreniform disorder; STPD, schizotypal personality disorder; wk(s), SIPD, substance-induced psychotic disorder; wk(s), week(s); yr(s), year(s); ×, for, per; →, then, followed, after, subsequently; ↑, increased, augmented, rise, potentiated, stronger; ↓, reduced, reduction, diminished, lower, weaker; ↔, unchanged, no significant variation; ♀, female, girl, woman; ♂, male, boy, man.

**Table 3:** Summary of clinical studies on pregnancy and fetal outcomes of patients receiving dopamine partial agonists during pregnancy and/or lactation in chronological order.

Study	Population	Design	Treatment	Outcomes	Conclusions
Maňáková & Hubičková, 2011 [73]	Pregnant ♀ who contacted CTIS between 2002–2009. The study included 43 women exposed to SSRIs, 37 to atypical APs and ADs (including aripiprazole), and 85 controls exposed to non-teratogenic substances. Most had depression, anxiety, or SCZ.	Prospective observational study. Data collected via phone or email during 1TM, with FU after delivery to assess pregnancy outcomes.	Exposure to SSRIs (mainly citalopram, escitalopram, sertraline) and atypical APs and ADs (risperidone 15.4%, mirtazapine 20.5%, venlafaxine 28.2%, trazodone 15.4%, aripiprazole 5.1%, ziprasidone 2.6%, olanzapine 7.6%, quetiapine 5.1%) Most ♀ were on polytherapy, especially in the SSRI group (86%). Exposure occurred mainly during 1TM, often continuing through pregnancy.	Main outcomes included birth defects, ETOP, SAB, gestational age, birth weight, and length. Malformation rates were within expected range across all groups. SSRI showed higher ETOP rates, atypical psychotropics, more SAB, and lower birth weight and length.	SSRI exposure during pregnancy was not linked to increased risk of major malformations. However, small sample size limited statistical power. ↑ETOP rates in the SSRI group and ↑ SAB and growth restriction in the atypical psychotropics group suggest possible associations.
Bellet et al., 2015 [74]	86 pregnant ♀ exposed to aripiprazole during embryogenesis (4–10 GW), compared to 172 matched controls with no exposure or exposure to non-teratogenic agents. Most were treated for SCZ, psychotic disorders or BD; $\bar{x}$ age 31.8 yrs.	Prospective multicenter cohort study using data from two French teratology/pharmacovigilance databases (2004–2011), comparing aripiprazole-exposed pregnancies to matched unexposed controls.	Aripiprazole exposure during embryogenesis (GW 4–10), with doses ranging from 5 to 30 mg/day ($\bar{x}$ 13.5 mg/day). 35% continued after 1TM; 21% treated throughout pregnancy. Frequent co-medications included ADs, BZDs, and APs.	No significant increase in major malformations (2.8% vs 1.2%, OR 2.30, 95% CI 0.32–16.7). Aripiprazole exposure was associated with higher rates of prematurity (16.4% vs. 7.1%, OR 2.57) and FGR (19.0% vs. 7.3%, OR 2.97). Two neonatal complications occurred with late pregnancy exposure.	Aripiprazole exposure during embryogenesis was not significantly associated with major malformations. However, ↑ risks of prematurity and FGR were observed. Small sample size and confounding factors (e.g., polytherapy), limit the strength of conclusions.
Montastruc et al., 2016 [75]	Reports from the WHO VigiBase^®^ database (1967–2014), including 1235 cases of congenital malformations associated with APs. Among these, 4 reports of anorectal disorders were linked to aripiprazole.	Case/non-case disproportionality analysis based on VigiBase, covering reports from 1967 to 2014. The study applied statistical methods to detect safety signals while minimizing competition bias from other drugs and EADs.	Treatment involved exposure to AP medications, including aripiprazole. Among the 11 reports of congenital anorectal malformations, 4 were associated with aripiprazole use during pregnancy. Specific dosage and timing of exposure not available in the spontaneous reports.	The analysis revealed a signal of disproportionate reporting for congenital gastrointestinal malformations, including palate, esophageal, and anorectal disorders. 4/11 anorectal malformation reports were linked to aripiprazole. No detailed clinical outcomes or FU data were available due to the nature of spontaneous reporting.	Potential safety signal identified for gastrointestinal congenital malformations associated with AP use, including aripiprazole. Causality cannot be established.
Park et al., 2017 [76]	1,522,247 pregnant ♀ enrolled in Medicaid (2001–2010) who delivered live-born infants. Cohort included 15,196 ♀ exposed to APs during pregnancy, with 0.4% exposed to aripiprazole. Most users diagnosed with BD, depression, or SCZ.	Retrospective cohort study using U.S. Medicaid Analytic eXtract data (2001–2010). Antipsychotic use during pregnancy was assessed through pharmacy dispensing records, with analyses of trends, discontinuation, switching, and patient characteristics.	AP use defined as filling at least one outpatient prescription during pregnancy. Aripiprazole was among the 6 most frequently used agents, with exposure peaking at 0.4% by 2010. Polytherapy was common: 65% also received ADs, 25% BDZs, and 22% mood stabilizers.	The study described patterns of antipsychotic use but did not assess pregnancy or neonatal outcomes. Key findings included a 3-fold increase in SGA use (especially aripiprazole and quetiapine) and high rates of discontinuation (50%) during pregnancy.	Use of SGAs, including aripiprazole, ↑ significantly among Medicaid-insured pregnant ♀ from 2001 to 2010. ↑ discontinuation rates and frequent polytherapy reflect safety concerns and clinical uncertainty.
Sakai et al., 2017 [77]	4355 pregnancy-related spontaneous reports from the JADER database (2004–2015). Among these, 85 reports involved aripiprazole as a suspected drug; 18 were linked to miscarriage.	Disproportionality analysis of pregnancy-related spontaneous reports from the JADER database (2004–2015), comparing miscarriage reports linked to aripiprazole vs. other SGAs.	Aripiprazole was the suspected drug in 85 pregnancy-related reports. Among 18 miscarriage cases, the median dose was 12 mg (range 3–30 mg). Most cases listed SCZ as the diagnosis. Not consistently available specific timing of exposure and co-medications.	A safety signal for miscarriage was detected for aripiprazole (reporting odds ratio 2.76, 95% CI 1.62–4.69). No signal found for risperidone, olanzapine, or quetiapine. No information on congenital anomalies or live birth outcomes.	Potential safety signal for miscarriage associated with aripiprazole, not seen with other SGAs. Limitations of spontaneous reporting and lack of clinical details ↓ the strength of observations.
Westin et al., 2018 [78]	103 ♀ (110 pregnancies) from Norway, treated with APs and monitored through routine TDM. Dataset included 201 serum concentration measurements during pregnancy and 512 baseline/postpartum samples.	Retrospective observational study based on therapeutic drug monitoring data from two Norwegian hospitals (1999–2011). Included ♀ had serum AP levels measured during and outside pregnancy, allowing within-subject comparison of drug disposition across gestation.	Included oral treatment with nine APs, notably aripiprazole (15 mg/day, n = 14 pregnancies). Serum concentrations of aripiprazole and its active metabolite DHAri were analyzed. Median post-dose sampling time was 16.8 h during pregnancy.	Aripiprazole serum concentrations ↓ significantly during pregnancy—by approximately 52% in 3TM compared to baseline (*p* < 0.001). The parent/metabolite ratio also ↓, pointing to altered drug metabolism. Clinical outcomes (e.g., efficacy, safety) were not assessed.	Pregnancy significantly ↓ serum concentrations of aripiprazole, likely due to ↑ metabolic clearance. This pharmacokinetic change may lead to subtherapeutic exposure, supporting the need for clinical monitoring and possible dose adjustment during pregnancy.
Park et al., 2018 [79]	1,543,334 pregnancies from U.S. Medicaid data (2000–2010), all involving ♀ prescribed aripiprazole, olanzapine, quetiapine, risperidone, or ziprasidone before pregnancy. Among these, 1924 ♀ had prior exposure to aripiprazole and no preexisting diabetes.	Retrospective cohort study using U.S. Medicaid claims data (2000–2010). Compared women who continued vs. discontinued atypical AP treatment during early pregnancy, using propensity score stratification to adjust for confounders.	♀ filled prescriptions for aripiprazole, ziprasidone, quetiapine, risperidone, or olanzapine in the 3 months preceding pregnancy. Continuation was defined as ≥2 dispensings during the first 140 days of pregnancy. Aripiprazole continuers (n = 419) were compared to discontinuers (n = 1505).	The primary outcome was gestational diabetes, defined using diagnostic codes and glucose testing criteria. Among aripiprazole users, gestational diabetes occurred in 4.8% of continuers vs. 4.5% of discontinuers. Adjusted RR for aripiprazole was 0.82 (95% CI: 0.50–1.33), indicating no increased risk.	Continuing aripiprazole during early pregnancy not associated with ↑ risk of gestational diabetes vs. discontinuation. Favorable metabolic safety profile for aripiprazole in pregnant ♀.
Galbally et al., 2018 [80]	26 ♀ on aripiprazole during pregnancy, $\bar{x}$ age 28.92 ± 5.94 yrs; of them: 12 continued ($\bar{x}$ age 29.42 ± 7.10 yrs) and 14 discontinued ($\bar{x}$ age 28.50 ± 4.74 yrs).	Retrospective study based on hospital medical records of two sites (Mercy Hospital for Women, Heidelberg, Victoria, Australia and King Edward Memorial Hospital for Women, Subiaco, Western Australia); compared pregnancy and birth outcomes between continuers and discontinuers.	Aripiprazole during any trimester; $\bar{x}$ dose 17.98 ± 12.00 mg; range 5–60 mg in discontinuers; $\bar{x}$ dose 19.77 ± 15.99 mg; range 5–60 mg in continuers. 8 ♀ took aripiprazole in monotherapy.	Continuers vs. discontinuers did not differ for gestational diabetes mellitus (1, 1), pregnancy-induced hypertension (1, 3), antepartum hemorrhage (0, 2), C-S rate (5, 6), neonatal ICU admission of newborn (4, 6), GWs at delivery, infant birth weight, infant birth length, and infant head circumference. Only one infant of a continuer had an Apgar score of <7 at 5 min.	Aripiprazole had no significant effect on pregnancy and infant outcomes. No adverse metabolic outcomes, but gestational hypertension should be further explored. Very small sample.
Freeman et al., 2021 [81]	1906 ♀ aged 18–45 yrs enrolled at MGH from November 2008 to April 2020, of whom 158 were exposed to aripiprazole in 1TM ($\bar{x}$ age 32.4 ± 5.49 yrs) vs. 621 ♀ exposed to other APs in 1TM ($\bar{x}$ age 32.6 ± 5.14 yrs) vs. 690 ♀ unexposed ($\bar{x}$ age 32.7 ± 4.19 yrs).	Retrospective study based on NPRAA data. Patients on 1TM aripiprazole were compared to patients exposed to SGAs at 1TM and to unexposed ♀; the outcome was the occurrence of major malformations in the fetus/newborn.	Aripiprazole, doses not specified; co-administered drugs in 1TM: SGAs 22 ♀ (13.9%), FGAs 4 ♀ (2.5%), SSRIs 54 ♀ (34.2%), SNRIs 20 ♀ (12.7%) TCAs 4 ♀ (2.5%), lithium 6 ♀ (3.8%), anticonvulsants 49 ♀ (31.0%), antianxiety 28 ♀ (17.7%), sedatives 9 ♀ (5.7%), stimulants 18 ♀ (11.4%).	Unadjusted ORs for major malformations 2.212 (95%CI from 0.878 to 5.571), adjusted 1.35 for aripiprazole vs. unexposed (95%CI from 0.43 to 4.20), but after adjustment for confounders, the risk of major malformations following 1TM exposure to aripiprazole was not significant compared to unexposed. 7 major malformations in the aripiprazole group and 14 in the unexposed.	Although data are reassuring for major malformations, the samples are small and need to be amplified to yield reliable results.
Straub et al., 2022 [82]	2,034,883 unexposed pregnancies and 9551 pregnancies with ≥1 AP ($\bar{x}$ age 26.8 ± 6.1 yrs for MAX, $\bar{x}$ age 33.1 ± 5.0 yrs for MarketScan). Exposed to aripiprazole, 1,5016 ♀ MAX and 241 ♀ MarketScan.	Birth cohort study with data from MAX (2000–2014) and MarketScan (2003–2015) for insured mother–child dyads with up to 14 yrs FU. Outcomes of interest: development of any of the following: ASD, ADHD, learning difficulty, intellectual disability, developmental coordination disorder, behavioral disorder, and any specific neurodevelopmental disorder.	More ♀ exposed in 1TM than late pregnancy to aripiprazole showed adverse developmental outcomes (Any neurodevelopmental disorder, ASD, ADHD, learning difficulty, speech/language disorder, intellectual disability, and behavioral disorder). Most women exposed to aripiprazole were receiving the drug as their only AP.	Slightly ↑ odds for any neurodevelopmental disorder in the aripiprazole group only compared to other Aps.	It cannot be ruled out that the slightly unfavorable results found for aripiprazole are due to chance.
Jiang et al., 2024 [83]	FAERS September 2015-April 2023 brexpiprazole AE reports at any stage, not only pregnancy, not only ♀ (5129 ♀ [59.23%], 2312 ♂ [27.01%], 1118 undetermined).	Reports of AEs to the FARS database for brexpiprazole as the suspected drug.	Brexpiprazole at unspecified doses.	33 case reports for AEs regarding pregnancy, puerperium and perinatal conditions. 6 case reports of lactation disorder.	Brexpiprazole may not be safe for pregnancy and may be detrimental to lactation, which is not present in the drug’s instructions. This study did not specifically assess the teratogenic potential of brexpiprazole during pregnancy and lactation.
Ishikawa et al., 2024 [84]	44,118 miscarrying patients ($\bar{x}$ age 33.3 ± 5.7 yrs) were matched 1:3 with 132,317 controls-live births ($\bar{x}$ age 33.2 ± 5.5 yrs) (AP use 0.5% in both groups).	Nested case-control study based on data from the administrative claims database of JMDC Inc. (Tokyo, Japan). Miscarriage cases reported during 2013 to 2022; miscarriage cases considered those ending pregnancy between 4–22 GWs.	Various antipsychotics, classified as DPA (aripiprazole oral and LAI, and brexpiprazole), SDA (blonanserin, risperidone, paliperidone, lurasidone, and perospirone), and MARTA (asenapine, quetiapine, clozapine, and olanzapine), and BDZs.	Atypical AP use did not differ between miscarrying ♀ and those delivering live birth. This held true for DPA (oral and LAI), SDA and MARTA. Only BDZs had a higher ratio in miscarrying vs. control ♀ (n = 848 (1.9%) vs. 1791 (1.4%)). Limiting to the SCZ population, sensitivity analysis showed no association between AP use and miscarriage. Adjusted OR for all APs 0.966 (95%CI 0.796 to 1.173) and for aripiprazole 0.998 (95%CI 0.784 to 1.269).	No association between exposure to atypical APs during pregnancy and the risk of miscarriage.
Zheng et al., 2024 [85]	21,605 reports involving neonates, with 6208 cases reporting congenital anomalies; of these, 53.58% were ♂, 36.50% were ♀, and 9.92% undisclosed gender.	Reports of congenital malformations to the FARS database for any psychotropic as the suspected drug (99% attribution) from January 2004 to June 2023 up to 28 days of life.	Atypical APs (quetiapine, olanzapine, and aripiprazole), SSRIs (sertraline, paroxetine, and fluoxetine), SNRIs (venlafaxine and duloxetine) and NaSSA (mirtazapine).	Top ten psychiatric drugs associated with congenital abnormalities in newborns were venlafaxine, quetiapine, olanzapine, sertraline, citalopram, mirtazapine, duloxetine, paroxetine, aripiprazole, and fluoxetine; all psychotropics examined have an ↑ risk for congenital malformations, with aripiprazole and fluoxetine having the least risk.	Caution needed when administering psychotropics in pregnancy; aripiprazole among APs carries the least risk for congenital malformations.
Cho et al., 2025 [86]	11,406 reports of AP exposure during pregnancy.	WHO pharmacovigilance database 1968–2023; disproportionality analysis to calculate reporting ORs for adverse pregnancy, fetal, or neonatal outcomes associated with various APs in comparison to quetiapine.	Haloperidol, ziprasidone, clozapine, olanzapine, risperidone, paliperidone, and aripiprazole vs. quetiapine (no dose specifications).	Haloperidol vs. quetiapine ↑reporting frequency for congenital malformations (OR 3.83; 95%CI from 2.62 to 5.59); no differences for all other APs vs. quetiapine for congenital malformations and neonatal outcomes; ↑ abortion/stillbirth rate for haloperidol, clozapine, olanzapine, risperidone, and aripiprazole vs. quetiapine; ↑ delivery and postpartum complications for aripiprazole.	Some concerns for adverse neonatal outcomes for aripiprazole vs. quetiapine; ziprasidone data inconclusive due to few cases exposed. Design focused too much on quetiapine.
Nanjundaswamy et al., 2025 [87]	60 ♀ of 398 attending perinatal psychiatry services at NIMHNS-I were prescribed aripiprazole during pregnancy ($\bar{x}$ age 29 ± 4.4 yrs); 21 (60%) with SCZ, 19 (54.2%) primiparous; 35 continued aripiprazole during lactation ($\bar{x}$ illness duration 5 yrs).	NIMHNS-I data of perinatal psychiatric services 2016–2021. Lactation evaluated in ♀ who continued on aripiprazole during lactation. Lactation failure defined as total absence of milk flow or secretion of just a few drops of milk following suckling for × ≥7 days.	Aripiprazole $\bar{x}$ dose 16.4 mg/day ±7.4 mg × 20 ±8.5 months. 27 ♀ exposed to aripiprazole throughout pregnancy (22 infants delivered at term, 10 preterm).	26 of the 35 ♀ (74%) showed complete lactation failure; 4 (11%) had insufficient milk production. 27 ♀ exposed to aripiprazole throughout pregnancy, although 5 had already experienced lactation failure in previous pregnancies. 11 ♀ had lactation issues attributable to neonatal health concerns. 8 ♀ were able to continue aripiprazole and start lactation. 7 ♀ discontinued aripiprazole and switched to risperidone or olanzapine, achieving lactation anew.	Detrimental effect of aripiprazole on lactation confirmed (85% of the sample).

Abbreviations: 1TM, first trimester; 2TM, second trimester; 3TM, third trimester; 95%CI(s), ninety-five percent confidence interval(s); AD(s), antidepressant(s); ADHD, attention deficit/hyperactivity disorder; AE(s), adverse event(s); AP(s), antipsychotic(s); ASD, autism spectrum disorder; BD, bipolar disorder; BDZ(s), benzodiazepine(s); BID, bis in die, twice a day; C-S, Cesarean section; CTIS, Czech Teratology Information Service; DHAri, dehydroaripiprazole; DPA, dopamine partial agonists; EADs, emerging adverse events; ETOP, elective terminations; FAERS, FDA Adverse Event Reporting System; FGA(s), first-generation antipsychotic(s); FGR, fetal growth retardation FU, follow-up; G*N*P*ŃŇǸṄ*, woman with *N* pregnancy(ies), *Ń* delivery(ies), *Ň* preterm, *Ǹ* miscarriage(s), *Ṅ* live birth(s); GAD, generalized anxiety disorder; GW(s), gestation week(s); ICU, intensive care unit; JADER, Japanese Adverse Drug Event Report; LAI, long-acting injectable; LC, Liquid chromatography; LfA, large for age; MARTA, multi-acting receptor targeted antipsychotics; MAX, Medicaid Analytic eXtract; MarketScan, IBM Health MarketScan Research Database; MGH, Massachusetts General Hospital; NaSSA, noradrenaline-serotonin specific antagonists; NIMHNS-I, National Institute of Mental Health and Neuro Science of India; NPRAA, Massachusetts General Hospital National Pregnancy Registry for Atypical Antipsychotics; pt(s), patient(s); OR, odds-ratio; PL(s), plasma level(s); qHS, every night at bedtime, quaque hora somni; RR, relative risk; SAB, spontaneous abortions; SCZ, schizophrenia; SCZF, schizophreniform disorder; SDA, serotonin/dopamine antagonists; SfA, small for age; SGA(s), second-generation antipsychotic(s); SNRI(s), Serotonin-Norepinephrine Reuptake Inhibitor(s); SSRI(s), Selective Serotonin Reuptake Inhibitor(s); TDM, therapeutic drug monitoring; WHO, World Health Organization; wk(s), week(s); $\bar{x}$, mean; yr(s), year(s); ×, for, per; →, then, followed, after, subsequently; ↑, increased, augmented, rise, potentiated, stronger; ↓, reduced, reduction, diminished, lower, weaker; ↔, unchanged, no significant variation; ♀, female, girl, woman; ♂, male, boy, man.

## Data Availability

The review used published data; hence, no new data were created. All data are contained in the manuscript/Supplements and are accessible to all.

## Supplementary Materials

The following supporting information can be downloaded at: https://www.mdpi.com/article/10.3390/ph18071010/s1, Table S1: Search strategy and eligibility, with reasons for exclusion; Table S2: PRISMA 2020 Checklist. Table S3. Quality assessment of included studies.

## Abbreviations

The following abbreviations are used in this manuscript:

DAPAs	Dopamine D_2_/D_3_ receptor partial agonists
FGAs	First-generation antipsychotic drugs
LfA	Large for age
SfA	Small for age
SGAs	Second-generation antipsychotic drugs
