# Supplementary material for: Dopamine Partial Agonists in Pregnancy and Lactation: A Systematic Review
Source: Pharmaceuticals (Basel). 2025 Jul 6;18(7):1010. doi: 10.3390/ph18071010 (PMC12299336; doi:10.3390/ph18071010)
Supplement: Supplementary file 1 [file pharmaceuticals-18-01010-s001.zip › Koukopoulos et al 2025 Partial DA agonists in pregnancy_Pharmaceuticals_Suppl_Rev.pdf]

Supplementary Table S1. Search strategy and eligibility, with reasons for exclusion.
(cariprazine OR aripiprazole OR brexpiprazole OR "dopamine partial agonist*") AND (pregnant OR pregnancy OR lactation OR breastfeeding OR peripartum OR postpartum OR perinatal) 23.6.2025 PubMed → 152 results

1	Krause W, Hümpel M. Pharmacokinetics of the dopamine partial agonist, terguride, in the rat and rhesus monkey. Eur J Drug Metab Pharmacokinet. 1988;13(3):185-94. doi: 10.1007/BF03189938.	Animal
2	Gentile S. Clinical utilization of atypical antipsychotics in pregnancy and lactation. Ann Pharmacother. 2004;38(7-8):1265-71. doi: 10.1345/aph.1D485. Epub 2004 May 18.	Review
3	Li M, Budin R, Fleming AS, Kapur S. Effects of novel antipsychotics, amisulpiride and aripiprazole, on maternal behavior in rats. Psychopharmacology (Berl). 2005;181(3):600-10. doi: 10.1007/s00213-005-0091-7. Epub 2005 Oct 12.	Animal
4	Iqbal MM, Aneja A, Rahman A, Megna J, Freemont W, Shiplo M, Nihilani N, Lee K. The potential risks of commonly prescribed antipsychotics: during pregnancy and lactation. Psychiatry (Edgmont). 2005;2(8):36-44.	Review
**5**	**Mendhekar DN, Sharma JB, Srilakshmi P. Use of aripiprazole during late pregnancy in a woman with psychotic illness. Ann Pharmacother. 2006;40(3):575. doi: 10.1345/aph.1G507. Epub 2006 Feb 7.**	**Case**
6	Haefliger T, Bonsack C. Antipsychotiques atypiques et dysfonction sexuelle: à propos de cinq cas associés à la rispéridone [Atypical antipsychotics and sexual dysfunction: five case-reports associated with risperidone]. Encéphale. 2006;32(1 Pt 1):97-105. French. doi: 10.1016/s0013-7006(06)76142-5.	No preg/Lact
7	Gentile S. Prophylactic treatment of bipolar disorder in pregnancy and breastfeeding: focus on emerging mood stabilizers. Bipolar Disord. 2006;8(3):207-20. doi: 10.1111/j.1399-5618.2006.00295.x.	Review
**8**	**Mendhekar DN, Sunder KR, Andrade C. Aripiprazole use in a pregnant schizoaffective woman. Bipolar Disord. 2006;8(3):299-300. doi: 10.1111/j.1399-5618.2006.00316.x.**	**Case**
9	Aichhorn W, Whitworth AB, Weiss EM, Marksteiner J. Second-generation antipsychotics: is there evidence for sex differences in pharmacokinetic and adverse effect profiles? Drug Saf. 2006;29(7):587-98. doi: 10.2165/00002018-200629070-00004.	Unfocused
10	Donohoe DR, Weeks K, Aamodt EJ, Dwyer DS. Antipsychotic drugs alter neuronal development including ALM neuroblast migration and PLM axonal outgrowth in *Caenorhabditis elegans*. Int J Dev Neurosci. 2008;26(3-4):371-80. doi: 10.1016/j.ijdevneu.2007.08.021. Epub 2008 Jan 20.	Animal
**11**	**Mervak B, Collins J, Valenstein M. Case report of aripiprazole usage during pregnancy. Arch Womens Ment Health. 2008;11(3):249-50. doi: 10.1007/s00737-008-0022-9.**	**Case**
12	de Leon J, Greenlee B, Barber J, Sabaawi M, Singh NN. Practical guidelines for the use of new generation antipsychotic drugs (except clozapine) in adult individuals with intellectual disabilities. Res Dev Disabil. 2009;30(4):613-69. doi: 10.1016/j.ridd.2008.10.010. Epub 2008 Dec 11.	Unfocused
13	Einarson A, Boskovic R. Use and safety of antipsychotic drugs during pregnancy. J Psychiatr Pract. 2009;15(3):183-92. doi: 10.1097/01.pra.0000351878.45260.94.	Review
14	McCauley-Elsom K, Gurvich C, Elsom SJ, Kulkarni J. Antipsychotics in pregnancy. J Psychiatr Ment Health Nurs. 2010;17(2):97-104. doi: 10.1111/j.1365-2850.2009.01481.x.	Review
**15**	**Lutz UC, Hiemke C, Wiatr G, Farger G, Arand J, Wildgruber D. Aripiprazole in pregnancy and lactation: a case report. J Clin Psychopharmacol. 2010;30(2):204-5. doi: 10.1097/JCP.0b013e3181d27c7d.**	**Case**
**16**	**Nguyen T, Teoh S, Hackett LP, Ilett K. Placental transfer of aripiprazole. Aust N Z J Psychiatry. 2011;45(6):500-1. doi: 10.3109/00048674.2011.566547. Epub 2011 Mar 17.**	**Case**
17	Guillemot J, Lukaszewski MA, Montel V, Delahaye F, Mayeur S, Laborie C, Dickes-Coopman A, Dutriez-Casteloot I, Lesage J, Breton C, Vieau D. Influence of prenatal undernutrition on the effects of clozapine and aripiprazole in the adult male rats: relevance to a neurodevelopmental origin of schizophrenia? Eur J Pharmacol. 2011;667(1-3):402-9. doi: 10.1016/j.ejphar.2011.04.011. Epub 2011 Apr 15.	Animal
**18**	**Watanabe N, Kasahara M, Sugibayashi R, Nakamura T, Nakajima K, Watanabe O, Murashima A. Perinatal use of aripiprazole: a case report. J Clin Psychopharmacol. 2011;31(3):377-9. doi: 10.1097/JCP.0b013e318218c400.**	**Case**
19	Guillemot J, Laborie C, Dutriez-Casteloot I, Maron M, Deloof S, Lesage J, Breton C, Vieau D. Could maternal perinatal atypical antipsychotic treatments program later metabolic diseases in the offspring? Eur J Pharmacol. 2011;667(1-3):13-6. doi: 10.1016/j.ejphar.2011.05.076. Epub 2011 Jun 12.	Review
20	Bharadwaj B, Kattimani S, Mukherjee A. Aripiprazole for acute mania in an elderly person. Ind Psychiatry J. 2011;20(2):142-4. doi: 10.4103/0972-6748.102532.	No preg/Lact
**21**	**Gentile S, Tofani S, Bellantuono C. Aripiprazole and pregnancy: a case report and literature review. J Clin Psychopharmacol. 2011;31(4):531-2. doi: 10.1097/JCP.0b013e318222bc65.**	**Case**
**22**	**Maňáková E, Hubičková L. Antidepressant drug exposure during pregnancy. CZTIS small prospective study. Neuro Endocrinol Lett. 2011;32(Suppl 1):53-6.**	**Database**
23	Richtand NM, Ahlbrand R, Horn P, Tambyraja R, Grainger M, Bronson SL, McNamara RK. Fluoxetine and aripiprazole treatment following prenatal immune activation exert longstanding effects on rat locomotor response. Physiol Behav. 2012;106(2):171-7. doi: 10.1016/j.physbeh.2012.02.004. Epub 2012 Feb 9.	Animal
**24**	**Widschwendter CG, Hofer A. Aripiprazole use in early pregnancy: a case report. Pharmacopsychiatry. 2012;45(7):299-300. doi: 10.1055/s-0032-1312591. Epub 2012 May 30.**	**Case**
25	Potter PO, John N, Coffey DB. Onset of abnormal movements and cardiovascular symptoms after acute change in complex polypharmacy in a child with attention- deficit/hyperactivity disorder and mood symptoms. J Child Adolesc Psychopharmacol. 2012;22(5):388-92. doi: 10.1089/cap.2012.2253.	No preg/Lact
26	Ratajczak P, Kus K, Jarmuszkiewicz Z, Woźniak A, Nowakowska E. The effect of ethyl alcohol on the function of spatial memory in rats. Arzneimittelforschung. 2012;62(12):614-23. doi: 10.1055/s-0032-1327701. Epub 2012 Oct 31.	Animal
**27**	**Wakil L, Perea E, Penaskovic K, Stuebe A, Meltzer-Brody S. Exacerbation of psychotic disorder during pregnancy in the context of medication discontinuation. Psychosomatics. 2013;54(3):290-3. doi: 10.1016/j.psym.2012.07.003. Epub 2012 Dec 4.**	**Case**
28	Ratajczak P, Kus K, Jarmuszkiewicz Z, Woźniak A, Cichocki M, Nowakowska E. Influence of aripiprazole and olanzapine on behavioral dysfunctions of adolescent rats exposed to stress in perinatal period. Pharmacol Rep. 2013;65(1):30-43. doi: 10.1016/s1734-1140(13)70961-7.	Animal
29	Klinger G, Stahl B, Fusar-Poli P, Merlob P. Antipsychotic drugs and breastfeeding. Pediatr Endocrinol Rev. 2013;10(3):308-17.	Review
30	Besnard I, Auclair V, Callery G, Gabriel-Bordenave C, Roberge C. Hyperprolactinémies induites par les antipsychotiques : physiopathologie, clinique et surveillance [Antipsychotic-drug-induced hyperprolactinemia: physiopathology, clinical features and guidance]. Encéphale. 2014;40(1):86-94. French. doi: 10.1016/j.encep.2012.03.002. Epub 2013 Aug 5.	Review
31	Hasnain M, Vieweg WV. Weight considerations in psychotropic drug prescribing and switching. Postgrad Med. 2013;125(5):117-29. doi: 10.3810/pgm.2013.09.2706.	No preg/Lact
32	Singh KP, Tripathi N. Prenatal exposure of a novel antipsychotic aripiprazole: impact on maternal, fetal and postnatal body weight modulation in rats. Curr Drug Saf. 2014;9(1):43-8. doi: 10.2174/15748863113086660061.	Animal
33	Wade RL, Kindermann SL, Hou Q, Thase ME. Comparative assessment of adherence measures and resource use in SSRI/SNRI-treated patients with depression using second-generation antipsychotics or L-methylfolate as adjunctive therapy. J Manag Care Pharm. 2014;20(1):76-85. doi: 10.18553/jmcp.2014.20.1.76.	No preg/Lact
34	Nowakowska E, Kus K, Ratajczak P, Cichocki M, Woźniak A. The influence of aripiprazole, olanzapine and enriched environment on depressant-like behavior, spatial memory dysfunction and hippocampal level of BDNF in prenatally stressed rats. Pharmacol Rep. 2014;66(3):404-11. doi: 10.1016/j.pharep.2013.12.008. Epub 2014 Apr 3.	Animal
35	Lozano R, Marin R, Santacruz MJ. Prolactin deficiency by aripiprazole. J Clin Psychopharmacol. 2014;34(4):539-40. doi: 10.1097/JCP.0000000000000151.	No preg/Lact
**36**	**Windhager E, Kim SW, Saria A, Zauner K, Amminger PG, Klier CM. Perinatal use of aripiprazole: plasma levels, placental transfer, and child outcome in 3 new cases. J Clin Psychopharmacol. 2014;34(5):637-41. doi: 10.1097/JCP.0000000000000171.**	**Case**
37	Ratajczak P, Nowakowska E, Kus K, Danielewicz R, Herman S, Woźniak A. Neuroleptics and enrichment environment treatment in memory disorders and other central nervous system function observed in prenatally stressed rats. Hum Exp Toxicol. 2015;34(5):526-37. doi: 10.1177/0960327114543934. Epub 2014 Jul 25.	Animal
38	Gentile S. A safety evaluation of aripiprazole for treating schizophrenia during pregnancy and puerperium. Expert Opin Drug Saf. 2014;13(12):1733-42. doi: 10.1517/14740338.2014.951325. Epub 2014 Aug 19.	Review
39	Sharma V, Sommerdyk C, Xie B. Aripiprazole augmentation of antidepressants for postpartum depression: a preliminary report. Arch Womens Ment Health. 2015;18(1):131-4. doi: 10.1007/s00737-014-0462-3. Epub 2014 Sep 17.	No preg/Lact
**40**	**Frew JR. Psychopharmacology of bipolar I disorder during lactation: a case report of the use of lithium and aripiprazole in a nursing mother. Arch Womens Ment Health. 2015;18(1):135-6. doi: 10.1007/s00737-014-0469-9. Epub 2014 Oct 29.**	**Case**
41	Parikh T, Goyal D, Scarff JR, Lippmann S. Antipsychotic drugs and safety concerns for breast-feeding infants. South Med J. 2014;107(11):686-8. doi: 10.14423/SMJ.0000000000000190.	Review

**42**	**Pirec V, Mehta A, Shoush S. Aripiprazole combined with other psychotropic drugs in pregnancy: two case reports. Isr J Psychiatry Relat Sci. 2014;51(2):135-6.**	**Case**
43	Ennis ZN, Damkier P. Pregnancy exposure to olanzapine, quetiapine, risperidone, aripiprazole and risk of congenital malformations. A systematic review. Basic Clin Pharmacol Toxicol. 2015;116(4):315-20. doi: 10.1111/bcpt.12372. Epub 2015 Jan 28.	Review
**44**	**Bellet F, Beyens MN, Bernard N, Beghin D, Elefant E, Vial T. Exposure to aripiprazole during embryogenesis: a prospective multicenter cohort study. Pharmacoepidemiol Drug Saf. 2015;24(4):368-80. doi: 10.1002/pds.3749. Epub 2015 Feb 12.**	**Prospective**
45	Eaton WW, Chen LY, Dohan FC Jr, Kelly DL, Cascella N. Improvement in psychotic symptoms after a gluten-free diet in a boy with complex autoimmune illness. Am J Psychiatry. 2015;172(3):219-21. doi: 10.1176/appi.ajp.2014.14040550.	Unrelated
46	Bellantuono C, Di Massimo G, Mauro A, Martellini M, Nardi B. Aripiprazolo in gravidanza: una rassegna della letteratura internazionale [Aripiprazole in pregnancy: a review of literature]. Riv Psichiatr. 2015;50(1):8-11. Italian. doi: 10.1708/1794.19526.	Review
47	Hu LY, Lee YT, Lu T, Hung MB, Hung YY. Using aripiprazole to treat new-onset hyperprolactinemia-related delusion of pregnancy. Aust N Z J Psychiatry. 2015;49(10):946. doi: 10.1177/0004867415589796. Epub 2015 Jun 22.	No preg/Lact
48	Leiderman EA, Lorenzo L. Hábitos prescriptivos en el tratamiento de la esquizofrenia [PRESCRIPTION PATTERNS IN THE TREATMENT OF SCHIZOPHRENIA]. Vertex. 2015;26(119):11-6. Spanish.	Unfocused
49	Leiderman EA, Lorenzo L. Hábitos prescriptivos en el tratamiento de la esquizofrenia [Prescription patterns in the treatment of schizophrenia]. Vertex. 2015;XXVI(119):11-16. Spanish.	Dupl. 48PM
50	Ratajczak P, Kus K, Giermaziak W, Nowakowska E. The influence of aripiprazole and olanzapine on the anxiolytic-like effect observed in prenatally stressed rats (animal model of schizophrenia) exposed to the ethyl alcohol. Pharmacol Rep. 2016;68(2):415-22. doi: 10.1016/j.pharep.2015.10.010. Epub 2015 Nov 10.	Animal
**51**	**Montastruc F, Salvo F, Arnaud M, Bégaud B, Pariente A. Signal of Gastrointestinal Congenital Malformations with Antipsychotics After Minimising Competition Bias: A Disproportionality Analysis Using Data from Vigibase(®). Drug Saf. 2016;39(7):689-96. doi: 10.1007/s40264-016-0413-1.**	**Database**
52	Uguz F. Second-Generation Antipsychotics During the Lactation Period: A Comparative Systematic Review on Infant Safety. J Clin Psychopharmacol. 2016;36(3):244-52. doi: 10.1097/JCP.0000000000000491.	Review
53	Ratajczak P, Kus K, Gołembiowska K, Noworyta-Sokołowska K, Woźniak A, Zaprutko T, Nowakowska E. The influence of aripiprazole and olanzapine on neurotransmitters level in frontal cortex of prenatally stressed rats. Environ Toxicol Pharmacol. 2016;46:122-130. doi: 10.1016/j.etap.2016.07.007. Epub 2016 Jul 18.	Animal
54	An FR, Yang R, Wang ZM, Ungvari GS, Ng CH, Chiu HF, Wu PP, Jin X, Li L, Lok GK, Xiang YT. Hyperprolactinemia, prolactin-related side effects and quality of life in Chinese psychiatric patients. Compr Psychiatry. 2016;71:71-76. doi: 10.1016/j.comppsych.2016.08.009. Epub 2016 Aug 24.	No preg/Lact
55	Korade Ž, Liu W, Warren EB, Armstrong K, Porter NA, Konradi C. Effect of psychotropic drug treatment on sterol metabolism. Schizophr Res. 2017;187:74-81. doi: 10.1016/j.schres.2017.02.001. Epub 2017 Feb 12.	Unfocused
56	Kus K, Ratajczak P, Czaja N, Zaprutko T, Nowakowska E. Effect of combined administration of aripiprazole and fluoxetine on cognitive functions in female rats exposed to ethyl alcohol. Acta Neurobiol Exp (Wars). 2017;77(1):86-93. doi: 10.21307/ane-2017-039.	Animal
57	Kaneta H, Ukai W, Tsujino H, Furuse K, Kigawa Y, Tayama M, Ishii T, Hashimoto E, Kawanishi C. Antipsychotics promote GABAergic interneuron genesis in the adult rat brain: Role of heat-shock protein production. J Psychiatr Res. 2017;92:108-118. doi: 10.1016/j.jpsychires.2017.03.008. Epub 2017 Mar 10.	Animal
**58**	**Morin C, Chevalier I. Severe Hypernatremic Dehydration and Lower Limb Gangrene in an Infant Exposed to Lamotrigine, Aripiprazole, and Sertraline in Breast Milk. Breastfeed Med. 2017;12(6):377-380. doi: 10.1089/bfm.2017.0031. Epub 2017 May 8.**	**Case**
**59**	**Park Y, Huybrechts KF, Cohen JM, Bateman BT, Desai RJ, Patorno E, Mogun H, Cohen LS, Hernandez-Diaz S. Antipsychotic Medication Use Among Publicly Insured Pregnant Women in the United States. Psychiatr Serv. 2017;68(11):1112-1119. doi: 10.1176/appi.ps.201600408. Epub 2017 Jun 15.**	**Database**
**60**	**Westin AA, Brekke M, Molden E, Skogvoll E, Castberg I, Spigset O. Treatment With Antipsychotics in Pregnancy: Changes in Drug Disposition. Clin Pharmacol Ther. 2018;103(3):477-484. doi: 10.1002/cpt.770. Epub 2017 Sep 19.**	**Longitudinal**
**61**	**Sakai T, Ohtsu F, Mori C, Tanabe K, Goto N. Signal of Miscarriage with Aripiprazole: A Disproportionality Analysis of the Japanese Adverse Drug Event Report Database. Drug Saf. 2017;40(11):1141-1146. doi: 10.1007/s40264-017-0560-z.**	**Database**
62	Gentile S. Safety concerns associated with second-generation antipsychotic long-acting injection treatment. A systematic update. Horm Mol Biol Clin Investig. 2017;36(2):20170004./j/hmbci.2018.36.issue-2/hmbci-2017-0004/hmbci-2017-0004.xml. doi: 10.1515/hmbci-2017-0004.	Review
63	Amerio A, Tonna M, Odone A, Ghaemi SN. The Osler's view: Treating comorbid bipolar disorder and obsessive-compulsive disorder. Aust N Z J Psychiatry. 2017;51(9):944-945. doi: 10.1177/0004867417721653. Epub 2017 Jul 27.	No preg/Lact
64	Hara Y, Ago Y, Taruta A, Hasebe S, Kawase H, Tanabe W, Tsukada S, Nakazawa T, Hashimoto H, Matsuda T, Takuma K. Risperidone and aripiprazole alleviate prenatal valproic acid-induced abnormalities in behaviors and dendritic spine density in mice. Psychopharmacology (Berl). 2017;234(21):3217-3228. doi: 10.1007/s00213-017-4703-9. Epub 2017 Aug 10.	Animal
65	Drazanova E, Ruda-Kucerova J, Kratka L, Horska K, Demlova R, Starcuk Z Jr, Kasparek T. Poly(I:C) model of schizophrenia in rats induces sex-dependent functional brain changes detected by MRI that are not reversed by aripiprazole treatment. Brain Res Bull. 2018;137:146-155. doi: 10.1016/j.brainresbull.2017.11.008. Epub 2017 Nov 16.	Animal
66	Ratajczak P, Kus K, Skurzyńska M, Nowakowska E. The influence of aripiprazole and venlafaxine on the antidepressant-like effect observed in prenatally stressed rats (animal model of depression). Hum Exp Toxicol. 2018;37(9):972-982. doi: 10.1177/0960327117747023. Epub 2017 Dec 14.	Animal
67	Drobnis EZ, Nangia AK. Psychotropics and Male Reproduction. Adv Exp Med Biol. 2017;1034:63-101. doi: 10.1007/978-3-319-69535-8_8.	No preg/Lact
68	Cuomo A, Goracci A, Fagiolini A. Aripiprazole use during pregnancy, peripartum and lactation. A systematic literature search and review to inform clinical practice. J Affect Disord. 2018;228:229-237. doi: 10.1016/j.jad.2017.12.021. Epub 2017 Dec 14.	Review
69	Damkier P, Videbech P. The Safety of Second-Generation Antipsychotics During Pregnancy: A Clinically Focused Review. CNS Drugs. 2018;32(4):351-366. doi: 10.1007/s40263-018-0517-5.	Review
70	Genaro-Mattos TC, Tallman KA, Allen LB, Anderson A, Mirnics K, Korade Z, Porter NA. Dichlorophenyl piperazines, including a recently-approved atypical antipsychotic, are potent inhibitors of DHCR7, the last enzyme in cholesterol biosynthesis. Toxicol Appl Pharmacol. 2018;349:21-28. doi: 10.1016/j.taap.2018.04.029. Epub 2018 Apr 23.	Unfocused
**71**	**Park Y, Hernandez-Diaz S, Bateman BT, Cohen JM, Desai RJ, Patorno E, Glynn RJ, Cohen LS, Mogun H, Huybrechts KF. Continuation of Atypical Antipsychotic Medication During Early Pregnancy and the Risk of Gestational Diabetes. Am J Psychiatry. 2018;175(6):564-574. doi: 10.1176/appi.ajp.2018.17040393. Epub 2018 May 7.**	**Database**
**72**	**Galbally M, Frayne J, Watson SJ, Snellen M. Aripiprazole and pregnancy: A retrospective, multicentre study. J Affect Disord. 2018;238:593-596. doi: 10.1016/j.jad.2018.06.004. Epub 2018 Jun 14.**	**Small sample**
**73**	**Yskes R, Thomas R, Nagalla ML. A Case of Decreased Milk Production Associated With Aripiprazole. Prim Care Companion CNS Disord. 2018;20(6):18l02303. doi: 10.4088/PCC.18l02303.**	**Case**
74	Uguz F. A New Safety Scoring System for the Use of Psychotropic Drugs During Lactation. Am J Ther. 2021;28(1):e118-e126. doi: 10.1097/MJT.0000000000000909.	Unfocused
75	Uguz F. Antipsychotic Use During Pregnancy and the Risk of Gestational Diabetes Mellitus: A Systematic Review. J Clin Psychopharmacol. 2019;39(2):162-167. doi: 10.1097/JCP.0000000000001002.	Review
76	Genaro-Mattos TC, Allen LB, Anderson A, Tallman KA, Porter NA, Korade Z, Mirnics K. Maternal aripiprazole exposure interacts with 7-dehydrocholesterol reductase mutations and alters embryonic neurodevelopment. Mol Psychiatry. 2019;24(4):491-500. doi: 10.1038/s41380-019-0368-6. Epub 2019 Feb 11.	Animal
77	Takahashi K, Nakagawasai O, Sakuma W, Nemoto W, Odaira T, Lin JR, Onogi H, Srivastava LK, Tan-No K. Prenatal treatment with methylazoxymethanol acetate as a neurodevelopmental disruption model of schizophrenia in mice. Neuropharmacology. 2019;150:1-14. doi: 10.1016/j.neuropharm.2019.02.034. Epub 2019 Mar 1.	Animal
78	Cuomo A, Beccarini Crescenzi B, Goracci A, Bolognesi S, Giordano N, Rossi R, Facchi E, Neal SM, Fagiolini A. Drug safety evaluation of aripiprazole in bipolar disorder. Expert Opin Drug Saf. 2019;18(6):455-463. doi: 10.1080/14740338.2019.1617847. Epub 2019 May 17.	Review
79	Lian J, Deng C. Early antipsychotic exposure affects NMDA and GABAA receptor binding in the brains of juvenile rats. Psychiatry Res. 2019;273:739-745. doi: 10.1016/j.psychres.2019.02.001. Epub 2019 Feb 2.	Animal
**80**	**Ballester-Gracia I, Pérez-Almarcha M, Galvez-Llompart A, Hernandez-Viadel M. Use of long acting injectable aripiprazole before and through pregnancy in bipolar disorder: a case report. BMC Pharmacol Toxicol. 2019;20(1):52. doi: 10.1186/s40360-019-0330-x.**	**Case**
81	Dragioti E, Solmi M, Favaro A, Fusar-Poli P, Dazzan P, Thompson T, Stubbs B, Firth J, Fornaro M, Tsartsalis D, Carvalho AF, Vieta E, McGuire P, Young AH, Shin JI, Correll CU, Evangelou E. Association of Antidepressant Use With Adverse Health Outcomes: A Systematic Umbrella Review. JAMA Psychiatry. 2019;76(12):1241-1255. doi: 10.1001/jamapsychiatry.2019.2859. Erratum in: JAMA Psychiatry. 2021;78(5):569. doi: 10.1001/jamapsychiatry.2021.0314.	Review

82	Anmella G, Pacchiarotti I, Cubała WJ, Dudek D, Maina G, Thomas P, Vieta E. Expert advice on the management of valproate in women with bipolar disorder at childbearing age. Eur Neuropsychopharmacol. 2019;29(11):1199-1212. doi: 10.1016/j.euroneuro.2019.09.007. Epub 2019 Oct 4.	Review
83	Beaulieu AM, Tabasky E, Osser DN. The psychopharmacology algorithm project at the Harvard South Shore Program: An algorithm for adults with obsessive-compulsive disorder. Psychiatry Res. 2019;281:112583. doi: 10.1016/j.psychres.2019.112583. Epub 2019 Sep 27.	Unfocused
84	Hara Y (原 雄大). ドパミン神経系賦活化薬の慢性投与は自閉症モデルマウスの行動異常を改善する[Chronic Activation of the Dopaminergic Neuronal Pathway Improves Behavioral Abnormalities in the Prenatal Valproic Acid Exposure Mouse Model of Autism Spectrum Disorder]. Yakugaku Zasshi. 2019;139(11):1391-1396. Japanese. doi: 10.1248/yakushi.19-00131.	Animal
85	Zhuo C, Xun Z, Hou W, Ji F, Lin X, Tian H, Zheng W, Chen M, Liu C, Wang W, Chen C. Surprising Anticancer Activities of Psychiatric Medications: Old Drugs Offer New Hope for Patients With Brain Cancer. Front Pharmacol. 2019;10:1262. doi: 10.3389/fphar.2019.01262.	Unfocused
86	Ratajczak P, Kus K, Murawiecka P, Słodzińska I, Zaprutko T, Kopciuch D, Paczkowska A, Nowakowska E. Memory deterioration based on the tobacco smoke exposure and methylazoxymethanol acetate administration vs. aripiprazole, olanzapine and enrichment environment conditions. Pharmacol Biochem Behav. 2020;189:172855. doi: 10.1016/j.pbb.2020.172855. Epub 2020 Jan 15.	Animal
87	Drugs for Depression. Med Lett Drugs Ther. 2020;62(1592):25-32.	Opinion
88	Genaro-Mattos TC, Anderson A, Allen LB, Tallman KA, Porter NA, Korade Z, Mirnics K. Maternal cariprazine exposure inhibits embryonic and postnatal brain cholesterol biosynthesis. Mol Psychiatry. 2020;25(11):2685-2694. doi: 10.1038/s41380-020-0801-x. Epub 2020 Jun 5.	Animal
89	Uguz F. Pharmacological prevention of mood episodes in women with bipolar disorder during the perinatal period: A systematic review of current literature. Asian J Psychiatr. 2020;52:102145. doi: 10.1016/j.ajp.2020.102145. Epub 2020 May 12.	Review
90	Lumateperone (Caplyta) for schizophrenia. Med Lett Drugs Ther. 2020;62(1603):113-116.	Opinion
91	Zheng L, Tang S, Tang R, Xu M, Jiang X, Wang L. Dose Adjustment of Quetiapine and Aripiprazole for Pregnant Women Using Physiologically Based Pharmacokinetic Modeling and Simulation. Clin Pharmacokinet. 2021;60(5):623-635. doi: 10.1007/s40262-020-00962-3. Epub 2020 Nov 30.	No Data
92	Tallman KA, Allen LB, Klingelsmith KB, Anderson A, Genaro-Mattos TC, Mirnics K, Porter NA, Korade Z. Prescription Medications Alter Neuronal and Glial Cholesterol Synthesis. ACS Chem Neurosci. 2021;12(4):735-745. doi: 10.1021/acschemneuro.0c00765. Epub 2021 Feb 2.	Animal
**93**	**Freeman MP, Viguera AC, Góez-Mogollón L, Young AV, Caplin PS, McElheny SA, Church TR, Chitayat D, Hernández-Díaz S, Cohen LS. Reproductive safety of aripiprazole: data from the Massachusetts General Hospital National Pregnancy Registry for Atypical Antipsychotics. Arch Womens Ment Health. 2021;24(4):659-667. doi: 10.1007/s00737-021-01115-6. Epub 2021 Mar 12. Erratum in: Arch Womens Ment Health. 2021;24(4):669-670. doi: 10.1007/s00737-021-01133-4.**	**Database**
**94**	**Komaroff A. Aripiprazole and lactation failure: The importance of shared decision making. A case report. Case Rep Womens Health. 2021;30:e00308. doi: 10.1016/j.crwh.2021.e00308.**	**Case**
95	Korade Z, Heffer M, Mirnics K. Medication effects on developmental sterol biosynthesis. Mol Psychiatry. 2022;27(1):490-501. doi: 10.1038/s41380-021-01074-5. Epub 2021 Apr 5.	Review
96	Freeman MP, Viguera AC, Góez-Mogollón L, Young AV, Caplin PS, McElheny SA, Church TR, Chitayat D, Hernández-Díaz S, Cohen LS. Correction to: Reproductive safety of aripiprazole: data from the Massachusetts General Hospital National Pregnancy Registry for Atypical Antipsychotics. Arch Womens Ment Health. 2021;24(4):669-670. doi: 10.1007/s00737-021-01133-4. Erratum for: Arch Womens Ment Health. 2021;24(4):659-667. doi: 10.1007/s00737-021-01115-6.	Dupl. 93PM
97	Tasaki M, Yasui-Furukori N, Yokoyama S, Shinozaki M, Sugawara N, Shimoda K. Hypoprolactinemia and hyperprolactinemia in male schizophrenia patients treated with aripiprazole and risperidone and their relationships with testosterone levels. Neuropsychopharmacol Rep. 2021;41(3):379-384. doi: 10.1002/npr2.12190. Epub 2021 Jun 29.	No preg/Lact
98	Román V, Adham N, Foley AG, Hanratty L, Farkas B, Lendvai B, Kiss B. Cariprazine alleviates core behavioral deficits in the prenatal valproic acid exposure model of autism spectrum disorder. Psychopharmacology (Berl). 2021;238(9):2381-2392. doi: 10.1007/s00213-021-05851-6. Epub 2021 Jul 15.	Animal
99	Medved S, Bajs Janović M, Štimac Z, Mihaljević-Peleš A. Add-on Oxytocin in the Treatment of Postpartum Acute Schizophrenia: A Case Report. J Psychiatr Pract. 2021;27(4):326-332. doi: 10.1097/PRA.0000000000000557.	Unsuitable C
100	Andrade C. Major Congenital Malformations Associated With Exposure to Second-Generation Antipsychotic Drugs During Pregnancy. J Clin Psychiatry. 2021;82(5):21f14252. doi: 10.4088/JCP.21f14252.	Review
101	Liang CS, Su TP, Hsieh MH, Lee CS, Kuo J, Chiu NY, Chen PS, Yen YC, Bai YM. Taiwan Expert Consensus Recommendations for Switching to Aripiprazole Long-Acting Once-Monthly in Patients with Schizophrenia. J Pers Med. 2021;11(11):1198. doi: 10.3390/jpm11111198.	Opinion
102	Wichniak A, Siwek M, Rymaszewska J, Janas-Kozik M, Wolańczyk T, Bieńkowski P, Dudek D, Heitzman J, Szulc A, Samochowiec J. The position statement of the Working Group of the Polish Psychiatric Association on the use of D2/D3 dopamine receptor partial agonists in special populations. Psychiatr Pol. 2021;55(5):967-987. English, Polish. doi: 10.12740/PP/140287. Epub 2021 Oct 31.	Opinion
**103**	**Fernández-Abascal B, Recio-Barbero M, Sáenz-Herrero M, Segarra R. Long-acting injectable aripiprazole in pregnant women with schizophrenia: a case-series report. Ther Adv Psychopharmacol. 2021;11:2045125321991277. doi: 10.1177/2045125321991277.**	**Case**
104	Rogóż Z, Kamińska K, Lech MA, Lorenc-Koci E. N-Acetylcysteine and Aripiprazole Improve Social Behavior and Cognition and Modulate Brain BDNF Levels in a Rat Model of Schizophrenia. Int J Mol Sci. 2022;23(4):2125. doi: 10.3390/ijms23042125.	Animal
**105**	**Straub L, Hernández-Díaz S, Bateman BT, Wisner KL, Gray KJ, Pennell PB, Lester B, McDougle CJ, Suarez EA, Zhu Y, Zakoul H, Mogun H, Huybrechts KF. Association of Antipsychotic Drug Exposure in Pregnancy With Risk of Neurodevelopmental Disorders: A National Birth Cohort Study. JAMA Intern Med. 2022;182(5):522-533. doi: 10.1001/jamainternmed.2022.0375.**	**Database**
106	Rizwan M, Shahid NUA, Naguit N, Jakkoju R, Laeeq S, Reghefaoui T, Zahoor H, Yook JH, Mohammed L. Efficacy of Behavioural Intervention, Antipsychotics, and Alpha Agonists in the Treatment of Tics Disorder in Tourette's Syndrome. Cureus. 2022;14(2):e22449. doi: 10.7759/cureus.22449.	No preg/Lact
107	Yoon J, Gu J, Martin KB. A Novel Treatment of Postpartum Depression and Review of Literature. Cureus. 2022;14(2):e22373. doi: 10.7759/cureus.22373.	No preg/Lact
108	Farias R, Hartnett J. Perinatal Catatonia in a Patient with a Twin Pregnancy of Unknown Chorionicity and Gestational Age Presenting in Spontaneous Preterm Labor. Case Rep Obstet Gynecol. 2022;2022:3143601. doi: 10.1155/2022/3143601.	Unfocused
109	Llorca PM, Nuss P, Fakra É, Alamome I, Drapier D, El Hage W, Jardri R, Mouchabac S, Rabbani M, Simon N, Vacheron MN, Azorin JM. Place of the partial dopamine receptor agonist aripiprazole in the management of schizophrenia in adults: a Delphi consensus study. BMC Psychiatry. 2022;22(1):364. doi: 10.1186/s12888-022-04008-9.	Review
110	Andrade C. Attention-Deficit/Hyperactivity Disorder, Autism Spectrum Disorder, and Other Neurodevelopmental Outcomes Associated With Antipsychotic Drug Exposure During Pregnancy. J Clin Psychiatry. 2022;83(3):22f14529. doi: 10.4088/JCP.22f14529.	Review
111	Lumateperone (Caplyta) for bipolar depression. Med Lett Drugs Ther. 2022;64(1656):126-128.	Opinion
112	Andrade C. Psychotropic Drugs With Long Half-Lives: Implications for Drug Discontinuation, Occasional Missed Doses, Dosing Interval, and Pregnancy Planning. J Clin Psychiatry. 2022;83(4):22f14593. doi: 10.4088/JCP.22f14593.	Review
113	Lian J, Han M, Su Y, Hodgson J, Deng C. The long-lasting effects of early antipsychotic exposure during juvenile period on adult behaviours - A study in a poly I:C rat model. Pharmacol Biochem Behav. 2022;219:173453. doi: 10.1016/j.pbb.2022.173453. Epub 2022 Aug 25.	Animal
114	Zheng L, Yang H, Dallmann A. Antidepressants and Antipsychotics in Human Pregnancy: Transfer Across the Placenta and Opportunities for Modeling Studies. J Clin Pharmacol. 2022;62(Suppl 1):S115-S128. doi: 10.1002/jcph.2108.	Review
115	Samalin L, Arnould A, Boudieu L, Henry C, Haffen E, Drapier D, Anmella G, Pacchiarotti I, Vieta E, Belzeaux R, Llorca PM. Avis d'experts français sur la prise en charge des femmes en âge de procréer et enceintes souffrant d'un trouble bipolaire traitées par valproate [French Expert advice on the management of valproate in childbearing and pregnant women with bipolar disorder]. Encéphale. 2022;48(6):624-631. French. doi: 10.1016/j.encep.2022.07.005. Epub 2022 Oct 17.	Review
116	Naughton S, O'Hara K, Nelson J, Keightley P. Aripiprazole, brexpiprazole, and cariprazine can affect milk supply: Advice to breastfeeding mothers. Australas Psychiatry. 2023;31(2):201-204. doi: 10.1177/10398562231159510. Epub 2023 Feb 24.	Review
117	de Oliveira Ferreira E, Pessoa Gomes JM, Neves KRT, Lima FAV, de Barros Viana GS, de Andrade GM. Maternal treatment with aripiprazole prevents the development of a valproic acid-induced autism-like phenotype in juvenile male mice. Behav Pharmacol. 2023;34(2-3):154-168. doi: 10.1097/FBP.0000000000000718. Epub 2023 Feb 10.	Animal
118	Zohny SM, Habib MZ, Mohamad MI, Elayat WM, Elhossiny RM, El-Salam MFA, Hassan GAM, Aboul-Fotouh S. Memantine/Aripiprazole Combination Alleviates Cognitive Dysfunction in Valproic Acid Rat Model of Autism: Hippocampal CREB/BDNF Signaling and Glutamate Homeostasis. Neurotherapeutics. 2023;20(2):464-483. doi: 10.1007/s13311-023-01360-w. Epub 2023 Mar 14.	Animal
119	Koch MT, Carlson HE, Kazimi MM, Correll CU. Antipsychotic-Related Prolactin Levels and Sexual Dysfunction in Mentally Ill Youth: A 3-Month Cohort Study. J Am Acad Child Adolesc Psychiatry. 2023;62(9):1021-1050. doi: 10.1016/j.jaac.2023.03.007. Epub 2023 Mar 15.	No preg/Lact

120	Kumon H, Yoshino Y, Ozaki T, Funahashi Y, Mori H, Ueno M, Ozaki Y, Yamazaki K, Ochi S, Iga JI, Ueno SI. Gestational exposure to haloperidol changes Cdkn1a and Apaf1 mRNA expressions in mouse hippocampus. Brain Res Bull. 2023;199:110662. doi: 10.1016/j.brainresbull.2023.110662. Epub 2023 May 6.	Animal
121	Cariprazine (Vraylar) for adjunctive treatment of depression. Med Lett Drugs Ther. 2023;65(1677):84-86. doi: 10.58347/tml.2023.1677c.	Opinion
**122**	**Sahoo MK, Biswas H, Grover S. Safety Profile of Aripiprazole During Pregnancy and Lactation: Report of 2 Cases. Türk Psikiyatri Derg. 2023;34(2):133-135. doi: 10.5080/u26681.**	**Case**
123	Solmi M, De Toffol M, Kim JY, Choi MJ, Stubbs B, Thompson T, Firth J, Miola A, Croatto G, Baggio F, Michelon S, Ballan L, Gerdle B, Monaco F, Simonato P, Scocco P, Ricca V, Castellini G, Fornaro M, Murru A, Vieta E, Fusar-Poli P, Barbui C, Ioannidis JPA, Carvalho AF, Radua J, Correll CU, Cortese S, Murray RM, Castle D, Shin JI, Dragioti E. Balancing risks and benefits of cannabis use: umbrella review of meta-analyses of randomised controlled trials and observational studies. BMJ. 2023;382:e072348. doi: 10.1136/bmj-2022-072348.	No DA partial agonists
124	Mother To Baby | Fact Sheets [Internet]. Brentwood (TN): Organization of Teratology Information Specialists (OTIS); 1994–. Aripiprazole (Abilify®). 2023 Oct.	Opinion
**125**	**Jiang Y, Zhou L, Shen Y, Zhou Q, Ji Y, Zhu H. Safety assessment of Brexpiprazole: Real-world adverse event analysis from the FAERS database. J Affect Disord. 2024;346:223-229. doi: 10.1016/j.jad.2023.11.025. Epub 2023 Nov 11.**	**Database**
126	Drugs for depression. Med Lett Drugs Ther. 2023;65(1691):193-200. doi: 10.58347/tml.2023.1691a.	Opinion
127	Shimada Y, Oda Y, Shibata S, Hirose Y, Sasaki T. Successful Challenge With Brexpiprazole for Idiopathic Hypersomnia in a Patient With Bipolar Disorder: A Case Report. Cureus. 2024;16(1):e53182. doi: 10.7759/cureus.53182.	No preg/Lact
128	Chen KW, Schultz L, Hughes N. A Case of Postpartum Obsessive-Compulsive Disorder in a First-Time Father. Cureus. 2024;16(2):e54547. doi: 10.7759/cureus.54547.	No preg/Lact
129	Drugs for bipolar disorder. Med Lett Drugs Ther. 2024;66(1699):49-54. doi: 10.58347/tml.2024.1699a.	Opinion
**130**	**Konishi T, Kitahiro Y, Fujiwara N, Yamamoto K, Hashimoto M, Ito T, Itohara K, Fujioka K, Imafuku H, Otsuka I, Omura T, Yano I. Pharmacokinetics of Brexpiprazole, Quetiapine, Risperidone, and Its Active Metabolite Paliperidone in a Postpartum Woman and Her Baby. Ther Drug Monit. 2024;46(5):687-691. doi: 10.1097/FTD.0000000000001197. Epub 2024 Apr 4.**	**Case**
**131**	**Pinci C, Bianciardi E, Sferra I, Castellani G, Santini R, Siracusano A, Niolu C. Switching from paliperidone palmitate 3-monthly long-acting injection to oral aripiprazole in a pregnant woman with schizophrenia: a case report and short review. Riv Psichiatr. 2024;59(2):75-79. doi: 10.1708/4259.42361.**	**Case**
132	Othman MA, Husni M, El-Din WAN, Salem AH, Sarwani N, Rashid A, Fadel R. Prenatal aripiprazole induces alterations of rat placenta: a histological, immunohistochemical and ultrastructural study. J Mol Histol. 2024;55(4):415-426. doi: 10.1007/s10735-024-10199-0. Epub 2024 May 7.	Animal
133	Mother To Baby | Fact Sheets [Internet]. Brentwood (TN): Organization of Teratology Information Specialists (OTIS); 1994–. Cariprazine (Vraylar®). 2024 Jun.	Opinion
134	Gursoy BK, Atay E, Bilir A, Firat F, Soylemez ESA, Kurt GA, Gozen M, Ertekin T. Effect of aripiprazole on neural tube development in early chick embryos. Toxicol Appl Pharmacol. 2024;489:117009. doi: 10.1016/j.taap.2024.117009. Epub 2024 Jun 19.	Animal
**135**	**Herold R, Tényi T, Herold M, Tóth T. Cariprazine maintenance treatment during pregnancy – a case report. Front Psychiatry. 2024;15:1421395. doi: 10.3389/fpsyt.2024.1421395.**	**Case**
136	DeBattista C, Schatzberg AF. The Black Book of Psychotropic Dosing and Monitoring. Psychopharmacol Bull. 2024;54(3):8-59.	No preg/Lact
137	Iloperidone (Fanapt) - a new indication for bipolar disorder. Med Lett Drugs Ther. 2024;66(1707):115-116. doi: 10.58347/tml.2024.1707c.	Opinion
138	Zhu Y, Huang S, Chai D, Liang L. G protein-coupled receptor 1 participating in the mechanism of mediating gestational diabetes mellitus by phosphorylating the AKT pathway. Open Life Sci. 2024;19(1):20220920. doi: 10.1515/biol-2022-0920.	Animal
**139**	**Ishikawa T, Sakai T, Iwama N, Obara R, Morishita K, Adomi M, Noda A, Ishikuro M, Kikuchi S, Kobayashi N, Tomita H, Saito M, Nishigori H, Kuriyama S, Mano N, Obara T. Association between exposure to atypical antipsychotics during pregnancy and risk of miscarriage. Acta Psychiatr Scand. 2024;150(6):562-572. doi: 10.1111/acps.13755. Epub 2024 Sep 5.**	**Database**
140	Wang E, Liu Y, Wang Y, Han X, Zhou Y, Zhang L, Tang Y. Comparative Safety of Antipsychotic Medications and Mood Stabilizers During Pregnancy: A Systematic Review and Network Meta-analysis of Congenital Malformations and Prenatal Outcomes. CNS Drugs. 2025;39(1):1-22. doi: 10.1007/s40263-024-01131-x. Epub 2024 Nov 11.	Review
**141**	**Zheng J, Zhang Z, Liang Y, Wu Q, Din C, Wang Y, Ma L, Su L. Risk of congenital anomalies associated with psychotropic medications: a review of neonatal reports in the FDA adverse event reporting System (FAERS). Arch Womens Ment Health. 2024. doi: 10.1007/s00737-024-01540-3. Epub ahead of print Dec 23.**	**Database**
**142**	**Cho H, Jo H, Jeong YD, Jang W, Park J, Yim Y, Lee K, Lee H, Lee S, Fond G, Boyer L, Pizzol D, Jung J, Yon DK. Antipsychotic use during pregnancy and outcomes in pregnant individuals and newborns. J Affect Disord. 2025;373:495-504. doi: 10.1016/j.jad.2024.12.102. Epub 2025 Jan 2.**	**Database**
143	Teodorescu A, Dima L, Petric PS, Necula RM, Banciu R, Moga MA, Marian- Pavlenco A, Ifteni P. Treatment With Long-Acting Injectable Aripiprazole During Pregnancy in Bipolar Disorder: A Scoping Review. Am J Ther. 2024;31(6):e635-e644. doi: 10.1097/MJT.0000000000001773.	Review
144	Jeong Y, Son S, Park J, Kim CY, Kim J. Antidepressant aripiprazole induces adverse effects on neural development during cortex organoid generation. Reprod Toxicol. 2025;133:108862. doi: 10.1016/j.reprotox.2025.108862. Epub 2025 Feb 17.	*In Vitro*
145	Drugs and Lactation Database (LactMed®) [Internet]. Bethesda (MD): National Institute of Child Health and Human Development; 2006–. Brexpiprazole. 2025 Mar 15.	Review
146	Drugs and Lactation Database (LactMed®) [Internet]. Bethesda (MD): National Institute of Child Health and Human Development; 2006–. Cariprazine. 2025 Mar 15.	Review
147	Drugs and Lactation Database (LactMed®) [Internet]. Bethesda (MD): National Institute of Child Health and Human Development; 2006–. Aripiprazole. 2025 Mar 15.	Review
148	Wang H, Li JT, Liu DN, Zhang XQ, Sun M, Zhang CC, Si TM, Su YA. Environmental enrichment improves deficits in hippocampal neuroplasticity and cognition in prenatally aripiprazole-exposed mouse offspring. Transl Psychiatry. 2025;15(1):102. doi: 10.1038/s41398-025-03335-1.	Animal
**149**	**Nanjundaswamy MH, Shah A, Lotlikar S, Arasappa R, Ganjekar S, Thippeswamy H, Chandra PS, Desai G. Lactation-Related Side Effects of Aripiprazole: A Study From Perinatal Psychiatry Services in India. J Clin Psychopharmacol. 2025. doi: 10.1097/JCP.0000000000001997. Epub ahead of print Apr 9.**	**Prospective**
150	Bediako-Kakari P, Monyo M, Atoyebi S, Olagunju A. Comparative modelling of foetal exposure to maternal long-acting injectable versus oral daily antipsychotics. NPJ Womens Health. 2025;3(1):31. doi: 10.1038/s44294-025-00077-9. Epub 2025 May 15.	Unfocused
151	Jain A, Dhir N, Prabha PK, Raja A, Sharma AR, Kaundal T, Charan S, Bhatia A, Banerjee D, Saikia B, Zohmangaihi D, Goyal MK, Medhi B, Prakash A. Adenylyl cyclase activator: Forskolin mediates CREB Ser133 phosphorylation in the hippocampus, alleviates autism-like deficits in a valproic acid model of Wistar rats. J Neurosci Res. 2025;103(6):e70049. doi: 10.1002/jnr.70049.	Animal
152	Jain A, Dhir N, Prabha PK, Raja A, Sharma AR, Kaundal T, Charan S, Singh H, Singla R, Malik D, Bhatia A, Banerjee D, Saikia B, Zohmangaihi D, Goyal MK, Medhi B, Prakash A. Restoring Brain Function in Autism: GSK3β Inhibition by 6-Bromoindirubin-3'-oxime Reverses Valproic Acid-induced Neuropathology. ACS Chem Neurosci. 2025. doi: 10.1021/acschemneuro.5c00125. Epub ahead of print 2025 Jun 20.	Animal
(cariprazine OR aripiprazole OR brexpiprazole OR "dopamine partial agonist*") AND (pregnant OR pregnancy OR lactation OR breastfeeding OR peripartum OR postpartum OR perinatal) 23.6.2025 CINAHL → 58 results		
153	Mortimer AM. Aripiprazole is effective for relapse prevention in people with chronic stable schizophrenia. Evid Based Ment Health. 2004;7(2):41. doi: 10.1136/ebmh.7.2.41.	No preg/Lact
154	Haddad PM, Wieck A. Antipsychotic-induced hyperprolactinaemia: mechanisms, clinical features and management. Drugs. 2004;64(20):2291-314. doi: 10.2165/00003495-200464200-00003.	Review
155	Mendhekar DN, Sharma JB, Srilakshmi P. Use of aripiprazole during late pregnancy in a woman with psychotic illness. Ann Pharmacother. 2006;40(3):575. doi: 10.1345/aph.1G507. Epub 2006 Feb 7.	Dupl. 5PM
156	Chou JC-Y. Continuing aripiprazole after stabilisation of a manic or mixed episode of bipolar I disorder delays relapse. Evid Based Ment Health. 2007;10(1):13. doi: 10.1136/ebmh.10.1.13.	No preg/Lact
157	Taylor D. Haloperidol, olanzapine and risperidone reduce treatment failure compared to aripiprazole, quetiapine and ziprasidone in acute schizophrenia. Evid Based Ment Health. 2007;10(3):76. doi: 10.1136/ebmh.10.3.76.	No preg/Lact
158	Psychopharmacology reviews. August 2007. Primary Psychiatry	Opinion
159	New drugs. Drug news. P&T: A Peer-Reviewed Journal for Managed Care & Formulary Management, 2007;32	Opinion
160	A roadmap to key pharmacologic principles in using antipsychotics. Prim Care Companion J Clin Psychiatry. 2007;9(6):444-54.	Opinion
161	Psychiatric dispatches. Noteworthy briefs from the field. Primary Psychiatry, 2008	Opinion

162	Mervak B, Collins J, Valenstein M. Case report of aripiprazole usage during pregnancy. Arch Womens Ment Health. 2008;11(3):249-50. doi: 10.1007/s00737-008-0022-9.	Dupl. 11PM
163	Lambert TJ. Switching to aripiprazole from olanzapine leads to weight loss in overweight people with schizophrenia or schizoaffective disorder. Evid Based Ment Health. 2009;12(2):50. doi: 10.1136/ebmh.12.2.50.	No preg/Lact
164	Einarson A, Boskovic R. Use and safety of antipsychotic drugs during pregnancy. J Psychiatr Pract. 2009;15(3):183-92. doi: 10.1097/01.pra.0000351878.45260.94.	Dupl. 13PM
165	McCauley-Elsom K, Gurvich C, Elsom SJ, Kulkarni J. Antipsychotics in pregnancy. J Psychiatr Ment Health Nurs. 2010;17(2):97-104. doi: 10.1111/j.1365-2850.2009.01481.x.	Dupl. 14PM
166	Aripiprazole use during pregnancy and lactation examined. Brown University Psychopharmacology Update, 2010;21(6):8. doi:10.1002/pu.20117. Refers to: Lutz UC, Hiemke C, Wiatr G, Farger G, Arand J, Wildgruber D. Aripiprazole in pregnancy and lactation: a case report. J Clin Psychopharmacol. 2010;30(2):204-5. doi: 10.1097/JCP.0b013e3181d27c7d.	Dupl. 15PM
167	Nguyen T, Teoh S, Hackett LP, Ilett K. Placental transfer of aripiprazole. Aust N Z J Psychiatry. 2011;45(6):500-1. doi: 10.3109/00048674.2011.566547. Epub 2011 Mar 17.	Dupl. 16PM
168	Patient-oriented evidence that matters: POEMs. JAAPA: Journal of the American Academy of Physician Assistants (Haymarket Media, Inc.), 2011; 24(7):57-58.	Opinion
169	Digest. Progress in Neurology & Psychiatry, 2012;16(1):32-35.	Opinion
170	Advisor Forum. Clinical Advisor, 2012	Opinion
171	Shuster, Joel. ISMP Adverse Drug Reactions. Hosp Pharm. 2013;48(2):100–103	Opinion
172	Hasnain M, Vieweg WV. Weight considerations in psychotropic drug prescribing and switching. Postgrad Med. 2013;125(5):117-29. doi: 10.3810/pgm.2013.09.2706.	Dupl. 31PM
173	New from NICE. Prescriber, Sep2013. Opinion on a Review.	Opinion
174	Varela FA, Der-Ghazarian T, Lee RJ, Charntikov S, Crawford CA, McDougall SA. Repeated aripiprazole treatment causes dopamine D2 receptor up-regulation and dopamine supersensitivity in young rats. J Psychopharmacol. 2014;28(4):376-86. doi: 10.1177/0269881113504016. Epub 2013 Sep 17.	Animal
175	Pirec V, Mehta A, Shoush S. Aripiprazole combined with other psychotropic drugs in pregnancy: two case reports. Isr J Psychiatry Relat Sci. 2014;51(2):135-6.	Dupl. 42PM
176	Sharma V, Sommerdyk C, Xie B. Aripiprazole augmentation of antidepressants for postpartum depression: a preliminary report. Arch Womens Ment Health. 2015;18(1):131-4. doi: 10.1007/s00737-014-0462-3. Epub 2014 Sep 17.	Dupl. 39PM
177	Frew JR. Psychopharmacology of bipolar I disorder during lactation: a case report of the use of lithium and aripiprazole in a nursing mother. Arch Womens Ment Health. 2015;18(1):135-6. doi: 10.1007/s00737-014-0469-9. Epub 2014 Oct 29.	Dupl. 40PM
178	Grover S, Avasthi A. Mood stabilizers in pregnancy and lactation. Indian J Psychiatry. 2015;57(Suppl 2):S308-23. doi: 10.4103/0019-5545.161498.	Review
179	Hu LY, Lee YT, Lu T, Hung MB, Hung YY. Using aripiprazole to treat new-onset hyperprolactinemia-related delusion of pregnancy. Aust N Z J Psychiatry. 2015;49(10):946. doi: 10.1177/0004867415589796. Epub 2015 Jun 22.	Dupl. 47PM
180	Montastruc F, Salvo F, Arnaud M, Bégaud B, Pariente A. Signal of Gastrointestinal Congenital Malformations with Antipsychotics After Minimising Competition Bias: A Disproportionality Analysis Using Data from Vigibase(®). Drug Saf. 2016;39(7):689-96. doi: 10.1007/s40264-016-0413-1.	Dupl. 51PM
181	Keane AG, Bastiampillai T, Dhillon R, Tibrewal P, Kaler V. Delusion of pregnancy - Is there a role of prolactin? Aust N Z J Psychiatry. 2016;50(10):1018. doi: 10.1177/0004867416664591. Epub 2016 Aug 17.	No preg/Lact
182	An FR, Yang R, Wang ZM, Ungvari GS, Ng CH, Chiu HF, Wu PP, Jin X, Li L, Lok GK, Xiang YT. Hyperprolactinemia, prolactin-related side effects and quality of life in Chinese psychiatric patients. Compr Psychiatry. 2016;71:71-76. doi: 10.1016/j.comppsych.2016.08.009. Epub 2016 Aug 24.	Dupl. 54PM
183	Korade Ž, Liu W, Warren EB, Armstrong K, Porter NA, Konradi C. Effect of psychotropic drug treatment on sterol metabolism. Schizophr Res. 2017;187:74-81. doi: 10.1016/j.schres.2017.02.001. Epub 2017 Feb 12.	Dupl. 55PM
184	Sakai T, Ohtsu F, Mori C, Tanabe K, Goto N. Signal of Miscarriage with Aripiprazole: A Disproportionality Analysis of the Japanese Adverse Drug Event Report Database. Drug Saf. 2017;40(11):1141-1146. doi: 10.1007/s40264-017-0560-z.	Dupl. 61PM
185	Park Y, Huybrechts KF, Cohen JM, Bateman BT, Desai RJ, Patorno E, Mogun H, Cohen LS, Hernandez-Diaz S. Antipsychotic Medication Use Among Publicly Insured Pregnant Women in the United States. Psychiatr Serv. 2017;68(11):1112-1119. doi: 10.1176/appi.ps.201600408. Epub 2017 Jun 15.	Dupl. 59PM
186	Cuomo A, Goracci A, Fagiolini A. Aripiprazole use during pregnancy, peripartum and lactation. A systematic literature search and review to inform clinical practice. J Affect Disord. 2018;228:229-237. doi: 10.1016/j.jad.2017.12.021. Epub 2017 Dec 14.	Dupl. 68PM
187	Damkier P, Videbech P. The Safety of Second-Generation Antipsychotics During Pregnancy: A Clinically Focused Review. CNS Drugs. 2018;32(4):351-366. doi: 10.1007/s40263-018-0517-5.	Dupl. 69PM
188	Suzuki H, Hibino H, Inoue Y, et al. A patient with schizophrenia who lactated owing to mastopathy during aripiprazole once-monthly 300 mg treatment. International Medical Journal: IMJ 2018;25:90-1.	No preg/Lact
189	Park Y, Hernandez-Diaz S, Bateman BT, Cohen JM, Desai RJ, Patorno E, Glynn RJ, Cohen LS, Mogun H, Huybrechts KF. Continuation of Atypical Antipsychotic Medication During Early Pregnancy and the Risk of Gestational Diabetes. Am J Psychiatry. 2018;175(6):564-574. doi: 10.1176/appi.ajp.2018.17040393. Epub 2018 May 7.	Dupl. 71PM
190	Brown University Psychopharmacology Update, Diabetes risk higher among some women who stay on antipsychotic while pregnant. 2018;29(9):1,5. doi.org/10.1002/pu.30349. Opinion on 71PM	Opinion
191	Ratajczak P, Kus K, Skurzyńska M, Nowakowska E. The influence of aripiprazole and venlafaxine on the antidepressant-like effect observed in prenatally stressed rats (animal model of depression). Hum Exp Toxicol. 2018;37(9):972-982. doi: 10.1177/0960327117747023. Epub 2017 Dec 14.	Dupl. 66PM
192	Brown University Psychopharmacology Research Roundup. Update, 2018	Opinion
193	Galbally M, Frayne J, Watson SJ, Snellen M. Aripiprazole and pregnancy: A retrospective, multicentre study. J Affect Disord. 2018;238:593-596. doi: 10.1016/j.jad.2018.06.004. Epub 2018 Jun 14.	Dupl. 72PM
194	Pan B, Lian J, Deng C. Chronic antipsychotic treatment differentially modulates protein kinase A- and glycogen synthase kinase 3 beta-dependent signaling pathways, N-methyl-D-aspartate receptor and γ-aminobutyric acid A receptors in nucleus accumbens of juvenile rats. J Psychopharmacol. 2018;32(11):1252-1263. doi: 10.1177/0269881118788822. Epub 2018 Aug 23.	Animal
195	Mancano MA, Esordi MV, Patel DD, Milenki KJ. ISMP Adverse Drug Reactions: Longitudinal Thumbnail Fissures Due to Erlotinib Priapism Associated With the Use of ExtenZe Blindness From a Nevirapine-Based HAART Regimen Hyperprolactinemia and Galactorrhea Due to Aripiprazole Trypophobia Associated With Gabapentin Coadministered Linezolid and Methadone Cause Serotonin Syndrome. Hosp Pharm. 2019;54(2):88-92. doi: 10.1177/0018578718824449. Epub 2019 Jan 23.	Opinion
**196**	**Walker T, Coursey C, Duffus ALJ. Low Dose of Abilify (Aripiprazole) in Combination With Effexor XR (Venlafaxine HCl) Resulted in Cessation of Lactation: A Case Report. Clinical Lactation. 2019;10(2):56-59. doi: 10.1891/2158-0782.10.2.56.**	**Case**
197	Luo T, Liu QS, Yang YJ, Wei B. Aripiprazole for the treatment of duloxetine-induced hyperprolactinemia: A case report. J Affect Disord. 2019;250:330-332. doi: 10.1016/j.jad.2019.03.006. Epub 2019 Mar 5.	No preg/Lact
**198**	**Solé E, Duran JI, Lera S, Torres A. Aripiprazole long-acting injectable in schizophrenia during pregnancy: a case report. Schizophr Bull. 2020;46(Suppl.1):S117. S206. DOI:10.1093/schbul/sbaa031.272.**	**Case**
199	Uguz F. A New Safety Scoring System for the Use of Psychotropic Drugs During Lactation. Am J Ther. 2021;28(1):e118-e126. doi: 10.1097/MJT.0000000000000909.	Dupl. 74PM
200	Zheng L, Tang S, Tang R, Xu M, Jiang X, Wang L. Dose Adjustment of Quetiapine and Aripiprazole for Pregnant Women Using Physiologically Based Pharmacokinetic Modeling and Simulation. Clin Pharmacokinet. 2021;60(5):623-635. doi: 10.1007/s40262-020-00962-3. Epub 2020 Nov 30.	Dupl. 91PM
201	Medved S, Bajs Janović M, Štimac Z, Mihaljević-Peleš A. Add-on Oxytocin in the Treatment of Postpartum Acute Schizophrenia: A Case Report. J Psychiatr Pract. 2021;27(4):326-332. doi: 10.1097/PRA.0000000000000557.	Dupl. 99PM
202	Freeman MP, Viguera AC, Góez-Mogollón L, Young AV, Caplin PS, McElheny SA, Church TR, Chitayat D, Hernández-Díaz S, Cohen LS. Reproductive safety of aripiprazole: data from the Massachusetts General Hospital National Pregnancy Registry for Atypical Antipsychotics. Arch Womens Ment Health. 2021;24(4):659-667. doi: 10.1007/s00737-021-01115-6. Epub 2021 Mar 12. Erratum in: Arch Womens Ment Health. 2021;24(4):669-670. doi: 10.1007/s00737-021-01133-4.	Dupl. 93PM
203	Freeman MP, Viguera AC, Góez-Mogollón L, Young AV, Caplin PS, McElheny SA, Church TR, Chitayat D, Hernández-Díaz S, Cohen LS. Reproductive safety of aripiprazole: data from the Massachusetts General Hospital National Pregnancy Registry for Atypical Antipsychotics. Arch Womens Ment Health. 2021;24(4):659-667. doi: 10.1007/s00737-021-01115-6. Epub 2021 Mar 12. Erratum in: Arch Womens Ment Health. 2021;24(4):669-670. doi: 10.1007/s00737-021-01133-4.	Dupl. 93PM

204	Román V, Adham N, Foley AG, Hanratty L, Farkas B, Lendvai B, Kiss B. Cariprazine alleviates core behavioral deficits in the prenatal valproic acid exposure model of autism spectrum disorder. Psychopharmacology (Berl). 2021;238(9):2381-2392. doi: 10.1007/s00213-021-05851-6. Epub 2021 Jul 15.	Dupl. 98PM
205	Edinoff AN, Nix CA, Fort JM, Kimble J, Guedry R, Thomas G, Cornett EM, Kaye AD. Sexual Dysfunction in Schizophrenia: A Narrative Review of the Mechanisms and Clinical Considerations. Psychiatry Int. 2022;3(1):29-42. doi:10.3390/psychiatryint3010003.	Review
206	Zheng L, Yang H, Dallmann A. Antidepressants and Antipsychotics in Human Pregnancy: Transfer Across the Placenta and Opportunities for Modeling Studies. J Clin Pharmacol. 2022;62(Suppl 1):S115-S128. doi: 10.1002/jcph.2108.	Dupl. 114PM
207	Sharma M, Shankar P, Kukreti P, Kataria D. A rare case report of pseudo-pregnancy in a menopausal female. Indian J Psychiatry. 2023;65(7):789-792. doi: 10.4103/indianjpsychiatry.indianjpsychiatry_430_22. Epub 2023 Jul 12.	No preg/Lact
208	Komaroff, Ariana R. Advocacy and Lactation Support for Patient With Bipolar Disorder: A Case Report. Clinical Lactation. 2023, DOI: 10.1891/CL-2023-0006. Duplicate case of 94PM	Dupl. 94PM
209	Teodorescu A, Dima L, Petric PS, Necula RM, Banciu R, Moga MA, Marian- Pavlenco A, Ifteni P. Treatment With Long-Acting Injectable Aripiprazole During Pregnancy in Bipolar Disorder: A Scoping Review. Am J Ther. 2024;31(6):e635-e644. doi: 10.1097/MJT.0000000000001773.	Dupl 143PM
210	Aksoy, Begüm Gökhan; Oruç, Evrim Bayrak; Nazlı, Şerif Bora. A Rare Case: Mania Following Corticosteroid Withdrawal. Poster Presentation. Pub No: P-003. urkish Neuropsychiatric Society, 1st Mood Disorders Congress, March 27-30, 2024, Izmir, Turkey. Arch Neuropsychiatry. 2024;61:(Ek 1): 1-54. doi:10.29399/npa.28860.	Unsuitable C
(cariprazine OR aripiprazole OR brexpiprazole OR "dopamine partial agonist*") AND (pregnant OR pregnancy OR lactation OR breastfeeding OR peripartum OR postpartum OR perinatal) 23.6.2025 PsycINFO/PsycARTICLES → 94 results		
211	Hughes, Deborah;Naccari, Christopher. Noteworthy briefs from the field. Primary Psychiatry, Sep 2003	Unrelated
212	Iqbal MM, Aneja A, Rahman A, Megna J, Freemont W, Shiplo M, Nihilani N, Lee K. The potential risks of commonly prescribed antipsychotics: during pregnancy and lactation. Psychiatry (Edgmont). 2005;2(8):36-44.	Dupl. 4PM
213	Li M, Budin R, Fleming AS, Kapur S. Effects of novel antipsychotics, amisulpiride and aripiprazole, on maternal behavior in rats. Psychopharmacology (Berl). 2005;181(3):600-10. doi: 10.1007/s00213-005-0091-7. Epub 2005 Oct 12.	Dupl. 3PM
214	Gentile S. Prophylactic treatment of bipolar disorder in pregnancy and breastfeeding: focus on emerging mood stabilizers. Bipolar Disord. 2006;8(3):207-20. doi: 10.1111/j.1399-5618.2006.00295.x.	Dupl. 7PM
215	Mendhekar DN, Sunder KR, Andrade C. Aripiprazole use in a pregnant schizoaffective woman. Bipolar Disord. 2006;8(3):299-300. doi: 10.1111/j.1399-5618.2006.00316.x.	Dupl. 8PM
216	Shivakumar K, Mir A, McAllister VDM, O'Keane V, Aitchison KJ. Aripiprazole in psychotic depression: Four case reports. Clin Neuropsychiatry. 2006;3(5):332-337.	No preg/Lact
217	Farley CM, Baella SA, Wacan JJ, Crawford CA, McDougall SA. Pre- and postsynaptic actions of a partial D2 receptor agonist in reserpinized young rats: longevity of agonistic effects. Brain Res. 2006;1124(1):37-44. doi: 10.1016/j.brainres.2006.09.068. Epub 2006 Oct 27.	Animal
218	Schlotterbeck P, Leube D, Kircher T, Hiemke C, Gründer G. Aripiprazole in human milk. Int J Neuropsychopharmacol. 2007;10(3):433. doi: 10.1017/S1461145707007602.	No preg/Lact
219	Mir A, Shivakumar K, Williamson RJ, McAllister V, O'Keane V, Aitchison KJ. Change in sexual dysfunction with aripiprazole: a switching or add-on study. J Psychopharmacol. 2008;22(3):244-53. doi: 10.1177/0269881107082901.	No preg/Lact
220	Macaluso M, Khan AY. A 19-year-old Black woman with psychotic and depressive symptoms. Psychiatr Annals. 2008;38(5):312-314.	No preg/Lact
221	Donohoe DR, Weeks K, Aamodt EJ, Dwyer DS. Antipsychotic drugs alter neuronal development including ALM neuroblast migration and PLM axonal outgrowth in Caenorhabditis elegans. Int J Dev Neurosci. 2008;26(3-4):371-80. doi: 10.1016/j.ijdevneu.2007.08.021. Epub 2008 Jan 20.	Dupl. 10PM
222	Mervak B, Collins J, Valenstein M. Case report of aripiprazole usage during pregnancy. Arch Womens Ment Health. 2008;11(3):249-50. doi: 10.1007/s00737-008-0022-9.	Dupl. 11PM
223	Einarson A, Boskovic R. Use and safety of antipsychotic drugs during pregnancy. J Psychiatr Pract. 2009;15(3):183-92. doi: 10.1097/01.pra.0000351878.45260.94.	Dupl. 13PM
224	de Leon J, Greenlee B, Barber J, Sabaawi M, Singh NN. Practical guidelines for the use of new generation antipsychotic drugs (except clozapine) in adult individuals with intellectual disabilities. Res Dev Disabil. 2009;30(4):613-69. doi: 10.1016/j.ridd.2008.10.010. Epub 2008 Dec 11.	Dupl. 12PM
225	McCauley-Elsom K, Gurvich C, Elsom SJ, Kulkarni J. Antipsychotics in pregnancy. J Psychiatr Ment Health Nurs. 2010;17(2):97-104. doi: 10.1111/j.1365-2850.2009.01481.x.	Dupl. 14PM
226	Lutz UC, Hiemke C, Wiatr G, Farger G, Arand J, Wildgruber D. Aripiprazole in pregnancy and lactation: a case report. J Clin Psychopharmacol. 2010;30(2):204-5. doi: 10.1097/JCP.0b013e3181d27c7d.	Dupl. 15PM
227	Der-Ghazarian T, Charntikov S, Varela FA, Crawford CA, McDougall SA. Effects of repeated and acute aripiprazole or haloperidol treatment on dopamine synthesis in the dorsal striatum of young rats: comparison to adult rats. J Neural Transm (Vienna). 2010;117(5):573-83. doi: 10.1007/s00702-010-0396-5. Epub 2010 Apr 6.	Animal
228	Savaş E, Özovacı A, Kokaçya MH, Altındağ A, Vırıt O. Antidepresanla Birlikte Atipik Antipsikotik Ekleme Tedavisi Kullanan ve Kullanmayan Obsesif Kompulsif Bozukluk Hastalarında Metabolik Sendrom Sıklığı: Naturalistik, Tanımlayıcı Bir Ön Çalışma. Klinik Psikofarmakoloji Bülteni-Bulletin of Clinical Psychopharmacology. 2010;20(4):307-313. doi:10.1080/10177833.2010.11790677.	No preg/Lact
229	Aitchison KJ, Mir A, Shivakumar K, McAllister VD, O'Keane V, McCrone P. Costs and outcomes associated with an aripiprazole add-on or switching open-label study in psychosis. J Psychopharmacol. 2011;25(5):675-84. doi: 10.1177/0269881109358198. Epub 2010 Feb 22.	No preg/Lact
230	Watanabe N, Kasahara M, Sugibayashi R, Nakamura T, Nakajima K, Watanabe O, Murashima A. Perinatal use of aripiprazole: a case report. J Clin Psychopharmacol. 2011;31(3):377-9. doi: 10.1097/JCP.0b013e318218c400.	Dupl. 18PM
231	Nguyen T, Teoh S, Hackett LP, Ilett K. Placental transfer of aripiprazole. Aust N Z J Psychiatry. 2011;45(6):500-1. doi: 10.3109/00048674.2011.566547. Epub 2011 Mar 17.	Dupl. 16PM
232	Gentile S, Tofani S, Bellantuono C. Aripiprazole and pregnancy: a case report and literature review. J Clin Psychopharmacol. 2011;31(4):531-2. doi: 10.1097/JCP.0b013e318222bc65.	Dupl. 21PM
233	Matsuda N, Hashimoto N, Kusumi I, Ito K, Koyama T. Tardive laryngeal dystonia associated with aripiprazole monotherapy. J Clin Psychopharmacol. 2012;32(2):297-8. doi: 10.1097/JCP.0b013e318248590f.	No preg/Lact
234	Richtand NM, Ahlbrand R, Horn P, Tambyraja R, Grainger M, Bronson SL, McNamara RK. Fluoxetine and aripiprazole treatment following prenatal immune activation exert longstanding effects on rat locomotor response. Physiol Behav. 2012;106(2):171-7. doi: 10.1016/j.physbeh.2012.02.004. Epub 2012 Feb 9.	Dupl.23PM
235	Potter PO, John N, Coffey DB. Onset of abnormal movements and cardiovascular symptoms after acute change in complex polypharmacy in a child with attention-deficit/hyperactivity disorder and mood symptoms. J Child Adolesc Psychopharmacol. 2012;22(5):388-92. doi: 10.1089/cap.2012.2253.	Dupl. 25PM
236	Singh R, Mall GD. Hyperprolactinemia in Antipsychotic Use. Psychiatric Annals. 2012;42(10):389-392. doi:10.3928/00485713-20121003-08.	No preg/Lact
237	Rizzo R, Gulisano M, Calì PV. Oculogyric crisis: a rare extrapyramidal side effect in the treatment of Tourette syndrome. Eur Child Adolesc Psychiatry. 2012;21(10):591-2. doi: 10.1007/s00787-012-0288-3. Epub 2012 Jun 13.	No preg/Lact
238	Widschwendter CG, Hofer A. Aripiprazole use in early pregnancy: a case report. Pharmacopsychiatry. 2012;45(7):299-300. doi: 10.1055/s-0032-1312591. Epub 2012 May 30.	Dupl. 24PM
239	Handley R, Zelaya FO, Reinders AA, Marques TR, Mehta MA, O'Gorman R, Alsop DC, Taylor H, Johnston A, Williams S, McGuire P, Pariante CM, Kapur S, Dazzan P. Acute effects of single-dose aripiprazole and haloperidol on resting cerebral blood flow (rCBF) in the human brain. Hum Brain Mapp. 2013;34(2):272-82. doi: 10.1002/hbm.21436. Epub 2012 Mar 25.	Unrelated
240	Wakil L, Perea E, Penaskovic K, Stuebe A, Meltzer-Brody S. Exacerbation of psychotic disorder during pregnancy in the context of medication discontinuation. Psychosomatics. 2013;54(3):290-3. doi: 10.1016/j.psym.2012.07.003. Epub 2012 Dec 4.	Dupl. 27PM
**241**	**Nordeng H, Gjerdalen G, Brede WR, Michelsen LS, Spigset O. Transfer of aripiprazole to breast milk: a case report. J Clin Psychopharmacol. 2014;34(2):272-5. doi: 10.1097/JCP.0000000000000079.**	**Case**
242	Varela FA, Der-Ghazarian T, Lee RJ, Charntikov S, Crawford CA, McDougall SA. Repeated aripiprazole treatment causes dopamine D2 receptor up-regulation and dopamine supersensitivity in young rats. J Psychopharmacol. 2014;28(4):376-86. doi: 10.1177/0269881113504016. Epub 2013 Sep 17.	Dupl. 22C
243	Lozano R, Marin R, Santacruz MJ. Prolactin deficiency by aripiprazole. J Clin Psychopharmacol. 2014;34(4):539-40. doi: 10.1097/JCP.0000000000000151.	Dupl. 35PM
244	Windhager E, Kim SW, Saria A, Zauner K, Amminger PG, Klier CM. Perinatal use of aripiprazole: plasma levels, placental transfer, and child outcome in 3 new cases. J Clin Psychopharmacol. 2014;34(5):637-41. doi: 10.1097/JCP.0000000000000171.	Dupl. 36PM
245	John AP, Adriana S, La'Brooy JA, Piepiorka-Sokolowska D. Successful treatment of clozapine-associated restless leg syndrome with pramipexole. J Clin Psychopharmacol. 2014;34(6):764-6. doi: 10.1097/JCP.0000000000000225.	Unsuitable C

246	Bellantuono C, Di Massimo G, Mauro A, Martellini M, Nardi B. Aripiprazolo in gravidanza: una rassegna della letteratura internazionale [Aripiprazole in pregnancy: a review of literature]. Riv Psichiatr. 2015;50(1):8-11. Italian. doi: 10.1708/1794.19526.	Dupl. 46PM
247	Sharma V, Sommerdyk C, Xie B. Aripiprazole augmentation of antidepressants for postpartum depression: a preliminary report. Arch Womens Ment Health. 2015;18(1):131-4. doi: 10.1007/s00737-014-0462-3. Epub 2014 Sep 17.	Dupl. 39PM
248	Frew JR. Psychopharmacology of bipolar I disorder during lactation: a case report of the use of lithium and aripiprazole in a nursing mother. Arch Womens Ment Health. 2015;18(1):135-6. doi: 10.1007/s00737-014-0469-9. Epub 2014 Oct 29.	Dupl. 40PM
249	Silverman MA, Leung JG, Schak KM. Reply to: comment on: aripiprazole-associated hiccups: a case and closer look at the association between hiccups and antipsychotics. J Pharm Pract. 2015;28(2):134. doi: 10.1177/0897190014568680.	Opinion
205	Salau M, Adam B, Coffey BJ. Metformin in an Adolescent with Significant Weight Gain. J Child Adolesc Psychopharmacol. 2015 Sep;25(7):589-92. doi: 10.1089/cap.2015.29022.bjc.	No preg/Lact
251	Hu LY, Lee YT, Lu T, Hung MB, Hung YY. Using aripiprazole to treat new-onset hyperprolactinemia-related delusion of pregnancy. Aust N Z J Psychiatry. 2015;49(10):946. doi: 10.1177/0004867415589796. Epub 2015 Jun 22.	Dupl. 47PM
252	Bacanli A. Aripiprazole Use in Children Diagnosed with Down Syndrome and Comorbid Autism Spectrum Disorders. J Child Adolesc Psychopharmacol. 2016;26(3):306-8. doi: 10.1089/cap.2015.0193. Epub 2016 Feb 12.	No preg/Lact
253	Uguz F. Second-Generation Antipsychotics During the Lactation Period: A Comparative Systematic Review on Infant Safety. J Clin Psychopharmacol. 2016;36(3):244-52. doi: 10.1097/JCP.0000000000000491.	Dupl. 52PM
254	An FR, Yang R, Wang ZM, Ungvari GS, Ng CH, Chiu HF, Wu PP, Jin X, Li L, Lok GK, Xiang YT. Hyperprolactinemia, prolactin-related side effects and quality of life in Chinese psychiatric patients. Compr Psychiatry. 2016;71:71-76. doi: 10.1016/j.comppsych.2016.08.009. Epub 2016 Aug 24.	Dupl. 54PM
255	Kus K, Ratajczak P, Czaja N, Zaprutko T, Nowakowska E. Effect of combined administration of aripiprazole and fluoxetine on cognitive functions in female rats exposed to ethyl alcohol. Acta Neurobiol Exp (Wars). 2017;77(1):86-93. doi: 10.21307/ane-2017-039.	Dupl. 56PM
256	Ipci M, Inci SB, Akyol Ardiç Ü, Ercan ES. A Case of Asperger Syndrome With Comorbidity of Posttraumatic Stress Disorder and Selective Mutism: Significant Remission With the Combination of Aripiprazole and Eye Movement Desensitization and Reprocessing. J Clin Psychopharmacol. 2017;37(1):109-110. doi: 10.1097/JCP.0000000000000627.	No preg/Lact
257	Lin MW, Chang C, Yeh CB, Tai YM, Chang HA, Kao YC, Tzeng NS. Aripiprazole-related hyponatremia and consequent valproic acid-related hyperammonemia in one patient. Aust N Z J Psychiatry. 2017;51(3):296-297. doi: 10.1177/0004867416671416. Epub 2016 Sep 30.	No preg/Lact
258	Korade Ž, Liu W, Warren EB, Armstrong K, Porter NA, Konradi C. Effect of psychotropic drug treatment on sterol metabolism. Schizophr Res. 2017;187:74-81. doi: 10.1016/j.schres.2017.02.001. Epub 2017 Feb 12.	Dupl. 55PM
259	Park Y, Huybrechts KF, Cohen JM, Bateman BT, Desai RJ, Patorno E, Mogun H, Cohen LS, Hernandez-Diaz S. Antipsychotic Medication Use Among Publicly Insured Pregnant Women in the United States. Psychiatr Serv. 2017;68(11):1112-1119. doi: 10.1176/appi.ps.201600408. Epub 2017 Jun 15.	Dupl. 59PM
260	Hara Y, Ago Y, Taruta A, Hasebe S, Kawase H, Tanabe W, Tsukada S, Nakazawa T, Hashimoto H, Matsuda T, Takuma K. Risperidone and aripiprazole alleviate prenatal valproic acid-induced abnormalities in behaviors and dendritic spine density in mice. Psychopharmacology (Berl). 2017;234(21):3217-3228. doi: 10.1007/s00213-017-4703-9. Epub 2017 Aug 10.	Dupl. 64PM
261	Çelikkol Ç, Akça ÖF. Aripiprazole Treatment in a 9-Year-Old Child With OCD and Hypomania Symptoms Possibly Related to Herbal Medicines: A Case Report. J Clin Psychopharmacol. 2018;38(1):101-102. doi: 10.1097/JCP.0000000000000833.	No preg/Lact
262	Cuomo A, Goracci A, Fagiolini A. Aripiprazole use during pregnancy, peripartum and lactation. A systematic literature search and review to inform clinical practice. J Affect Disord. 2018;228:229-237. doi: 10.1016/j.jad.2017.12.021. Epub 2017 Dec 14.	Dupl. 68PM
263	Damkier P, Videbech P. The Safety of Second-Generation Antipsychotics During Pregnancy: A Clinically Focused Review. CNS Drugs. 2018;32(4):351-366. doi: 10.1007/s40263-018-0517-5.	Dupl. 69PM
264	Park Y, Hernandez-Diaz S, Bateman BT, Cohen JM, Desai RJ, Patorno E, Glynn RJ, Cohen LS, Mogun H, Huybrechts KF. Continuation of Atypical Antipsychotic Medication During Early Pregnancy and the Risk of Gestational Diabetes. Am J Psychiatry. 2018;175(6):564-574. doi: 10.1176/appi.ajp.2018.17040393. Epub 2018 May 7.	Dupl. 71PM
265	Lian J, Deng C. The effects of antipsychotics on the density of cannabinoid receptors in selected brain regions of male and female adolescent juvenile rats. Psychiatry Res. 2018;266:317-322. doi: 10.1016/j.psychres.2018.03.030. Epub 2018 Mar 16.	Animal
266	Galbally M, Frayne J, Watson SJ, Snellen M. Aripiprazole and pregnancy: A retrospective, multicentre study. J Affect Disord. 2018;238:593-596. doi: 10.1016/j.jad.2018.06.004. Epub 2018 Jun 14.	Dupl. 72PM
267	Pan B, Lian J, Deng C. Chronic antipsychotic treatment differentially modulates protein kinase A- and glycogen synthase kinase 3 beta-dependent signaling pathways, N-methyl-D-aspartate receptor and γ-aminobutyric acid A receptors in nucleus accumbens of juvenile rats. J Psychopharmacol. 2018;32(11):1252-1263. doi: 10.1177/0269881118788822. Epub 2018 Aug 23.	Dupl. 42C
268	Uguz F. Antipsychotic Use During Pregnancy and the Risk of Gestational Diabetes Mellitus: A Systematic Review. J Clin Psychopharmacol. 2019;39(2):162-167. doi: 10.1097/JCP.0000000000001002.	Dupl. 75PM
269	Lian J, Deng C. Early antipsychotic exposure affects NMDA and GABAA receptor binding in the brains of juvenile rats. Psychiatry Res. 2019;273:739-745. doi: 10.1016/j.psychres.2019.02.001. Epub 2019 Feb 2.	Dupl. 79PM
270	Genaro-Mattos TC, Allen LB, Anderson A, Tallman KA, Porter NA, Korade Z, Mirnics K. Maternal aripiprazole exposure interacts with 7-dehydrocholesterol reductase mutations and alters embryonic neurodevelopment. Mol Psychiatry. 2019;24(4):491-500. doi: 10.1038/s41380-019-0368-6. Epub 2019 Feb 11.	Dupl. 76PM
271	Mereu A, Serra G, Vicari S, Zanna V. Bipolar Disorder in a Young Woman With Preexisting Rapid-Onset Dystonia-Parkinsonism and Successful Treatment With Clozapine and Lithium. J Clin Psychopharmacol. 2019 May/Jun;39(3):277-279. doi: 10.1097/JCP.0000000000001045.	No preg/Lact
272	Takahashi K, Nakagawasai O, Sakuma W, Nemoto W, Odaira T, Lin JR, Onogi H, Srivastava LK, Tan-No K. Prenatal treatment with methylazoxymethanol acetate as a neurodevelopmental disruption model of schizophrenia in mice. Neuropharmacology. 2019;150:1-14. doi: 10.1016/j.neuropharm.2019.02.034. Epub 2019 Mar 1.	Dupl. 77PM
273	Choi HJ, Im SJ, Park HR, Park S, Kim CE, Ryu S. Long-term Effects of Aripiprazole Treatment during Adolescence on Cognitive Function and Dopamine D2 Receptor Expression in Neurodevelopmentally Normal Rats. Clin Psychopharmacol Neurosci. 2019;17(3):400-408. doi: 10.9758/cpn.2019.17.3.400.	Animal
274	Anmella G, Pacchiarotti I, Cubała WJ, Dudek D, Maina G, Thomas P, Vieta E. Expert advice on the management of valproate in women with bipolar disorder at childbearing age. Eur Neuropsychopharmacol. 2019;29(11):1199-1212. doi: 10.1016/j.euroneuro.2019.09.007. Epub 2019 Oct 4.	Dupl. 82PM
275	Beaulieu AM, Tabasky E, Osser DN. The psychopharmacology algorithm project at the Harvard South Shore Program: An algorithm for adults with obsessive-compulsive disorder. Psychiatry Res. 2019;281:112583. doi: 10.1016/j.psychres.2019.112583. Epub 2019 Sep 27.	Dupl. 83PM
276	Smirnova, D., & Fountoulakis, K. (2020). Evidence-based outcome for the interventions in childhood-onset schizophrenia. In A. Shrivastava & A. De Sousa (Eds.), Schizophrenia treatment outcomes: An evidence-based approach to recovery (pp. 319–337). Cham CH: Springer Nature Switzerland AG. doi:10.1007/978-3-030-19847-3_28.	Review
277	Uguz F. Pharmacological prevention of mood episodes in women with bipolar disorder during the perinatal period: A systematic review of current literature. Asian J Psychiatr. 2020;52:102145. doi: 10.1016/j.ajp.2020.102145. Epub 2020 May 12.	Dupl. 89PM
278	Genaro-Mattos TC, Anderson A, Allen LB, Tallman KA, Porter NA, Korade Z, Mirnics K. Maternal cariprazine exposure inhibits embryonic and postnatal brain cholesterol biosynthesis. Mol Psychiatry. 2020;25(11):2685-2694. doi: 10.1038/s41380-020-0801-x. Epub 2020 Jun 5.	Dupl. 88PM
279	Wichniak A, Siwek M, Rymaszewska J, Janas-Kozik M, Wolańczyk T, Bieńkowski P, Dudek D, Heitzman J, Szulc A, Samochowiec J. The position statement of the Working Group of the Polish Psychiatric Association on the use of D2/D3 dopamine receptor partial agonists in special populations. Psychiatr Pol. 2021;55(5):967-987. English, Polish. doi: 10.12740/PP/140287. Epub 2021 Oct 31.	Dupl. 102PM
280	Procyshyn RM, Bezchlibnyk-Butler KZ, Jeffries JJ (eds.). Clinical Handbook of Psychotropic Drugs, 24th ed. Boston, Mass.: Hogrefe, 2021.	Review
281	Medved S, Bajs Janović M, Štimac Z, Mihaljević-Peleš A. Add-on Oxytocin in the Treatment of Postpartum Acute Schizophrenia: A Case Report. J Psychiatr Pract. 2021;27(4):326-332. doi: 10.1097/PRA.0000000000000557.	Dupl. 99PM
282	Freeman MP, Viguera AC, Góez-Mogollón L, Young AV, Caplin PS, McElheny SA, Church TR, Chitayat D, Hernández-Díaz S, Cohen LS. Reproductive safety of aripiprazole: data from the Massachusetts General Hospital National Pregnancy Registry for Atypical Antipsychotics. Arch Womens Ment Health. 2021;24(4):659-667. doi: 10.1007/s00737-021-01115-6. Epub 2021 Mar 12. Erratum in: Arch Womens Ment Health. 2021;24(4):669-670. doi: 10.1007/s00737-021-01133-4.	Dupl. 93PM
283	Freeman MP, Viguera AC, Góez-Mogollón L, Young AV, Caplin PS, McElheny SA, Church TR, Chitayat D, Hernández-Díaz S, Cohen LS. Correction to: Reproductive safety of aripiprazole: data from the Massachusetts General Hospital National Pregnancy Registry for Atypical Antipsychotics. Arch Womens Ment Health. 2021;24(4):669-670. doi: 10.1007/s00737-021-01133-4. Erratum for: Arch Womens Ment Health. 2021;24(4):659-667. doi: 10.1007/s00737-021-01115-6.	Dupl. 93PM

284	Román V, Adham N, Foley AG, Hanratty L, Farkas B, Lendvai B, Kiss B. Cariprazine alleviates core behavioral deficits in the prenatal valproic acid exposure model of autism spectrum disorder. Psychopharmacology (Berl). 2021;238(9):2381-2392. doi: 10.1007/s00213-021-05851-6. Epub 2021 Jul 15.	Dupl. 98PM
285	Korade Z, Heffer M, Mirnics K. Medication effects on developmental sterol biosynthesis. Mol Psychiatry. 2022;27(1):490-501. doi: 10.1038/s41380-021-01074-5. Epub 2021 Apr 5.	Dupl. 95PM
286	Llorca PM, Nuss P, Fakra É, Alamome I, Drapier D, El Hage W, Jardri R, Mouchabac S, Rabbani M, Simon N, Vacheron MN, Azorin JM. Place of the partial dopamine receptor agonist aripiprazole in the management of schizophrenia in adults: a Delphi consensus study. BMC Psychiatry. 2022;22(1):364. doi: 10.1186/s12888-022-04008-9.	Dupl. 109PM
287	Lian J, Han M, Su Y, Hodgson J, Deng C. The long-lasting effects of early antipsychotic exposure during juvenile period on adult behaviours - A study in a poly I:C rat model. Pharmacol Biochem Behav. 2022;219:173453. doi: 10.1016/j.pbb.2022.173453. Epub 2022 Aug 25.	Dupl. 113PM
288	Lee MY, Hsieh CP, Chan MH, Chen HH. Beneficial effects of atypical antipsychotics on object recognition deficits after adolescent toluene exposure in mice: involvement of 5-HT1A receptors. Am J Drug Alcohol Abuse. 2022;48(6):673-683. doi: 10.1080/00952990.2022.2122484. Epub 2022 Sep 22.	Animal
289	Samalin L, Arnould A, Boudieu L, Henry C, Haffen E, Drapier D, Anmella G, Pacchiarotti I, Vieta E, Belzeaux R, Llorca PM. Avis d'experts français sur la prise en charge des femmes en âge de procréer et enceintes souffrant d'un trouble bipolaire traitées par valproate [French Expert advice on the management of valproate in childbearing and pregnant women with bipolar disorder]. Encephale. 2022;48(6):624-631. French. doi: 10.1016/j.encep.2022.07.005. Epub 2022 Oct 17.	Review
290	de Oliveira Ferreira E, Pessoa Gomes JM, Neves KRT, Lima FAV, de Barros Viana GS, de Andrade GM. Maternal treatment with aripiprazole prevents the development of a valproic acid-induced autism-like phenotype in juvenile male mice. Behav Pharmacol. 2023;34(2-3):154-168. doi: 10.1097/FBP.0000000000000718. Epub 2023 Feb 10.	Dupl. 117PM
291	Naughton S, O'Hara K, Nelson J, Keightley P. Aripiprazole, brexpiprazole, and cariprazine can affect milk supply: Advice to breastfeeding mothers. Australas Psychiatry. 2023;31(2):201-204. doi: 10.1177/10398562231159510. Epub 2023 Feb 24.	Dupl. 116PM
292	Sharma V, Al-Farayedhi M. Postpartum-onset obsessive-compulsive disorder: A harbinger of bipolar disorder. Bipolar Disord. 2023;25(3):254-256. doi: 10.1111/bdi.13303. Epub 2023 Jan 25.	Unsuitable C
293	Sahoo MK, Biswas H, Grover S. Safety Profile of Aripiprazole During Pregnancy and Lactation: Report of 2 Cases. Türk Psikiyatri Derg. 2023;34(2):133-135. doi: 10.5080/u26681.	Dupl. 122PM
294	Kumon H, Yoshino Y, Ozaki T, Funahashi Y, Mori H, Ueno M, Ozaki Y, Yamazaki K, Ochi S, Iga JI, Ueno SI. Gestational exposure to haloperidol changes Cdkn1a and Apaf1 mRNA expressions in mouse hippocampus. Brain Res Bull. 2023;199:110662. doi: 10.1016/j.brainresbull.2023.110662. Epub 2023 May 6.	Dupl. 120PM
295	Koch MT, Carlson HE, Kazimi MM, Correll CU. Antipsychotic-Related Prolactin Levels and Sexual Dysfunction in Mentally Ill Youth: A 3-Month Cohort Study. J Am Acad Child Adolesc Psychiatry. 2023;62(9):1021-1050. doi: 10.1016/j.jaac.2023.03.007. Epub 2023 Mar 15.	Dupl. 119PM
296	Jiang Y, Zhou L, Shen Y, Zhou Q, Ji Y, Zhu H. Safety assessment of Brexpiprazole: Real-world adverse event analysis from the FAERS database. J Affect Disord. 2024;346:223-229. doi: 10.1016/j.jad.2023.11.025. Epub 2023 Nov 11.	Dupl. 125PM
297	Pinci C, Bianciardi E, Sferra I, Castellani G, Santini R, Siracusano A, Niolu C. Switching from paliperidone palmitate 3-monthly long-acting injection to oral aripiprazole in a pregnant woman with schizophrenia: a case report and short review. Riv Psichiatr. 2024;59(2):75-79. doi: 10.1708/4259.42361.	Dupl. 131PM
298	Stachura A, Banaszek Ł, Jurkin K, Święcicki Ł. Vitamin B12 overdose may trigger the onset of mixed-state bipolar disorder: A case report. Bipolar Disord. 2024;26(3):293-295. doi: 10.1111/bdi.13424. Epub 2024 Mar 21.	No preg/Lact
299	Wang X, Li Z, Kuai S, Wang X, Chen J, Yang Y, Qin L. Correlation between desynchrony of hippocampal neural activity and hyperlocomotion in the model mice of schizophrenia and therapeutic effects of aripiprazole. CNS Neurosci Ther. 2024;30(5):e14739. doi: 10.1111/cns.14739.	Animal
300	Ishikawa T, Sakai T, Iwama N, Obara R, Morishita K, Adomi M, Noda A, Ishikuro M, Kikuchi S, Kobayashi N, Tomita H, Saito M, Nishigori H, Kuriyama S, Mano N, Obara T. Association between exposure to atypical antipsychotics during pregnancy and risk of miscarriage. Acta Psychiatr Scand. 2024;150(6):562-572. doi: 10.1111/acps.13755. Epub 2024 Sep 5.	Dupl. 139PM
301	Song SH, Hayirli TC, Shore O, Coconcea C, Keshavan M. Atypical presentation of schizophrenia with ablution avoidance: A case report. Schizophr Res. 2024;272:96-97. doi: 10.1016/j.schres.2024.08.019. Epub 2024 Aug 28.	No preg/Lact
302	Zheng J, Zhang Z, Liang Y, Wu Q, Din C, Wang Y, Ma L, Su L. Risk of congenital anomalies associated with psychotropic medications: a review of neonatal reports in the FDA adverse event reporting System (FAERS). Arch Womens Ment Health. 2024. doi: 10.1007/s00737-024-01540-3. Epub ahead of print Dec 23.	Dupl. 141PM
303	Wang E, Liu Y, Wang Y, Han X, Zhou Y, Zhang L, Tang Y. Comparative Safety of Antipsychotic Medications and Mood Stabilizers During Pregnancy: A Systematic Review and Network Meta-analysis of Congenital Malformations and Prenatal Outcomes. CNS Drugs. 2025;39(1):1-22. doi: 10.1007/s40263-024-01131-x. Epub 2024 Nov 11.	Dupl. 140PM
304	Cho H, Jo H, Jeong YD, Jang W, Park J, Yim Y, Lee K, Lee H, Lee S, Fond G, Boyer L, Pizzol D, Jung J, Yon DK. Antipsychotic use during pregnancy and outcomes in pregnant individuals and newborns. J Affect Disord. 2025;373:495-504. doi: 10.1016/j.jad.2024.12.102. Epub 2025 Jan 2.	Dupl. 142PM
	Scopus TITLE(cariprazine OR brexpiprazole OR aripiprazole) AND TITLE-ABS-KEY(pregnancy OR lactation OR breastfeeding) 23.6.2025 → 80 results	
305	Gupta S, Masand P. Aripiprazole: review of its pharmacology and therapeutic use in psychiatric disorders. Ann Clin Psychiatry. 2004;16(3):155-66. doi: 10.1080/10401230490487007.	Review
306	Schutte, M.K., Bijl, D. Aripiprazole (Abilify®) for the treatment of schizophrenia | Aripiprazol (Abilify®), behandeling schizofrenie. Geneesmiddelenbulletin. 2005;39(8):85-87. Dutch	Opinion
307	[Editorial]. Aripiprazole: Just another neuroleptic. Prescrire International, 2005;14(79):163-167	Opinion
308	Ginsberg, D.L. Addition of aripiprazole for risperidone-induced hyperprolactinemia. Primary Psychiatry. 2005;12(10):34-35	Opinion
309	Mendhekar DN, Sunder KR, Andrade C. Aripiprazole use in a pregnant schizoaffective woman. Bipolar Disord. 2006;8(3):299-300. doi: 10.1111/j.1399-5618.2006.00316.x.	Dupl. 8PM
310	Mendhekar DN, Sharma JB, Srilakshmi P. Use of aripiprazole during late pregnancy in a woman with psychotic illness. Ann Pharmacother. 2006;40(3):575. doi: 10.1345/aph.1G507. Epub 2006 Feb 7.	Dupl. 5PM
311	Mervak B, Collins J, Valenstein M. Case report of aripiprazole usage during pregnancy. Arch Womens Ment Health. 2008;11(3):249-50. doi: 10.1007/s00737-008-0022-9.	Dupl. 11PM
312	Pae C-U. A review of the safety and tolerability of aripiprazole. Expert Opin Drug Saf. 2009;8(3):373-86. doi: 10.1517/14740330902835493.	Review
313	Lutz UC, Hiemke C, Wiatr G, Farger G, Arand J, Wildgruber D. Aripiprazole in pregnancy and lactation: a case report. J Clin Psychopharmacol. 2010;30(2):204-5. doi: 10.1097/JCP.0b013e3181d27c7d.	Dupl. 15PM
314	Nguyen T, Teoh S, Hackett LP, Ilett K. Placental transfer of aripiprazole. Aust N Z J Psychiatry. 2011;45(6):500-1. doi: 10.3109/00048674.2011.566547. Epub 2011 Mar 17.	Dupl. 16PM
315	Watanabe N, Kasahara M, Sugibayashi R, Nakamura T, Nakajima K, Watanabe O, Murashima A. Perinatal use of aripiprazole: a case report. J Clin Psychopharmacol. 2011;31(3):377-9. doi: 10.1097/JCP.0b013e318218c400.	Dupl. 18PM
316	Gentile S, Tofani S, Bellantuono C. Aripiprazole and pregnancy: a case report and literature review. J Clin Psychopharmacol. 2011;31(4):531-2. doi: 10.1097/JCP.0b013e318222bc65.	Dupl. 21PM
317	Guillemot J, Lukaszewski MA, Montel V, Delahaye F, Mayeur S, Laborie C, Dickes-Coopman A, Dutriez-Casteloot I, Lesage J, Breton C, Vieau D. Influence of prenatal undernutrition on the effects of clozapine and aripiprazole in the adult male rats: relevance to a neurodevelopmental origin of schizophrenia? Eur J Pharmacol. 2011;667(1-3):402-9. doi: 10.1016/j.ejphar.2011.04.011. Epub 2011 Apr 15.	Dupl. 17PM
318	Park MH, Han C, Pae CU, Lee SJ, Patkar AA, Masand PS, Fleischhacker WW. Aripiprazole treatment for patients with schizophrenia: from acute treatment to maintenance treatment. Expert Rev Neurother. 2011;11(11):1541-52. doi: 10.1586/ern.11.151.	Review
319	Czernikiewicz, A. Treatment of bipolar disorder - Use of aripiprazole. Part II - Special populations | Terapia choroby afektywnej dwubiegunowej - Zastosowanie arypiprazolu. Część II - Szczególne sytuacje kliniczne. Wiadomosci Psychiatryczne. 2011;14(3):202-204. Polish.	Review
320	Richtand NM, Ahlbrand R, Horn P, Tambyraja R, Grainger M, Bronson SL, McNamara RK. Fluoxetine and aripiprazole treatment following prenatal immune activation exert longstanding effects on rat locomotor response. Physiol Behav. 2012;106(2):171-7. doi: 10.1016/j.physbeh.2012.02.004. Epub 2012 Feb 9.	Dupl.23PM
321	Widschwendter CG, Hofer A. Aripiprazole use in early pregnancy: a case report. Pharmacopsychiatry. 2012;45(7):299-300. doi: 10.1055/s-0032-1312591. Epub 2012 May 30.	Dupl. 24PM
322	Singh KP, Tripathi N. Prenatal exposure of a novel antipsychotic aripiprazole: impact on maternal, fetal and postnatal body weight modulation in rats. Curr Drug Saf. 2014;9(1):43-8. doi: 10.2174/15748863113086660061.	Dupl. 32PM
323	Nowakowska E, Kus K, Ratajczak P, Cichocki M, Woźniak A. The influence of aripiprazole, olanzapine and enriched environment on depressant-like behavior, spatial memory dysfunction and hippocampal level of BDNF in prenatally stressed rats. Pharmacol Rep. 2014;66(3):404-11. doi: 10.1016/j.pharep.2013.12.008. Epub 2014 Apr 3.	Dupl. 34PM

324	Lozano R, Marin R, Santacruz MJ. Prolactin deficiency by aripiprazole. J Clin Psychopharmacol. 2014;34(4):539-40. doi: 10.1097/JCP.0000000000000151.	Dupl. 35PM
325	Nordeng H, Gjerdalen G, Brede WR, Michelsen LS, Spigset O. Transfer of aripiprazole to breast milk: a case report. J Clin Psychopharmacol. 2014;34(2):272-5. doi: 10.1097/JCP.0000000000000079.	Dupl. 31Ps
326	Windhager E, Kim SW, Saria A, Zauner K, Amminger PG, Klier CM. Perinatal use of aripiprazole: plasma levels, placental transfer, and child outcome in 3 new cases. J Clin Psychopharmacol. 2014;34(5):637-41. doi: 10.1097/JCP.0000000000000171.	Dupl. 36PM
327	Pirec V, Mehta A, Shoush S. Aripiprazole combined with other psychotropic drugs in pregnancy: two case reports. Isr J Psychiatry Relat Sci. 2014;51(2):135-6.	Dupl. 42PM
328	Gentile S. A safety evaluation of aripiprazole for treating schizophrenia during pregnancy and puerperium. Expert Opin Drug Saf. 2014;13(12):1733-42. doi: 10.1517/14740338.2014.951325. Epub 2014 Aug 19.	Dupl. 38PM
329	Bellantuono C, Di Massimo G, Mauro A, Martellini M, Nardi B. Aripiprazolo in gravidanza: una rassegna della letteratura internazionale [Aripiprazole in pregnancy: a review of literature]. Riv Psichiatr. 2015;50(1):8-11. Italian. doi: 10.1708/1794.19526.	Dupl. 46PM
330	Frew JR. Psychopharmacology of bipolar I disorder during lactation: a case report of the use of lithium and aripiprazole in a nursing mother. Arch Womens Ment Health. 2015;18(1):135-6. doi: 10.1007/s00737-014-0469-9. Epub 2014 Oct 29.	Dupl. 40PM
331	Sharma V, Sommerdyk C, Xie B. Aripiprazole augmentation of antidepressants for postpartum depression: a preliminary report. Arch Womens Ment Health. 2015;18(1):131-4. doi: 10.1007/s00737-014-0462-3. Epub 2014 Sep 17.	Dupl. 39PM
332	Bellet F, Beyens MN, Bernard N, Beghin D, Elefant E, Vial T. Exposure to aripiprazole during embryogenesis: a prospective multicenter cohort study. Pharmacoepidemiol Drug Saf. 2015;24(4):368-80. doi: 10.1002/pds.3749. Epub 2015 Feb 12.	Dupl. 44PM
333	Ennis ZN, Damkier P. Pregnancy exposure to olanzapine, quetiapine, risperidone, aripiprazole and risk of congenital malformations. A systematic review. Basic Clin Pharmacol Toxicol. 2015;116(4):315-20. doi: 10.1111/bcpt.12372. Epub 2015 Jan 28.	Dupl. 43PM
334	Hu LY, Lee YT, Lu T, Hung MB, Hung YY. Using aripiprazole to treat new-onset hyperprolactinemia-related delusion of pregnancy. Aust N Z J Psychiatry. 2015;49(10):946. doi: 10.1177/0004867415589796. Epub 2015 Jun 22.	Dupl. 47PM
335	Lee SY, Min JA, Lee IG, Kim JJ. Clinical Usefulness of Aripiprazole and Lamotrigine in Schizoaffective Presentation of Tuberous Sclerosis. Clin Psychopharmacol Neurosci. 2016;14(3):305-10. doi: 10.9758/cpn.2016.14.3.305.	No preg/Lact
336	Hussar DA, Shatynski R. Brexpiprazole, cariprazine hydrochloride, and flibanserin. J Am Pharm Assoc (2003). 2016;56(2):211-4. doi: 10.1016/j.japh.2016.02.003.	Opinion
337	Ratajczak P, Kus K, Giermaziak W, Nowakowska E. The influence of aripiprazole and olanzapine on the anxiolytic-like effect observed in prenatally stressed rats (animal model of schizophrenia) exposed to the ethyl alcohol. Pharmacol Rep. 2016;68(2):415-22. doi: 10.1016/j.pharep.2015.10.010. Epub 2015 Nov 10.	Dupl. 50PM
338	Ratajczak P, Kus K, Gołembiowska K, Noworyta-Sokołowska K, Woźniak A, Zaprutko T, Nowakowska E. The influence of aripiprazole and olanzapine on neurotransmitters level in frontal cortex of prenatally stressed rats. Environ Toxicol Pharmacol. 2016;46:122-130. doi: 10.1016/j.etap.2016.07.007. Epub 2016 Jul 18.	Dupl. 53PM
339	Kus K, Ratajczak P, Czaja N, Zaprutko T, Nowakowska E. Effect of combined administration of aripiprazole and fluoxetine on cognitive functions in female rats exposed to ethyl alcohol. Acta Neurobiol Exp (Wars). 2017;77(1):86-93. doi: 10.21307/ane-2017-039.	Dupl. 56PM
340	Parikh NB, Robinson DM, Clayton AH. Clinical role of brexpiprazole in depression and schizophrenia. Ther Clin Risk Manag. 2017;13:299-306. doi: 10.2147/TCRM.S94060.	Review
341	Morin C, Chevalier I. Severe Hypernatremic Dehydration and Lower Limb Gangrene in an Infant Exposed to Lamotrigine, Aripiprazole, and Sertraline in Breast Milk. Breastfeed Med. 2017;12(6):377-380. doi: 10.1089/bfm.2017.0031. Epub 2017 May 8.	Dupl. 58PM
342	Hara Y, Ago Y, Taruta A, Hasebe S, Kawase H, Tanabe W, Tsukada S, Nakazawa T, Hashimoto H, Matsuda T, Takuma K. Risperidone and aripiprazole alleviate prenatal valproic acid-induced abnormalities in behaviors and dendritic spine density in mice. Psychopharmacology (Berl). 2017;234(21):3217-3228. doi: 10.1007/s00213-017-4703-9. Epub 2017 Aug 10.	Dupl. 64PM
443	Sakai T, Ohtsu F, Mori C, Tanabe K, Goto N. Signal of Miscarriage with Aripiprazole: A Disproportionality Analysis of the Japanese Adverse Drug Event Report Database. Drug Saf. 2017;40(11):1141-1146. doi: 10.1007/s40264-017-0560-z.	Dupl. 61PM
444	Yskes R, Thomas R, Nagalla ML. A Case of Decreased Milk Production Associated With Aripiprazole. Prim Care Companion CNS Disord. 2018;20(6):18l02303. doi: 10.4088/PCC.18l02303.	Dupl. 73PM
345	Drazanova E, Ruda-Kucerova J, Kratka L, Horska K, Demlova R, Starcuk Z Jr, Kasparek T. Poly(I:C) model of schizophrenia in rats induces sex-dependent functional brain changes detected by MRI that are not reversed by aripiprazole treatment. Brain Res Bull. 2018;137:146-155. doi: 10.1016/j.brainresbull.2017.11.008. Epub 2017 Nov 16.	Dupl. 65PM
346	Cuomo A, Goracci A, Fagiolini A. Aripiprazole use during pregnancy, peripartum and lactation. A systematic literature search and review to inform clinical practice. J Affect Disord. 2018;228:229-237. doi: 10.1016/j.jad.2017.12.021. Epub 2017 Dec 14.	Dupl. 68PM
347	Suzuki H, Hibino H, Inoue Y, et al. A patient with schizophrenia who lactated owing to mastopathy during aripiprazole once-monthly 300 mg treatment. International Medical Journal: IMJ 2018;25:90-1.	Dupl. 38C
348	Ecker-Schlipf, B. Antipsychotics: Is aripiprazole suitable during pregnancy and breastfeeding? | Aripiprazol geeignet in Schwangerschaft und Stillzeit? Psychopharmakotherapie. 2018;25(2):86-87. German.	Review
349	Ratajczak P, Kus K, Skurzyńska M, Nowakowska E. The influence of aripiprazole and venlafaxine on the antidepressant-like effect observed in prenatally stressed rats (animal model of depression). Hum Exp Toxicol. 2018;37(9):972-982. doi: 10.1177/0960327117747023. Epub 2017 Dec 14.	Dupl. 66PM
350	Galbally M, Frayne J, Watson SJ, Snellen M. Aripiprazole and pregnancy: A retrospective, multicentre study. J Affect Disord. 2018;238:593-596. doi: 10.1016/j.jad.2018.06.004. Epub 2018 Jun 14.	Dupl. 72PM
351	Walker T, Coursey C, Duffus ALJ. Low Dose of Abilify (Aripiprazole) in Combination With Effexor XR (Venlafaxine HCl) Resulted in Cessation of Lactation: A Case Report. Clinical Lactation. 2019;10(2):56-59. doi: 10.1891/2158-0782.10.2.56.	Dupl. 44C
352	Genaro-Mattos TC, Allen LB, Anderson A, Tallman KA, Porter NA, Korade Z, Mirnics K. Maternal aripiprazole exposure interacts with 7-dehydrocholesterol reductase mutations and alters embryonic neurodevelopment. Mol Psychiatry. 2019;24(4):491-500. doi: 10.1038/s41380-019-0368-6. Epub 2019 Feb 11.	Dupl. 76PM
353	Cuomo A, Beccarini Crescenzi B, Goracci A, Bolognesi S, Giordano N, Rossi R, Facchi E, Neal SM, Fagiolini A. Drug safety evaluation of aripiprazole in bipolar disorder. Expert Opin Drug Saf. 2019;18(6):455-463. doi: 10.1080/14740338.2019.1617847. Epub 2019 May 17.	Dupl. 78PM
354	Ballester-Gracia I, Pérez-Almarcha M, Galvez-Llompart A, Hernandez-Viadel M. Use of long acting injectable aripiprazole before and through pregnancy in bipolar disorder: a case report. BMC Pharmacol Toxicol. 2019;20(1):52. doi: 10.1186/s40360-019-0330-x.	Dupl. 80PM
355	Kumar A, Singh H, Mishra A, Mishra AK. Aripiprazole: An FDA Approved Bioactive Compound to Treat Schizophrenia- A Mini Review. Curr Drug Discov Technol. 2020;17(1):23-29. doi: 10.2174/1570163815666181008151718.	Review
356	Ratajczak P, Kus K, Murawiecka P, Słodzińska I, Zaprutko T, Kopciuch D, Paczkowska A, Nowakowska E. Memory deterioration based on the tobacco smoke exposure and methylazoxymethanol acetate administration vs. aripiprazole, olanzapine and enrichment environment conditions. Pharmacol Biochem Behav. 2020;189:172855. doi: 10.1016/j.pbb.2020.172855. Epub 2020 Jan 15.	Dupl. 86PM
357	Wong MMC, Chung AKK, Yeung TMH, Wong DTW, Lee CK, Lai E, Chan GFY, Mak GKL, Wong JOY, Ng RMK, Tam KL, Mak KY. Consensus statements on the clinical usage and characteristics of aripiprazole for Hong Kong. Intern Med J. 2020;50(Suppl 3):6-14. doi: 10.1111/imj.14896.	Review
358	Genaro-Mattos TC, Anderson A, Allen LB, Tallman KA, Porter NA, Korade Z, Mirnics K. Maternal cariprazine exposure inhibits embryonic and postnatal brain cholesterol biosynthesis. Mol Psychiatry. 2020;25(11):2685-2694. doi: 10.1038/s41380-020-0801-x. Epub 2020 Jun 5.	Dupl. 88PM
359	Fernández-Abascal B, Recio-Barbero M, Sáenz-Herrero M, Segarra R. Long-acting injectable aripiprazole in pregnant women with schizophrenia: a case-series report. Ther Adv Psychopharmacol. 2021;11:2045125321991277. doi: 10.1177/2045125321991277.	Dupl.103PM
360	Komaroff A. Aripiprazole and lactation failure: The importance of shared decision making. A case report. Case Rep Womens Health. 2021;30:e00308. doi: 10.1016/j.crwh.2021.e00308.	Dupl. 94PM
361	Zheng L, Tang S, Tang R, Xu M, Jiang X, Wang L. Dose Adjustment of Quetiapine and Aripiprazole for Pregnant Women Using Physiologically Based Pharmacokinetic Modeling and Simulation. Clin Pharmacokinet. 2021;60(5):623-635. doi: 10.1007/s40262-020-00962-3. Epub 2020 Nov 30.	Dupl. 91PM
362	Freeman MP, Viguera AC, Góez-Mogollón L, Young AV, Caplin PS, McElheny SA, Church TR, Chitayat D, Hernández-Díaz S, Cohen LS. Reproductive safety of aripiprazole: data from the Massachusetts General Hospital National Pregnancy Registry for Atypical Antipsychotics. Arch Womens Ment Health. 2021;24(4):659-667. doi: 10.1007/s00737-021-01115-6. Epub 2021 Mar 12. Erratum in: Arch Womens Ment Health. 2021;24(4):669-670. doi: 10.1007/s00737-021-01133-4.	Dupl. 93PM
363	Freeman MP, Viguera AC, Góez-Mogollón L, Young AV, Caplin PS, McElheny SA, Church TR, Chitayat D, Hernández-Díaz S, Cohen LS. Reproductive safety of aripiprazole: data from the Massachusetts General Hospital National Pregnancy Registry for Atypical Antipsychotics. Arch Womens Ment Health. 2021;24(4):659-667. doi: 10.1007/s00737-021-01115-6. Epub 2021 Mar 12. Erratum in: Arch Womens Ment Health. 2021;24(4):669-670. doi: 10.1007/s00737-021-01133-4.	Dupl. 93PM

364	Tasaki M, Yasui-Furukori N, Yokoyama S, Shinozaki M, Sugawara N, Shimoda K. Hypoprolactinemia and hyperprolactinemia in male schizophrenia patients treated with aripiprazole and risperidone and their relationships with testosterone levels. Neuropsychopharmacol Rep. 2021;41(3):379-384. doi: 10.1002/npr2.12190. Epub 2021 Jun 29.	Dupl. 97PM
365	Román V, Adham N, Foley AG, Hanratty L, Farkas B, Lendvai B, Kiss B. Cariprazine alleviates core behavioral deficits in the prenatal valproic acid exposure model of autism spectrum disorder. Psychopharmacology (Berl). 2021;238(9):2381-2392. doi: 10.1007/s00213-021-05851-6. Epub 2021 Jul 15.	Dupl. 98PM
366	Liang CS, Su TP, Hsieh MH, Lee CS, Kuo J, Chiu NY, Chen PS, Yen YC, Bai YM. Taiwan Expert Consensus Recommendations for Switching to Aripiprazole Long-Acting Once-Monthly in Patients with Schizophrenia. J Pers Med. 2021;11(11):1198. doi: 10.3390/jpm11111198.	Dupl. 101PM
367	Matsuzaka Y, Noguchi M, Kanamura S, Maeda K, Hisano T, Tanaka D, Ando Y, Yamamoto T, Morimoto Y, Ozawa H, Otsuka T. Combination therapy of modified electroconvulsive therapy and long-acting injectable aripiprazole for dopamine supersensitivity psychosis: a case report. Neurocase. 2022;28(3):310-313. doi: 10.1080/13554794.2022.2114838. Epub 2022 Aug 21.	No preg/Lact
368	Rogóż Z, Kamińska K, Lech MA, Lorenc-Koci E. N-Acetylcysteine and Aripiprazole Improve Social Behavior and Cognition and Modulate Brain BDNF Levels in a Rat Model of Schizophrenia. Int J Mol Sci. 2022;23(4):2125. doi: 10.3390/ijms23042125.	Dupl. 104PM
369	Tsou HL, Hsu TW, Liang CS. Do Not Panic About Prenatal Exposure to Antipsychotics Except for Aripiprazole? JAMA Intern Med. 2022;182(10):1106. doi: 10.1001/jamainternmed.2022.3309. Refers to 105PM. Straub L, Hernández-Díaz S, Huybrechts KF. Do Not Panic About Prenatal Exposure to Antipsychotics Except for Aripiprazole?-Reply. JAMA Intern Med. 2022;182(10):1106-1107. doi: 10.1001/jamainternmed.2022.3312.	Opinion
370	Llorca PM, Nuss P, Fakra É, Alamome I, Drapier D, El Hage W, Jardri R, Mouchabac S, Rabbani M, Simon N, Vacheron MN, Azorin JM. Place of the partial dopamine receptor agonist aripiprazole in the management of schizophrenia in adults: a Delphi consensus study. BMC Psychiatry. 2022;22(1):364. doi: 10.1186/s12888-022-04008-9.	Dupl. 109PM
371	[Editorial]. Cariprazine (Vraylar) for Adjunctive Treatment of Depression. Medical Letter on Drugs and Therapeutics. 2023;65(1677):84-86	Opinion
372	Sahoo MK, Biswas H, Grover S. Safety Profile of Aripiprazole During Pregnancy and Lactation: Report of 2 Cases. Türk Psikiyatri Derg. 2023;34(2):133-135. doi: 10.5080/u26681.	Dupl. 122PM
373	Zohny SM, Habib MZ, Mohamad MI, Elayat WM, Elhossiny RM, El-Salam MFA, Hassan GAM, Aboul-Fotouh S. Memantine/Aripiprazole Combination Alleviates Cognitive Dysfunction in Valproic Acid Rat Model of Autism: Hippocampal CREB/BDNF Signaling and Glutamate Homeostasis. Neurotherapeutics. 2023;20(2):464-483. doi: 10.1007/s13311-023-01360-w. Epub 2023 Mar 14.	Dupl. 118PM
374	de Oliveira Ferreira E, Pessoa Gomes JM, Neves KRT, Lima FAV, de Barros Viana GS, de Andrade GM. Maternal treatment with aripiprazole prevents the development of a valproic acid-induced autism-like phenotype in juvenile male mice. Behav Pharmacol. 2023;34(2-3):154-168. doi: 10.1097/FBP.0000000000000718. Epub 2023 Feb 10.	Dupl. 117PM
375	Naughton S, O'Hara K, Nelson J, Keightley P. Aripiprazole, brexpiprazole, and cariprazine can affect milk supply: Advice to breastfeeding mothers. Australas Psychiatry. 2023;31(2):201-204. doi: 10.1177/10398562231159510. Epub 2023 Feb 24.	Dupl. 116PM
376	Herold R, Tényi T, Herold M, Tóth T. Cariprazine maintenance treatment during pregnancy – a case report. Front Psychiatry. 2024;15:1421395. doi: 10.3389/fpsyt.2024.1421395.	Dupl. 135PM
377	Jiang Y, Zhou L, Shen Y, Zhou Q, Ji Y, Zhu H. Safety assessment of Brexpiprazole: Real-world adverse event analysis from the FAERS database. J Affect Disord. 2024;346:223-229. doi: 10.1016/j.jad.2023.11.025. Epub 2023 Nov 11.	Dupl. 125PM
378	Pinci C, Bianciardi E, Sferra I, Castellani G, Santini R, Siracusano A, Niolu C. Switching from paliperidone palmitate 3-monthly long-acting injection to oral aripiprazole in a pregnant woman with schizophrenia: a case report and short review. Riv Psichiatr. 2024;59(2):75-79. doi: 10.1708/4259.42361.	Dupl. 131PM
379	Othman MA, Husni M, El-Din WAN, Salem AH, Sarwani N, Rashid A, Fadel R. Prenatal aripiprazole induces alterations of rat placenta: a histological, immunohistochemical and ultrastructural study. J Mol Histol. 2024;55(4):415-426. doi: 10.1007/s10735-024-10199-0. Epub 2024 May 7.	Dupl. 132PM
380	Konishi T, Kitahiro Y, Fujiwara N, Yamamoto K, Hashimoto M, Ito T, Itohara K, Fujioka K, Imafuku H, Otsuka I, Omura T, Yano I. Pharmacokinetics of Brexpiprazole, Quetiapine, Risperidone, and Its Active Metabolite Paliperidone in a Postpartum Woman and Her Baby. Ther Drug Monit. 2024;46(5):687-691. doi: 10.1097/FTD.0000000000001197. Epub 2024 Apr 4.	Dupl. 130PM
381	Teodorescu A, Dima L, Petric PS, Necula RM, Banciu R, Moga MA, Marian- Pavlenco A, Ifteni P. Treatment With Long-Acting Injectable Aripiprazole During Pregnancy in Bipolar Disorder: A Scoping Review. Am J Ther. 2024;31(6):e635-e644. doi: 10.1097/MJT.0000000000001773.	Dupl. 143PM
382	Jeong Y, Son S, Park J, Kim CY, Kim J. Antidepressant aripiprazole induces adverse effects on neural development during cortex organoid generation. Reprod Toxicol. 2025;133:108862. doi: 10.1016/j.reprotox.2025.108862. Epub 2025 Feb 17.	Dupl. 144PM
383	Nanjundaswamy MH, Shah A, Lotlikar S, Arasappa R, Ganjekar S, Thippeswamy H, Chandra PS, Desai G. Lactation-Related Side Effects of Aripiprazole: A Study From Perinatal Psychiatry Services in India. J Clin Psychopharmacol. 2025;45(3):258-266. doi: 10.1097/JCP.0000000000001997. Epub 2025 Apr 9.	Dupl. 149PM
384	Wang H, Li JT, Liu DN, Zhang XQ, Sun M, Zhang CC, Si TM, Su YA. Environmental enrichment improves deficits in hippocampal neuroplasticity and cognition in prenatally aripiprazole-exposed mouse offspring. Transl Psychiatry. 2025;15(1):102. doi: 10.1038/s41398-025-03335-1.	Dupl. 148PM
ClinicalTrials.gov 23.6.2025 Condition/Disease: Pregnancy, Other terms: Lactation, Treatment/Intervention, Aripiprazole → 1; Brexpiprazole → 1; Cariprazine → 0		
385	National Pregnancy Registry for Psychiatric Medications. NCT01246765. Lee Cohen, Bryn Rediger; Massachusetts General Hospital, Boston, Massachusetts, United States. Start: 2010-11-22, Last updated: 2023-11-13; Status: Recruiting	No Data
386	National Pregnancy Registry for Psychiatric Medications. NCT01246765. Lee Cohen, Bryn Rediger; Massachusetts General Hospital, Boston, Massachusetts, United States. Start: 2010-11-22, Last updated: 2023-11-13; Status: Recruiting	Dupl. 1ClTr

Included: 39
- Cases 24
- Studies 15 (11 from databases [retrospective], 1 small sample, 1 longitudinal, 2 prospective)

Excluded: 347
- Reviews 48
- No pregnancy/Lactation 45
- Animal 44
- Opinion 30
- Unfocused 10
- Unsuitable case 4
- No Data 2
- No DA partial agonists 1
- *In vitro* 1
- Unrelated 3
- Duplicates 159

Supplementary Table S2. PRISMA 2020 CheckList

PRISMA 2020 Checklist [45]

Section and Topic	Item #	Checklist item	Location where item is reported
**TITLE**			1
Title	1	Identify the report as a systematic review.	1
**ABSTRACT**			1
Abstract	2	See the PRISMA 2020 for Abstracts checklist.	1
**INTRODUCTION**			1-5
Rationale	3	Describe the rationale for the review in the context of existing knowledge.	4-
Objectives	4	Provide an explicit statement of the objective(s) or question(s) the review addresses.	5
**METHODS**			18
Eligibility criteria	5	Specify the inclusion and exclusion criteria for the review and how studies were grouped for the syntheses.	18
Information sources	6	Specify all databases, registers, websites, organisations, reference lists and other sources searched or consulted to identify studies. Specify the date when each source was last searched or consulted.	18
Search strategy	7	Present the full search strategies for all databases, registers and websites, including any filters and limits used.	18
Selection process	8	Specify the methods used to decide whether a study met the inclusion criteria of the review, including how many reviewers screened each record and each report retrieved, whether they worked independently, and if applicable, details of automation tools used in the process.	18
Data collection process	9	Specify the methods used to collect data from reports, including how many reviewers collected data from each report, whether they worked independently, any processes for obtaining or confirming data from study investigators, and if applicable, details of automation tools used in the process.	18
Data items	10a	List and define all outcomes for which data were sought. Specify whether all results that were compatible with each outcome domain in each study were sought (e.g. for all measures, time points, analyses), and if not, the methods used to decide which results to collect.	18
	10b	List and define all other variables for which data were sought (e.g. participant and intervention characteristics, funding sources). Describe any assumptions made about any missing or unclear information.	18
Study risk of bias assessment	11	Specify the methods used to assess risk of bias in the included studies, including details of the tool(s) used, how many reviewers assessed each study and whether they worked independently, and if applicable, details of automation tools used in the process.	N/A
Effect measures	12	Specify for each outcome the effect measure(s) (e.g. risk ratio, mean difference) used in the synthesis or presentation of results.	N/A
Synthesis methods	13a	Describe the processes used to decide which studies were eligible for each synthesis (e.g. tabulating the study intervention characteristics and comparing against the planned groups for each synthesis (item #5)).	18-19
	13b	Describe any methods required to prepare the data for presentation or synthesis, such as handling of missing summary statistics, or data conversions.	N/A
	13c	Describe any methods used to tabulate or visually display results of individual studies and syntheses.	18-19
	13d	Describe any methods used to synthesize results and provide a rationale for the choice(s). If meta-analysis was performed, describe the model(s), method(s) to identify the presence and extent of statistical heterogeneity, and software package(s) used.	N/A
	13e	Describe any methods used to explore possible causes of heterogeneity among study results (e.g. subgroup analysis, meta-regression).	N/A
	13f	Describe any sensitivity analyses conducted to assess robustness of the synthesized results.	N/A
Reporting bias assessment	14	Describe any methods used to assess risk of bias due to missing results in a synthesis (arising from reporting biases).	N/A
Certainty assessment	15	Describe any methods used to assess certainty (or confidence) in the body of evidence for an outcome.	N/A
**RESULTS**			5-17
Study selection	16a	Describe the results of the search and selection process, from the number of records identified in the search to the number of studies included in the review, ideally using a flow diagram.	5
	16b	Cite studies that might appear to meet the inclusion criteria, but which were excluded, and explain why they were excluded.	Suppl.
Study characteristics	17	Cite each included study and present its characteristics.	5-17
Risk of bias in studies	18	Present assessments of risk of bias for each included study.	N/A
Results of individual studies	19	For all outcomes, present, for each study: (a) summary statistics for each group (where appropriate) and (b) an effect estimate and its precision (e.g. confidence/credible interval), ideally using structured tables or plots.	N/A
Results of syntheses	20a	For each synthesis, briefly summarise the characteristics and risk of bias among contributing studies.	N/A
	20b	Present results of all statistical syntheses conducted. If meta-analysis was done, present for each the summary estimate and its precision (e.g. confidence/credible interval) and measures of statistical heterogeneity. If comparing groups, describe the direction of the effect.	N/A

Section and Topic	Item #	Checklist item	Location where item is reported
	20c	Present results of all investigations of possible causes of heterogeneity among study results.	N/A
	20d	Present results of all sensitivity analyses conducted to assess the robustness of the synthesized results.	N/A
Reporting biases	21	Present assessments of risk of bias due to missing results (arising from reporting biases) for each synthesis assessed.	N/A
Certainty of evidence	22	Present assessments of certainty (or confidence) in the body of evidence for each outcome assessed.	N/A
**DISCUSSION**			17-18, 19
Discussion	23a	Provide a general interpretation of the results in the context of other evidence.	17-18, 19
	23b	Discuss any limitations of the evidence included in the review.	17-18
	23c	Discuss any limitations of the review processes used.	17-18
	23d	Discuss implications of the results for practice, policy, and future research.	17-18, 19
**OTHER INFORMATION**			
Registration and protocol	24a	Provide registration information for the review, including register name and registration number, or state that the review was not registered.	19
	24b	Indicate where the review protocol can be accessed, or state that a protocol was not prepared.	19-20
	24c	Describe and explain any amendments to information provided at registration or in the protocol.	N/A
Support	25	Describe sources of financial or non-financial support for the review, and the role of the funders or sponsors in the review.	19-20
Competing interests	26	Declare any competing interests of review authors.	19-20
Availability of data, code and other materials	27	Report which of the following are publicly available and where they can be found: template data collection forms; data extracted from included studies; data used for all analyses; analytic code; any other materials used in the review.	N/A

Supplementary Table S3. Quality assessment of included studies.

JBI Critical Appraisal Checklist for Case Reports

Study: Mendhekar et al., 2006a [49]				
Question	Yes	No	Unclear	Not applicable
1. Were patient's demographic characteristics clearly described?	X			
2. Was the patient's history clearly described and presented as a timeline?	X			
3. Was the current clinical condition of the patient on presentation clearly described?	X			
4. Were diagnostic tests or assessment methods and the results clearly described?	X			
5. Was the intervention(s) or treatment procedure(s) clearly described?	X			
6. Was the post-intervention clinical condition clearly described?	X			
7. Were adverse events (harms) or unanticipated events identified and described?	X			
8. Does the case report provide takeaway lessons?	X			
Study: Mendhekar et al., 2006b [50]				
Question	Yes	No	Unclear	Not applicable
1. Were patient's demographic characteristics clearly described?	X			
2. Was the patient's history clearly described and presented as a timeline?	X			
3. Was the current clinical condition of the patient on presentation clearly described?	X			
4. Were diagnostic tests or assessment methods and the results clearly described?	X			
5. Was the intervention(s) or treatment procedure(s) clearly described?	X			
6. Was the post-intervention clinical condition clearly described?	X			
7. Were adverse events (harms) or unanticipated events identified and described?	X			
8. Does the case report provide takeaway lessons?	X			
Study: Mervak et al., 2008 [51]				
Question	Yes	No	Unclear	Not applicable
1. Were patient's demographic characteristics clearly described?	X			
2. Was the patient's history clearly described and presented as a timeline?	X			
3. Was the current clinical condition of the patient on presentation clearly described?	X			
4. Were diagnostic tests or assessment methods and the results clearly described?	X			
5. Was the intervention(s) or treatment procedure(s) clearly described?	X			
6. Was the post-intervention clinical condition clearly described?	X			
7. Were adverse events (harms) or unanticipated events identified and described?	X			
8. Does the case report provide takeaway lessons?	X			
Study: Lutz et al., 2010 [52]				
Question	Yes	No	Unclear	Not applicable
1. Were patient's demographic characteristics clearly described?	X			
2. Was the patient's history clearly described and presented as a timeline?	X			
3. Was the current clinical condition of the patient on presentation clearly described?	X			
4. Were diagnostic tests or assessment methods and the results clearly described?	X			
5. Was the intervention(s) or treatment procedure(s) clearly described?	X			
6. Was the post-intervention clinical condition clearly described?	X			
7. Were adverse events (harms) or unanticipated events identified and described?	X			

8. Does the case report provide takeaway lessons?	X			
Study: Nguyen et al., 2011 [53]				
Question	Yes	No	Unclear	Not applicable
9. Were patient's demographic characteristics clearly described?	X			
10. Was the patient's history clearly described and presented as a timeline?	X			
11. Was the current clinical condition of the patient on presentation clearly described?	X			
12. Were diagnostic tests or assessment methods and the results clearly described?	X			
13. Was the intervention(s) or treatment procedure(s) clearly described?	X			
14. Was the post-intervention clinical condition clearly described?	X			
15. Were adverse events (harms) or unanticipated events identified and described?	X			
16. Does the case report provide takeaway lessons?	X			
Study: Watanabe et al., 2011 [54]				
Question	Yes	No	Unclear	Not applicable
9. Were patient's demographic characteristics clearly described?	X			
10. Was the patient's history clearly described and presented as a timeline?	X			
11. Was the current clinical condition of the patient on presentation clearly described?	X			
12. Were diagnostic tests or assessment methods and the results clearly described?	X			
13. Was the intervention(s) or treatment procedure(s) clearly described?	X			
14. Was the post-intervention clinical condition clearly described?	X			
15. Were adverse events (harms) or unanticipated events identified and described?	X			
16. Does the case report provide takeaway lessons?	X			
Study: Gentile et al., 2011 [55]				
Question	Yes	No	Unclear	Not applicable
9. Were patient's demographic characteristics clearly described?	X			
10. Was the patient's history clearly described and presented as a timeline?	X			
11. Was the current clinical condition of the patient on presentation clearly described?	X			
12. Were diagnostic tests or assessment methods and the results clearly described?	X			
13. Was the intervention(s) or treatment procedure(s) clearly described?	X			
14. Was the post-intervention clinical condition clearly described?	X			
15. Were adverse events (harms) or unanticipated events identified and described?	X			
16. Does the case report provide takeaway lessons?	X			
Study: Widschwendter and Hofer, 2012 [56]				
Question	Yes	No	Unclear	Not applicable
9. Were patient's demographic characteristics clearly described?	X			
10. Was the patient's history clearly described and presented as a timeline?	X			
11. Was the current clinical condition of the patient on presentation clearly described?	X			
12. Were diagnostic tests or assessment methods and the results clearly described?	X			
13. Was the intervention(s) or treatment procedure(s) clearly described?	X			
14. Was the post-intervention clinical condition clearly described?	X			
15. Were adverse events (harms) or unanticipated events identified and described?	X			
16. Does the case report provide takeaway lessons?	X			
Study: Wakil et al., 2013 [57]				
Question	Yes	No	Unclear	Not applicable
17. Were patient's demographic characteristics clearly described?	X			
18. Was the patient's history clearly described and presented as a timeline?	X			
19. Was the current clinical condition of the patient on presentation clearly described?	X			
20. Were diagnostic tests or assessment methods and the results clearly described?	X			
21. Was the intervention(s) or treatment procedure(s) clearly described?	X			
22. Was the post-intervention clinical condition clearly described?	X			
23. Were adverse events (harms) or unanticipated events identified and described?	X			
24. Does the case report provide takeaway lessons?	X			
Study: Windhager et al., 2014 [84]				
Question	Yes	No	Unclear	Not applicable
17. Were patient's demographic characteristics clearly described?	X			
18. Was the patient's history clearly described and presented as a timeline?	X			
19. Was the current clinical condition of the patient on presentation clearly described?	X			
20. Were diagnostic tests or assessment methods and the results clearly described?	X			
21. Was the intervention(s) or treatment procedure(s) clearly described?	X			
22. Was the post-intervention clinical condition clearly described?	X			
23. Were adverse events (harms) or unanticipated events identified and described?	X			
24. Does the case report provide takeaway lessons?	X			
Study: Pirec et al., 2014 [59]				
Question	Yes	No	Unclear	Not applicable
17. Were patient's demographic characteristics clearly described?	X			
18. Was the patient's history clearly described and presented as a timeline?	X			
19. Was the current clinical condition of the patient on presentation clearly described?	X			
20. Were diagnostic tests or assessment methods and the results clearly described?	X			
21. Was the intervention(s) or treatment procedure(s) clearly described?	X			

22. Was the post-intervention clinical condition clearly described?	X			
23. Were adverse events (harms) or unanticipated events identified and described?	X			
24. Does the case report provide takeaway lessons?	X			
Study: Nordeng et al., 2014 [60]				
Question	Yes	No	Unclear	Not applicable
17. Were patient's demographic characteristics clearly described?	X			
18. Was the patient's history clearly described and presented as a timeline?	X			
19. Was the current clinical condition of the patient on presentation clearly described?	X			
20. Were diagnostic tests or assessment methods and the results clearly described?	X			
21. Was the intervention(s) or treatment procedure(s) clearly described?	X			
22. Was the post-intervention clinical condition clearly described?	X			
23. Were adverse events (harms) or unanticipated events identified and described?	X			
24. Does the case report provide takeaway lessons?	X			
Study: Frew, 2015 [61]				
Question	Yes	No	Unclear	Not applicable
25. Were patient's demographic characteristics clearly described?	X			
26. Was the patient's history clearly described and presented as a timeline?	X			
27. Was the current clinical condition of the patient on presentation clearly described?	X			
28. Were diagnostic tests or assessment methods and the results clearly described?	X			
29. Was the intervention(s) or treatment procedure(s) clearly described?	X			
30. Was the post-intervention clinical condition clearly described?	X			
31. Were adverse events (harms) or unanticipated events identified and described?	X			
32. Does the case report provide takeaway lessons?	X			
Study: Morin & Chevalier, 2017 [62]				
Question	Yes	No	Unclear	Not applicable
25. Were patient's demographic characteristics clearly described?	X			
26. Was the patient's history clearly described and presented as a timeline?	X			
27. Was the current clinical condition of the patient on presentation clearly described?	X			
28. Were diagnostic tests or assessment methods and the results clearly described?	X			
29. Was the intervention(s) or treatment procedure(s) clearly described?	X			
30. Was the post-intervention clinical condition clearly described?	X			
31. Were adverse events (harms) or unanticipated events identified and described?	X			
32. Does the case report provide takeaway lessons?	X			
Study: Yskes et al., 2018 [63]				
Question	Yes	No	Unclear	Not applicable
25. Were patient's demographic characteristics clearly described?	X			
26. Was the patient's history clearly described and presented as a timeline?	X			
27. Was the current clinical condition of the patient on presentation clearly described?	X			
28. Were diagnostic tests or assessment methods and the results clearly described?	X			
29. Was the intervention(s) or treatment procedure(s) clearly described?	X			
30. Was the post-intervention clinical condition clearly described?	X			
31. Were adverse events (harms) or unanticipated events identified and described?	X			
32. Does the case report provide takeaway lessons?	X			
Study: Ballester-Gracia et al., 2019 [64]				
Question	Yes	No	Unclear	Not applicable
25. Were patient's demographic characteristics clearly described?	X			
26. Was the patient's history clearly described and presented as a timeline?	X			
27. Was the current clinical condition of the patient on presentation clearly described?	X			
28. Were diagnostic tests or assessment methods and the results clearly described?	X			
29. Was the intervention(s) or treatment procedure(s) clearly described?	X			
30. Was the post-intervention clinical condition clearly described?	X			
31. Were adverse events (harms) or unanticipated events identified and described?	X			
32. Does the case report provide takeaway lessons?	X			
Study: Walker et al., 2019 [65]				
Question	Yes	No	Unclear	Not applicable
33. Were patient's demographic characteristics clearly described?			X	
34. Was the patient's history clearly described and presented as a timeline?	X			
35. Was the current clinical condition of the patient on presentation clearly described?			X	
36. Were diagnostic tests or assessment methods and the results clearly described?	X			
37. Was the intervention(s) or treatment procedure(s) clearly described?	X			
38. Was the post-intervention clinical condition clearly described?	X			
39. Were adverse events (harms) or unanticipated events identified and described?	X			
40. Does the case report provide takeaway lessons?	X			
Study: Solé et al., 2020 [66]				
Question	Yes	No	Unclear	Not applicable
33. Were patient's demographic characteristics clearly described?	X			
34. Was the patient's history clearly described and presented as a timeline?			X	
35. Was the current clinical condition of the patient on presentation clearly described?	X			

36. Were diagnostic tests or assessment methods and the results clearly described?			X	
37. Was the intervention(s) or treatment procedure(s) clearly described?	X			
38. Was the post-intervention clinical condition clearly described?		X		
39. Were adverse events (harms) or unanticipated events identified and described?	X			
40. Does the case report provide takeaway lessons?	X			
Study: Komaroff, 2021 [67]				
Question	Yes	No	Unclear	Not applicable
33. Were patient’s demographic characteristics clearly described?	X			
34. Was the patient’s history clearly described and presented as a timeline?	X			
35. Was the current clinical condition of the patient on presentation clearly described?	X			
36. Were diagnostic tests or assessment methods and the results clearly described?	X			
37. Was the intervention(s) or treatment procedure(s) clearly described?	X			
38. Was the post-intervention clinical condition clearly described?	X			
39. Were adverse events (harms) or unanticipated events identified and described?	X			
40. Does the case report provide takeaway lessons?	X			
Study: Fernández-Abascal et al., 2021 [68]				
Question	Yes	No	Unclear	Not applicable
33. Were patient’s demographic characteristics clearly described?	X			
34. Was the patient’s history clearly described and presented as a timeline?	X			
35. Was the current clinical condition of the patient on presentation clearly described?	X			
36. Were diagnostic tests or assessment methods and the results clearly described?	X			
37. Was the intervention(s) or treatment procedure(s) clearly described?	X			
38. Was the post-intervention clinical condition clearly described?	X			
39. Were adverse events (harms) or unanticipated events identified and described?	X			
40. Does the case report provide takeaway lessons?	X			
Study: Sahoo et al., 2023 [69]				
Question	Yes	No	Unclear	Not applicable
41. Were patient’s demographic characteristics clearly described?	X			
42. Was the patient’s history clearly described and presented as a timeline?	X			
43. Was the current clinical condition of the patient on presentation clearly described?	X			
44. Were diagnostic tests or assessment methods and the results clearly described?	X			
45. Was the intervention(s) or treatment procedure(s) clearly described?	X			
46. Was the post-intervention clinical condition clearly described?	X			
47. Were adverse events (harms) or unanticipated events identified and described?	X			
48. Does the case report provide takeaway lessons?	X			
Study: Konishi et al., 2024 [70]				
Question	Yes	No	Unclear	Not applicable
41. Were patient’s demographic characteristics clearly described?	X			
42. Was the patient’s history clearly described and presented as a timeline?	X			
43. Was the current clinical condition of the patient on presentation clearly described?	X			
44. Were diagnostic tests or assessment methods and the results clearly described?	X			
45. Was the intervention(s) or treatment procedure(s) clearly described?	X			
46. Was the post-intervention clinical condition clearly described?	X			
47. Were adverse events (harms) or unanticipated events identified and described?	X			
48. Does the case report provide takeaway lessons?	X			
Study: Pinci et al., 2024 [71]				
Question	Yes	No	Unclear	Not applicable
41. Were patient’s demographic characteristics clearly described?	X			
42. Was the patient’s history clearly described and presented as a timeline?	X			
43. Was the current clinical condition of the patient on presentation clearly described?	X			
44. Were diagnostic tests or assessment methods and the results clearly described?	X			
45. Was the intervention(s) or treatment procedure(s) clearly described?	X			
46. Was the post-intervention clinical condition clearly described?	X			
47. Were adverse events (harms) or unanticipated events identified and described?	X			
48. Does the case report provide takeaway lessons?	X			
Study: Herold et al., 2024 [72]				
Question	Yes	No	Unclear	Not applicable
41. Were patient’s demographic characteristics clearly described?	X			
42. Was the patient’s history clearly described and presented as a timeline?	X			
43. Was the current clinical condition of the patient on presentation clearly described?	X			
44. Were diagnostic tests or assessment methods and the results clearly described?	X			
45. Was the intervention(s) or treatment procedure(s) clearly described?	X			
46. Was the post-intervention clinical condition clearly described?	X			
47. Were adverse events (harms) or unanticipated events identified and described?	X			
48. Does the case report provide takeaway lessons?	X			

[46] Moola, S.; Munn, Z.; Tufanaru, C.; Aromataris, E.; Sears, K.; Sfetcu, R.; Currie, M.; Qureshi, R.; Mattis, P.; Lisy, K.; Mu, P.-F. Chapter 7: Systematic reviews of etiology and risk. In: Aromataris, E.; Munn, Z. (Editors). *JBI Manual for Evidence Synthesis*. Joanna Briggs Institute, Adelaide, South Australia, 2020. Available from https://synthesismanual.jbi.global.

[47] Gagnier, J.J.; Kienle, G.; Altman, D.G.; Moher, D.; Sox, H.; Riley, D.; CARE Group. The CARE guidelines: consensus-based clinical case reporting guideline development. *Headache* **2013**, *53*(10), 1541-1547. doi: 10.1111/head.12246.

Cochrane tool for assessing risk of bias in randomised trials (RoB-2)

Study	Confounding	Measurement of exposure	Participant selection	Post-exposure intervention	Missing data	Measurement of outcome	Selection of reported results	Overall Risk of Bias
Maňáková & Hubičková, 2011 [73]								
Bellet et al., 2015 [74]								
Montastruc et al., 2016 [75]								
Park et al., 2017 [76]								
Sakai et al., 2017 [77]								
Westin et al., 2018 [78]								
Park et al., 2018 [79]								
Galbally et al., 2018 [80]								
Freeman et al., 2021 [81]								
Straub et al., 2022 [82]								
Jiang et al., 2024 [83]								
Ishikawa et al., 2024 [84]								
Zheng et al., 2024 [85]								
Cho et al., 2025 [86]								
Nanjundaswamy et al., 2025 [87]								

Colour code: Red, high risk of bias; Yellow, some concerns; Green, low risk of bias.

[48] Sterne, J.A.C.; Savović, J.; Page, M.J.; Elbers, R.G.; Blencowe, N.S.; Boutron, I.; Cates, C.J.; Cheng, H.-Y.; Corbett, M.S.; Eldridge, S.M.; Hernán, M.A.; Hopewell, S.; Hróbjartsson, A.; Junqueira, D.R.; Jüni, P.; Kirkham, J.J.; Lasserson, T.; Li, T.; McAleenan, A.; Reeves, B.C.; Shepperd, S.; Shrier, I.; Stewart, L.A.; Tilling, K.; White, I.R.; Whiting, P.F.; Higgins, J.P.T. RoB 2: a revised tool for assessing risk of bias in randomised trials. *BMJ* **2019**, *366*, l4898.
